# Squaraine Dyes for Organic Photomultiplication Photodetectors with 220% External Quantum Efficiency at 1240 nm

**DOI:** 10.1002/advs.202502320

**Published:** 2025-04-03

**Authors:** Joshua Csucker, Elodie Didier, João Pedro Ferreira Assunção, Daniel Rentsch, Radha Kothandaraman, Dominik Bachmann, Ivan Shorubalko, Frank Nüesch, Roland Hany, Michael Bauer

**Affiliations:** ^1^ Empa Swiss Federal Laboratories for Materials Science and Technology Laboratory for Functional Polymers Dübendorf CH‐8600 Switzerland; ^2^ Institute of Chemistry and Chemical Engineering Ecole Polytechnique Fédérale de Lausanne EPFL Station 12 Lausanne CH‐1015 Switzerland; ^3^ Institute of Materials Science and Engineering Ecole Polytechnique Fédérale de Lausanne EPFL Station 12 Lausanne CH‐1015 Switzerland; ^4^ Empa Swiss Federal Laboratories for Materials Science and Technology Laboratory for Thin Films and Photovoltaics Dübendorf CH‐8600 Switzerland; ^5^ Empa Swiss Federal Laboratories for Materials Science and Technology Transport at Nanoscale Interfaces Laboratory Dübendorf CH‐8600 Switzerland

**Keywords:** gain photodetector, near infrared, organic photodetector, photomultiplication, squaraine dye

## Abstract

Near‐infrared (NIR) light detection at wavelengths λ > 1100 nm is essential in modern science and technology. Emerging organic semiconductors are promising for solution‐processed, flexible, and large‐area NIR organic photodetectors (OPDs), but only a few organic chromophores with peak absorption beyond the silicon bandgap are available. Furthermore, the external quantum efficiency (EQE) and specific detectivity (D^*^) of NIR OPDs are restricted by insufficient exciton dissociation and high dark/noise current. Here, the combination of strong electron‐accepting and ‐donating groups is used to synthesize a selection of novel NIR squaraine dyes with superior redshifted absorptions, peaking at 1165 nm in solution and extending to 1240 nm in a blend film. To overcome the tradeoff between long wavelength absorption and high photoresponse, NIR photons are detected utilizing a gain OPD design, where photomultiplication occurs via squaraine hole trap‐induced injection of external charges. The OPD can achieve an EQE of 220% at 1240 nm and still maintains 25% in the absorption tail at 1400 nm, thereby surpassing existing NIR OPDs in a broad wavelength range beyond 1100 nm. The measured maximum D^*^ equals 10^9^ Jones at 1240 nm, and the detectivity estimated from the shot noise is ≈10^11^ Jones, independent of the bias voltage.

## Graphical abstract

The synthesis of a range of squaraine dyes is reported with maximum absorption in solution above 1100 nm. Near‐infrared sensitive organic photomultiplication photodetectors are developed. Photomultiplication occurs via squaraine hole trap‐induced injection of electrons from the external circuit. Photodetectors achieve an external quantum efficiency of 220% at 1240 nm.

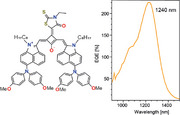

## 1 Introduction

Near‐infrared (NIR) photodetectors (PDs) enable broad applications ranging from industrial product monitoring and sorting to machine automation systems and are typically made of inorganic sensor materials, such as silicon (up to 1100 nm) or indium gallium arsenide that is sensitive in the wavelength range ≈ 800–1800 nm.^[^ ^1^, ^2^, ^3^ ^]^ However, emerging technologies in environmental monitoring and medical diagnostics, consumer applications, or wearable health monitoring require low‐cost, large‐area, lightweight, and mechanically compliant NIR PDs. For this, NIR‐sensitive organic semiconductors show promising application perspective due to their tunable optical and chemical properties, simple integration with read‐out circuits in imagers, and compatibility with solution‐processing of NIR organic PDs (OPDs) at low temperatures.^[^ ^4^, ^5^, ^6^ ^]^

Important figures of merit for any PD include a fast temporal response, a large dynamic range to precisely detect signals of various light intensity, as well as a high external quantum efficiency (EQE), which is the ratio of the number of collected electrical charges and the incident photons. The key performance metric for the sensitivity of a PD to weak light intensity is the specific detectivity D^*^ [cm Hz^½^ W^−1^ or Jones],^[^ ^4^, ^7^, ^8^, ^9^ ^]^ which is proportional to the ratio of photocurrent response and noise current spectral density.

Recently, several NIR OPDs have been reported with maximum sensitivity in the range 900–1100 nm that have a D^*^ within reach of typical commercial silicon PDs, i.e., D^*^ ≈ 10^13^ Jones. Therefore, polymers or small molecules have been explored in order to reduce the material's optical bandgap by extending the conjugation length or introducing donor‐acceptor push‐pull structures, including quinoidal building blocks.^[^ ^10^, ^11^, ^12^, ^13^, ^14^, ^15^ ^]^ Also, the family of non‐fullerene acceptors has great potential for NIR and narrowband light detection, with absorption edges at ≈1100 nm.^[^ ^7^, ^10^, ^16^, ^17^ ^]^

However, the development of NIR OPDs with reasonable peak efficiency beyond 1100 nm that rely on direct photon absorption over the material's bandgap, i.e., not utilizing optical cavity structures,^[^ ^4^, ^18^ ^]^ has remained challenging. First, the device limiting factors for D^*^ with decreasing optical gap are high non‐radiative recombination and poor exciton dissociation,^[^ ^12^, ^14^, ^19^, ^20^ ^]^ consistent with the energy gap law–as well as thermal and trap state‐mediated generation of charge carriers resulting in high dark/noise current.^[^ ^10^, ^11^, ^12^, ^17^, ^21^, ^22^ ^]^ In addition, the library of organic semiconducting materials with maximum absorption above the cutoff wavelength of silicon is very small.^[^ ^23^, ^24^, ^25^ ^]^ Examples of organic materials in NIR OPDs with peak response ranging from 1200 nm to 1400 nm include narrow‐bandgap conjugated polymers,^[^ ^13^, ^19^, ^22^, ^26^, ^27^, ^28^ ^]^ heptamethine cyanine dyes or a porphyrin‐type dimer,^[^ ^29^, ^30^ ^]^ with excellent values for the EQE ≈ 20% and D^*^ = 3 × 10^10^ Jones at 1200 nm measured in reference. ^[^ ^27^ ^]^ In general, reported EQE values drop to below 10% at even longer wavelengths, and typical measured specific detectivities are in the order of D^*^ = 10^8^–10^9^ Jones.^[^ ^14^, ^20^, ^22^, ^26^, ^30^ ^]^ OPD photoresponse below 1 eV was also possible in cases where the long wavelength absorption is not intrinsic to the molecular structure but has been achieved via a redshift due to aggregation in the film.^[^ ^14^, ^31^ ^]^

Squaraine (SQ) dyes are an interesting class of functional chromophores due to their strong red and NIR absorption in a narrow wavelength range.^[^ ^24^ ^]^ SQs have good photo‐physical stability, and they have been used in a variety of applications in bioimaging, chemical sensing, and optoelectronic devices,^[^ ^32^, ^33^, ^34^ ^]^ including NIR OPDs.^[^ ^25^, ^35^, ^36^ ^]^ SQs are polymethine dyes that consist of 1,3‐donor‐substituted derivatives of a central electron‐accepting squaric acid core.^[^ ^23^, ^37^ ^]^ It is known that the combination of strong donor with acceptor units results in a bathochromic shift of the absorption.^[^ ^37^, ^38^, ^39^ ^]^ For example, the symmetrical benz[*c,d*]indole‐capped SQ dye absorbs at 900 nm, and the absorption shifts to 1014 nm when introducing the dicyanomethylene group at the squaric acid core in combination with thienyl‐substituted benzindoles.^[^ ^25^, ^40^ ^]^

Exploring the donor‐acceptor‐donor concept in SQs, we here report the synthesis of a panel of SQ dyes with absorption maxima beyond 1000 nm, reaching 1165 nm for the dye that incorporates the rhodanine acceptor unit with diphenylamine‐substituted benzindole groups. We tested dyes in so‐called photomultiplication OPDs that exhibit a gain.^[^ ^4^, ^31^, ^41^, ^42^, ^43^, ^44^ ^]^ Photomultiplication OPDs operate via photogenerated trap‐induced injection of multiple secondary charges from the external circuit and therefore the EQE can exceed 100%.^[^ ^45^, ^46^, ^47^ ^]^ After device optimization we evaluated photodetector properties, including spectral response in the NIR, noise current, response time, and linear dynamic range. Optimized OPDs showed peak EQE = 220% and measured D^*^ reached 10^9^ Jones at 1240 nm.

## 2 Results and Discussion

### 2.1 Squaraine Dye Synthesis and Molecular Properties

**Scheme 1:**
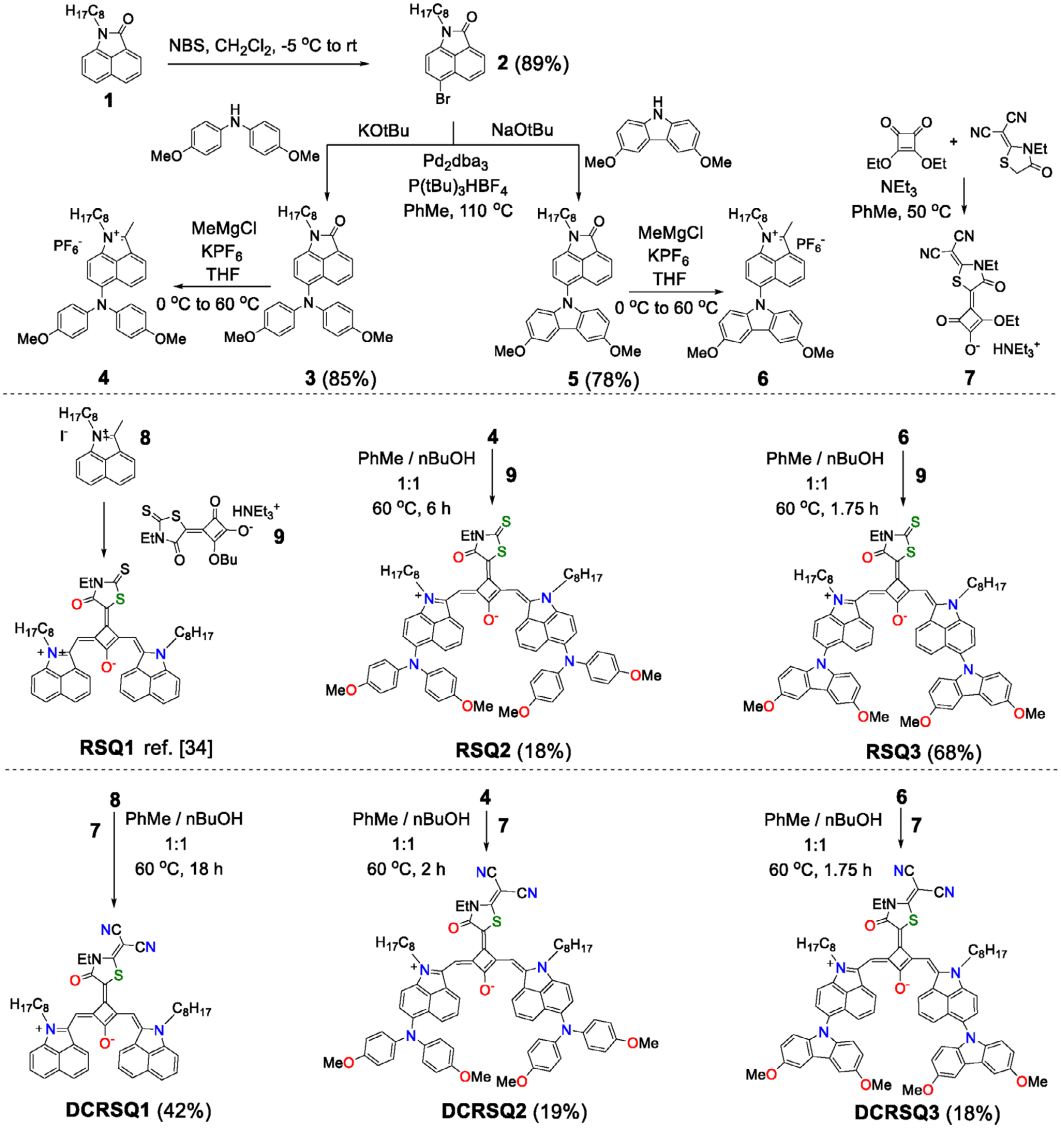
Synthesis of squaraine dyes. Indicated yields refer to the last synthetic step.

Five new acceptor‐functionalized SQs with symmetrical donor units were conceptualized and synthesized for this work (**Scheme** **1**). Dye preparation followed routes reported by Strassel et al.^[^ ^25^ ^]^ and Wang et al.,^[^ ^39^ ^]^ albeit with optimization work of key synthesis steps. The Buchwald‐Hartwig C‐N cross coupling between **2** and bis(4‐methoxyphenyl)amine or 3,6‐dimethoxycarbazole delivered **3** and **5**. The choice of base was critical; KOtBu provided successful conversion to **3** (85%), whereas NaOtBu was required as a base to deliver **5** in acceptable (78%) yield.

The amides were converted to iminium salts **4** and **6** by the addition of methyl magnesium chloride. Isolating the cations as PF_6_^−^ salt was essential for successful condensation with the rhodanine squarate **9** toward **RSQ2** and **RSQ3**, as attempts with ClO_4_^−^ or I^−^ failed, likely due to their oxidative properties. Heating the reaction above 60 °C led to the thermal decomposition of the squaric acid ester starting material. The isolated yield (18%) of **RSQ2** from four batches was reproducible but moderate. In a similar manner, **DCRSQ2** and **DCRSQ3** were obtained by coupling **4** and **6** with the synthesized (crude yield 83%) dicyano‐rhodanine squarate **7**.

**RSQ1** was synthesized before^[^ ^36^ ^]^ and its structural and molecular properties are included here for the sake of completeness. **DCRSQ1** was synthesized as reported for **RSQ1** via condensation of the iminium iodide **8** with **7** in a mixture of toluene and *n*‐butanol, limiting the temperature to 60 °C. For the synthesis of the dicyano‐rhodanine squaraine dyes, in situ formed water was removed by adding an activated molecular sieve to the reaction mixture. Experimental details for the synthetic procedure of all dyes are compiled in the Supporting Information.

The structural characterization of new SQ dyes was made using NMR and high‐resolution MS (Supporting Information). Thermal gravimetric analysis (TGA) under nitrogen atmosphere (Figure S1, Supporting Information) showed that dyes are stable up to ≈200 °C, and temperatures at 5% mass loss varied between 218 °C (for **RSQ2**) and 270 °C (for **RSQ3**). This agrees with reported values for related SQs with different donor substituents, indicating the lability of the central four‐membered ring.^[^ ^25^, ^48^ ^]^

**Figure 1:**
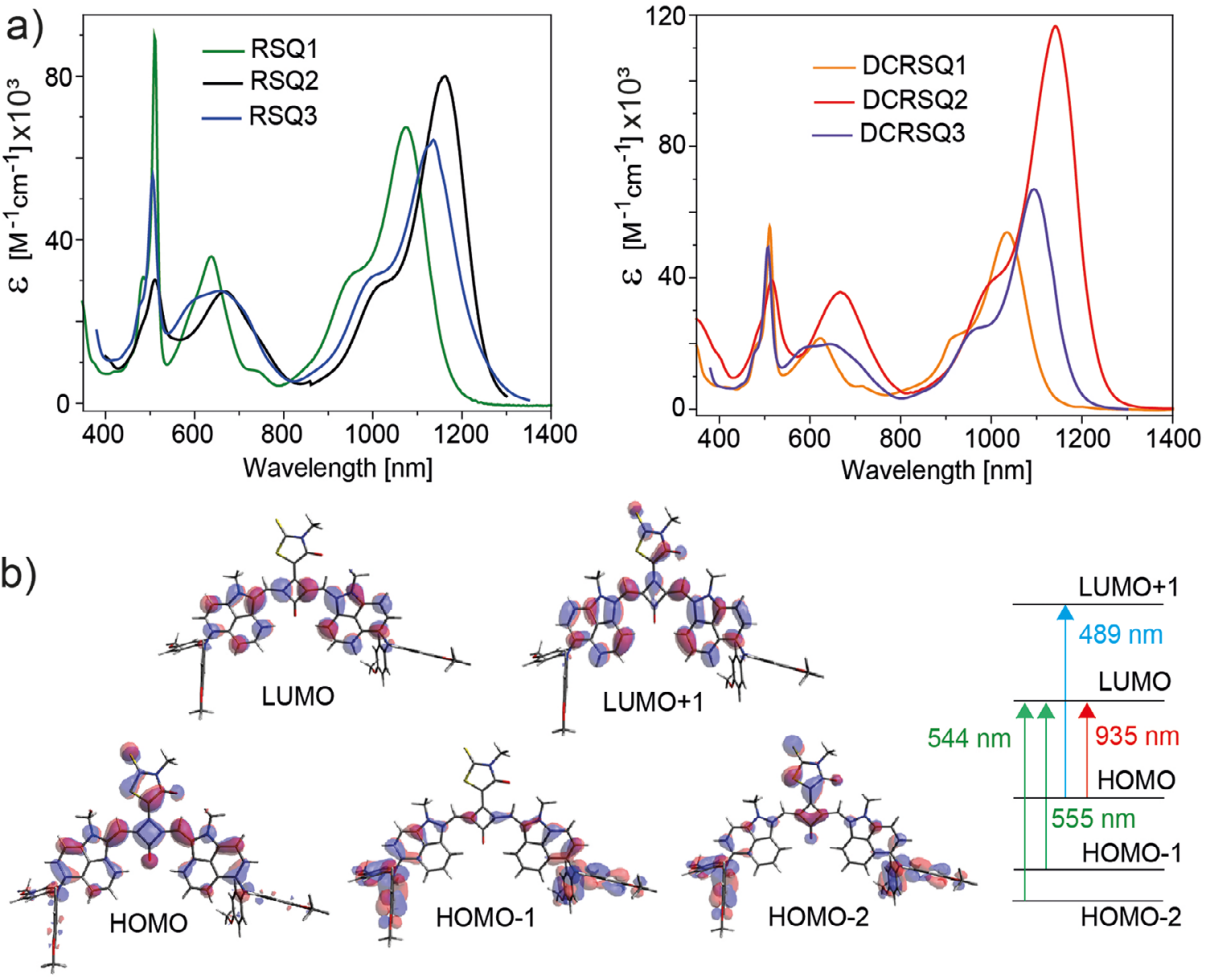
a) Absorption spectra of squaraine dyes in toluene. b) Frontier molecular orbitals and calculated optical transitions for **RSQ2**.

Absorption spectra in solution displayed for all dyes three absorption bands at 510 nm, at ≈650 nm, and in the NIR (**Figure** **1**a). Typical for the main optical transition of SQs is the very high extinction coefficient (**Table** **1**).^[^ ^37^ ^]^ The NIR band shows a hypsochromic shift in absorption with increasing solvent polarity (negative solvatochromism, Figure S2, Supporting Information), but the absorption wavelength at 510 nm did not change when comparing spectra in toluene and dichloromethane (Figure S3, Supporting Information). In the dye series, the rhodanine acceptor resulted in a larger redshift than dicyano‐rhodanine. Also, substitution at the benzindole moiety lowered the optical bandgap, and the diphenylamine unit was a stronger donor than carbazole. The most red‐shifted absorption (for any SQ reported so far) at 1165 nm was measured for **RSQ2** which is composed of diphenylamine‐substituted benzindole donors and the rhodanine‐substituted acceptor.

**Table 1:** Absorption and electrochemical data for squaraine dyes.

	λ_max,sol_ [nm]^a)^	ε_max_ [M^−1^cm^−1^] (f_exp_)^b)^	S1 (calc.) [nm] (f_calc_)^c)^, ^d)^	S2 (calc.) [nm] (f_calc_)^c)^, ^f)^	S3 (calc.) [nm] (f_calc_)^c)^, ^g)^	λ_max,film_ [nm]^h)^	E_HOMO_ [eV]^i)^	E_LUMO_ [eV]^j)^
**RSQ1**	1072 (1015)	68 000 (0.33)	851 (1.32)	477 (0.58)	466 (0.90)	1071	−4.93	−4.10
**RSQ2**	1165 (1118)	80 000 (0.33)	935 (1.65); 908^e)^	555 (0.44); 544 (0.20)	489 (0.58); 487^e)^	1182	−4.74	−4.07
**RSQ3**	1134 (1074)	64 000 (0.33)	892 (1.45)	536 (0.16); 521 (0.22)	476 (0.85)	1156	−5.00	−4.23
**DCRSQ1**	1035 (990)	54 000 (0.25)	823 (1.41)	461 (0.49)	465 (1.03)	900; 1050	−5.00	−4.11
**DCRSQ2**	1142 (1106)	117 000 (0.48)	921 (1.73); 900^e)^	560 (0.53)	488 (0.62); 488^e)^	1160	−4.75	−4.04
**DCRSQ3**	1096 (1050)	67 000 (0.28)	864 (1.54)	541 (0.18)	473 (0.96)	1110	−5.03	−4.20

^a)^ Measured in toluene. In bracket absorption maxima in dichloromethane;

^b)^ Measured in toluene. In bracket experimental oscillator strength^[^ ^49^ ^]^;

^c)^ Prominent calculated excited states in toluene, oscillator strength (f, > 0.15) in bracket;

^d)^ HOMO → LUMO;

^e)^ In dichloromethane;

^f)^ Predominantly HOMO‐1 → LUMO. For **RSQ2** and **RSQ3**, S2 is composed of HOMO‐1 and HOMO‐2 → LUMO;

^g)^ HOMO → LUMO+1;

^h)^ Spin‐coated from a chloroform solution onto a glass substrate;

^i)^ Using ‐5.1 eV for Fc/Fc^+^ against vacuum;

^j)^ Optical band gaps from the absorption onsets are larger by 0.2–0.3 eV than the HOMO‐LUMO gaps from CV.

NIR absorptions suggest that both donor substituents at the benzidole moiety enlarge the π‐conjugated system and that the conjugation for diphenylamine is stronger than for carbazole. Experimental absorption trends are confirmed by quantum chemical calculations of the electron density distribution of the frontier molecular orbitals (FMOs) and the resulting optical transitions. The highest occupied molecular orbital (HOMO) extends for all dyes over the whole π system (Figure 1b; Figures S4, S5, Supporting Information). For the diphenylamine substituents (**RSQ2** and **DCRSQ2**), the electron distribution extends to the phenyl groups, whereas the conjugation with the carbazole substituent is weak (**RSQ3** and **DCRSQ3**). The lowest unoccupied molecular orbitals (LUMOs) are concentrated along the polymethine bridges, and neither the oxygen nor the acceptor units show a notable contribution.

The trends of the calculated S1 HOMO → LUMO wavelengths and oscillator strengths (Table 1) agree with the experiment, with the calculated transition energy being larger by ≈0.3 eV for all dyes (Figure S6, Supporting Information). This constant energy offset allowed us to estimate the expected NIR absorption for benzindole‐substituted SQs containing alternative strong acceptors, such as the dicyanoindanone or difluoro‐dicyanoindanone moiety.^[^ ^10^, ^14^ ^]^ The calculated absorption maxima for these dyes were ≈900 nm, much below the experimental absorption of the corresponding rhodanine‐substituted dye **RSQ1** (1072 nm). This suggests that the rhodanine and dicyano‐rhodanine units are indeed among the strongest conceivable acceptors for the design of NIR SQs.

The experimental absorptions in the range ≈ 600 to 700 nm are assigned to HOMO‐1 → LUMO transitions, with HOMO‐2 contributing to **RSQ2** and **RSQ3** (Table 1). Most likely, the absorption bands at 510 nm originate from HOMO → LUMO+1 transitions. In the LUMO+1 MOs, the electron density extends to the donor units and remains weak on the oxygen but is notable on the acceptors. The calculated S3 HOMO → LUMO+1 transition wavelengths for **RSQ2** and **DCRSQ2** are the same when changing the solvent from toluene to dichloromethane, whereas a blue shift in absorption is calculated for the main NIR bands (Table 1). This agrees with the experiment, where the absorption at 510 nm is constant in the two solvents, but the NIR bands blueshift by 47 nm (for **RSQ2**) and 36 nm (for **DCRSQ2**) (Figure S3, Supporting Information).

Due to the steric demand of the acceptors, heterocycles on opposite sides of the polymethine chain are arranged in a *cis* configuration.^[^ ^36^ ^]^ The HOMO‐1 → LUMO and HOMO → LUMO+1 transitions are prominent for our dye series. These transitions in the visible are symmetry‐forbidden in parent SQs that exist in a *trans* configuration where the heterocycles are attached on opposite sides of the polymethine chain (C_2h_ symmetry), and are much weaker in *cis*‐locked symmetrical SQ dyes (C*_2v_* symmetry), as for example present with the dicyanomethylene acceptor.^[^ ^25^, ^37^ ^]^

**Figure 2:**
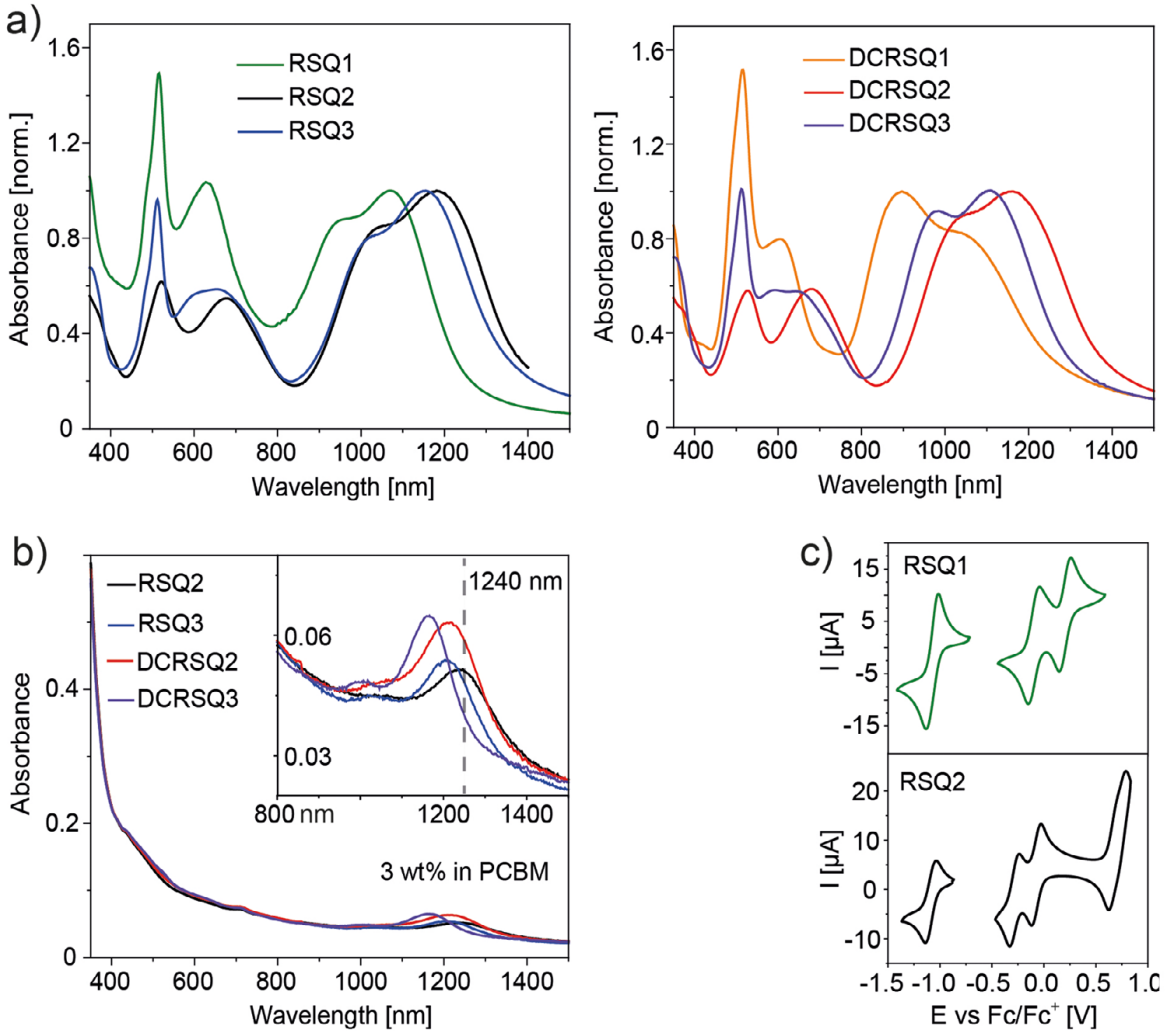
a) Film absorbance spectra of SQ dyes, coated from chloroform solution. b) Absorbance spectra of PCBM:SQ (3 wt%) host‐guest films. c) Cyclic voltammograms of **RSQ1** and **RSQ2**.

The NIR band shapes broaden in the film and an H‐dimer/aggregate peak covers the vibrational band observed in solution (**Figure** **2**a).^[^ ^23^ ^]^ This feature is noticeable for **DCRSQ1**. However, molecular ordering in pure SQ films remains minor, as the absorbance wavelength maxima of spin‐coated films and of solutions are quite similar (Table 1). Aggregation is completely suppressed when dispersing dyes in a [6,6]‐phenyl‐C_61_‐butyric acid methyl ester (PCBM) host matrix (Figure 2b), which was used for the fabrication of OPDs (see below). Absorptions shift bathochromically in the blend film, and the peak absorption of **RSQ2** is at 1240 nm.

Absorption wavelength trends and calculated FMO electron density distributions are reflected in the HOMO and LUMO levels measured via cyclic voltammetry (CV). All dyes show two reversible one‐electron oxidations and one reversible one‐electron reduction (Figure S7, Supporting Information). For the diphenylamine‐ and carbazole‐containing dyes, oxidation of the two donor groups results in an additional reversible two‐electron oxidation (Figure 2c). The strong diphenylamine donor resulted in an increase in the CV HOMO energy levels for **RSQ2** and **DCRSQ2** (Table 1). Variation of the CV LUMO energy levels was small, in agreement with the almost constant calculated electron distribution in the dye LUMOs. Taken together, increasing the elongation of the π‐system at the benzindole donors resulted in a narrowing of the electrochemical gap in each dye series.

### 2.2 Photodetector Characterization/Performance

**Figure 3:**
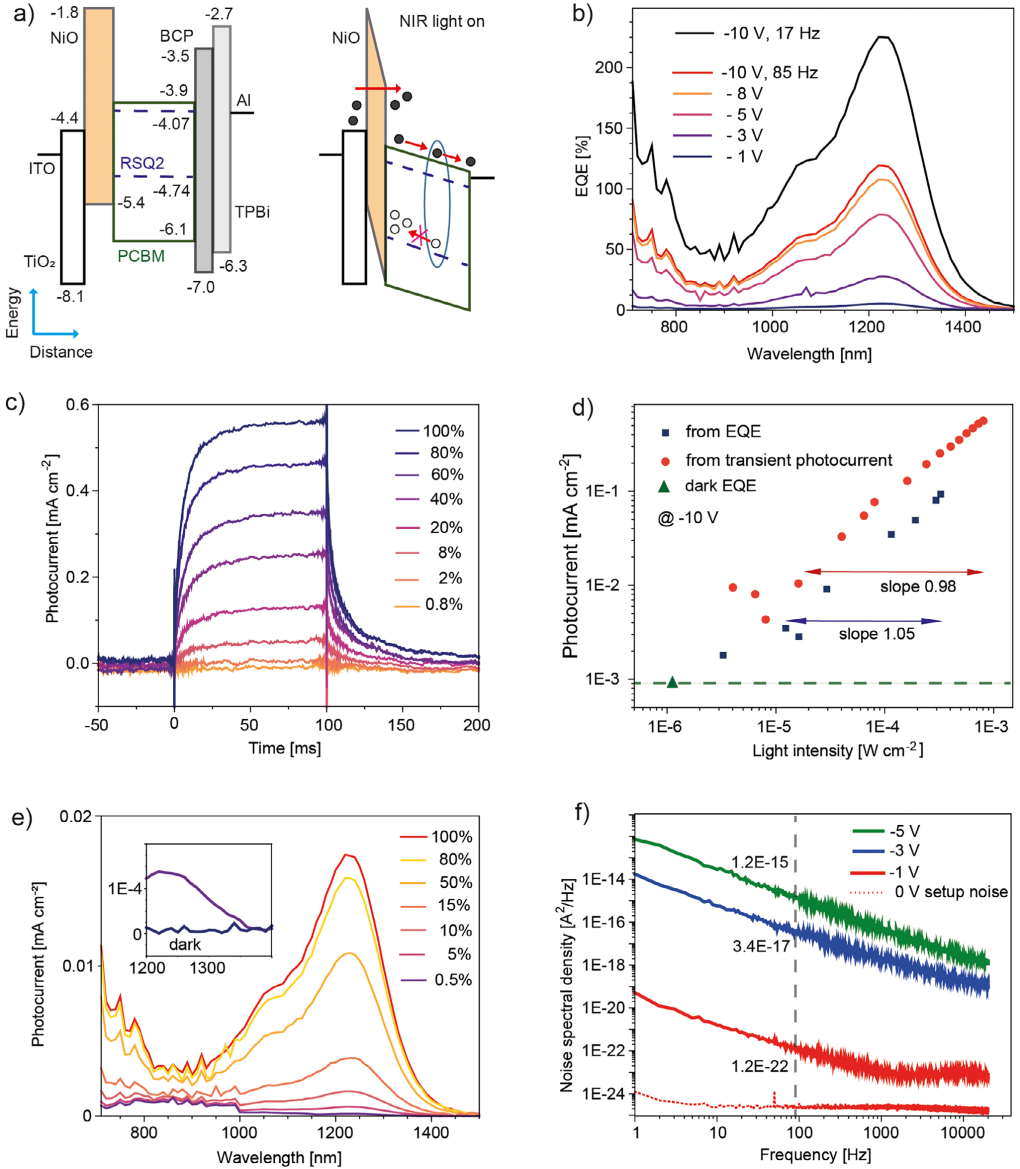
a) OPD stack with indicated energy values [eV] for the frontier orbitals, ITO/TiO_2_ 35 nm/NiO 5 nm/PCBM:**RSQ2** (3 wt%) 100 nm/BCP 5 nm/TPBi 4 nm/Al 60 nm, and device functionality in the presence of light. BCP is 2,9‐dimethyl‐4,7‐diphenyl‐1,10‐phenanthroline, TPBi is 1,3,5‐tris(1‐phenyl‐1H‐benzimidazol‐2‐yl)benzene, black circles denote electrons, empty circles denote holes. b) EQE spectra at different reverse bias, the light was modulated at 85 Hz. Included is the EQE spectrum at −10 V using a chopper rotation rate of 17 Hz. c) Photocurrent response at −10 V using light pulses at 1200 nm. d) OPD dynamic range from transient photocurrent (red circles) and EQE (blue squares) measurements. e) EQE spectra at different light intensities, at −1 V and 85 Hz. f) Noise spectral density as a function of frequency, with noise at 85 Hz indicated.

We used the most red‐shifted **RSQ2** dye for the design and optimization of NIR photodetectors, the basic device functionality using other SQs is shown in Figure S8 (Supporting Information). In the OPD stack (**Figure** **3**a, left), TiO_2_ serves to block any unintended hole extraction at the ITO anode, and BCP was used as an electron‐transporting/hole‐blocking layer^[^ ^9^, ^41^ ^]^ at the cathode (TPBi/Al).^[^ ^50^ ^]^ NiO acts as an electron‐blocking layer in the dark. In the presence of NIR light (Figure 3a, right), photogenerated electrons are transported via the LUMO of PCBM and are extracted at the cathode to the external circuit, while photogenerated holes are immobile and trapped in the active layer. Holes close to the anode induce an energy level bending over NiO, which results in a thin triangular energy barrier^[^ ^46^ ^]^ through which the injection of secondary electrons occurs. If the lifetime of trapped holes is longer than the transit time for electrons to sweep across the device, many extra electrons can traverse the device in addition to the photogenerated charge, resulting in photomultiplication.

The EQE increased with the applied bias and reached 120% at 1240 nm and at −10 V, indicating photomultiplication gain^[^ ^3^ ^]^ (Figure 3b). The corresponding device responsivity, the ratio between output photocurrent and input light power, is R = EQE x λ / 1240 = 1.2 A W^−1^. Photomultiplication is inferred from the faint light absorption in the NIR (Figure 2b; Figure S9, Supporting Information). The optical density at 1240 nm for the film containing 3 wt% **RSQ2** is only 0.017, which would limit the EQE to below 10% in a conventional OPD. EQE values were lower when decreasing (to 1 wt%) or increasing (to 5 wt%) the **RSQ2** concentration in the PCBM host (Figure S9, Supporting Information). This is because the optimum dye content is a tradeoff between weak light absorption and consequential small charge generation at low concentration, and the formation of percolating paths for holes at high dye loading, resulting in charge extraction rather than charge trapping.

Photomultiplication was dependent on the light modulation frequency used for the EQE measurement, and the EQE increased to 220% when reducing the chopper rotation rate from 85 Hz to 17 Hz (Figure 3b), and to ≈250% at 1 Hz (Figure S10, Supporting Information). This behavior is attributed to the presence of hole traps in the active layer. For photomultiplication OPDs, traps in the active layer often result in a long charge‐trapping time needed for significant energy level bending and subsequent charge‐detrapping and recombination, which inevitably results in a slow device response.^[^ ^9^, ^42^, ^45^ ^]^ The slow device response is confirmed by the transient photoresponse with current rise and fall times in the range of ≈ 20 ms (Figure 3c). This is much slower than the response speed of not trap‐limited NIR OPDs, for which typical cut‐off frequencies are well above 100 kHz, corresponding to current rise and fall times of a few microseconds.^[^ ^7^, ^35^, ^51^ ^]^

We evaluated the OPD dynamic range at ‐10 V from light‐dependent transient photocurrent and EQE measurements (Figure S11, Supporting Information). The dynamic range of a PD is the optical input range where the photoresponse can be accurately predicted.^[^ ^4^ ^]^ On the double logarithmic plot, the transient photocurrent versus light intensity (*I*) shows a near‐unity slope (Figure 3d, red circles), which indicates a linear dependence. However, the linear dynamic range (LDR = 20 x log[*I*_max_/*I*_min_] = 34) is moderate, limited by the available lamp intensity at the higher end and the dark current at the lower end of light intensity, which was 0.8 mA cm^−2^ at −10 V (Figure S12, Supporting Information). A linear relationship between photocurrent and light intensity means that within this range the processes of charge and trap generation in the PCBM: dye film, external charge injection through the NiO layer, and charge recombination in the organic layer are independent of the light intensity.

Light modulation in the EQE measurement suppressed the dark current (Figure 3d, green triangle), and the LDR = 28 extended to lower light intensity (blue squares). Again, the LDR is limited at the higher end of light by the available lamp intensity. The LDR offset between the EQE and transient photocurrent data is due to experimental unknowns when measuring the LED light intensity at 1200 nm (Supporting Information).

To estimate the sensitivity of our OPD, we evaluated the specific detectivity in the voltage range −1 to −5 V, the maximum allowed bias of our noise measurement setup. D^*^ = A^½^ x R / S_n_, where A (0.071 cm^2^) is the device area and S_n_ is the noise spectral density measured in the dark. D^*^ can be thought of as the signal‐to‐noise ratio generated by a PD with an area of 1 cm^2^ at 1 W incident light power, if the detection bandwidth is 1 Hz. Lowering the operation voltage from −10 to −1 V (Figure 3e) reduced the EQE dark current by two orders of magnitude and the LDR from light‐dependent EQE measurements increased to 66 (Figure S13, Supporting Information). The noise shows a dominant contribution from flicker noise (1/frequency noise), which is generally attributed to the trapping and detrapping of charge carriers (Figure 3f).^[^ ^4^, ^52^ ^]^ In our case, traps consist of oxidized squaraine dye molecules, but other sources of traps (interface traps, impurities) cannot be excluded at the moment. Flicker noise increased and extended to a higher frequency at a larger bias. This aligns with the strongly increasing dark current under a larger bias, where the number of stochastic processes of trapping and release increases because of the larger number of carriers being transported.

The noise spectral density was evaluated at 85 Hz, the frequency at which the responsivity was measured. At −1 V, the responsivity at 1240 nm was R = 0.06 A W^−1^ and the noise current S_n_ = 1.1 × 10^−11^ A Hz^½^, which results in D^*^ = 10^9^ Jones. With increasing voltage bias, D^*^ decreased steadily to D^*^ ≈ 5×10^6^ Jones at −5 V. This is because at higher bias the responsivity increase due to photomultiplication (Figure 3b) is overcompensated by a stronger increase of the noise current (Figure 3f). In general, the specific detectivity of photomultiplication OPDs is considerably smaller than for OPDs without gain.^[^ ^5^, ^43^ ^]^ For conventional NIR OPDs, values for D^*^ in the order of ≈ 10^13^ Jones have been reported.^[^ ^10^, ^17^ ^]^ One reason for this behavior is that triggering photomultiplication in a gain OPD often requires a high bias that results in a high dark current, especially problematic for NIR OPDs because the dark/noise current increases at longer wavelengths.^[^ ^4^, ^19^, ^20^ ^]^

We mention that the chosen thin, but still practicable, thickness for NiO (5 nm) and the active layer (100 nm) resulted in OPDs with the highest EQE. When increasing the NiO thickness to 10 nm, the photoresponse current decreased by one order of magnitude (Figure S14, Supporting Information). We also increased the active layer thickness from 100 to 200 nm (Figure S15, Supporting Information). When comparing the thin with the thick device at the same applied electric field, the EQE dropped by a factor of about 5, and the dark current in the thick device was lowered by ≈ 50 times (at E = 4 × 10^5^ V cm^−1^). The sensitivity of the OPD to weak optical signals depends on the signal‐to‐noise ratio; therefore, device parameters optimized for a high photoresponse do not necessarily also result in the highest detectivity. However, in our case, the OPD detectivity does not alter strongly, despite a stronger reduction of the dark current compared to the photocurrent with increasing active layer thickness. This can be illustrated by evaluating the shot‐noise limited detectivity for the two devices, D^*^_sh_ = R / (2 x q x J_d_)^½^, where q is the value of the electron charge and J_d_ [A cm^−2^] the dark current density. D^*^_sh_ represents an upper bound of the true specific detectivity and assumes that under reverse bias the shot noise from the dark current is the dominant contribution to the overall noise. In our device, the shot‐noise current dominates over the thermal noise current (I_th_)^2^ = (4 x k_b_ x T) / R_sh_ (Supporting Information). D^*^_sh_ was (1 ± 0.4) x 10^11^ Jones for both the thin and thick device, independent of the bias.

## 3 Conclusion

Exploring the chemical combination of strong electron‐donating with –accepting groups in SQs, we were able to synthesize a set of novel NIR SQ dyes with maximum absorption above 1100 nm in solution, extending to beyond 1400 nm in the film. Among commonly known electron‐acceptor groups, the rhodanine‐substituted squaric acid core induces the strongest red‐shift in absorption. Further extending the π‐conjugation on the donor group might shift the absorption to a longer wavelength; however, for any real‐world use, the synthetic accessibility and important material properties of the corresponding SQ dye, including thermal stability and solubility, should be ensured. Alternatively, long wavelength absorption can be attempted via SQ dye aggregation in the film, although it seems that the predictable supramolecular engineering of SQs to form J‐aggregates is more difficult than for the related polymethine cyanine dyes. As an example of use for NIR SQs, we developed fullerene‐host: SQ‐guest NIR‐sensitive OPDs with photomultiplication. The OPD achieves an EQE of over 200% at 1240 nm. A high responsivity is the first essential requirement for the realization of efficient OPDs beyond the silicon cutoff. Therefore, to increase the specific detectivity of our photomultiplication NIR OPD design, device optimization efforts should be directed to contain the dark/noise current, because the signal component of the signal‐to‐noise consideration is already high.

## Conflict of Interest

The authors declare no conflict of interest.

## Supporting information



Supporting Information

## Data Availability

The data that support the findings of this study are available in the supplementary material of this article.
