# Supplementary material for: Squaraine Dyes for Organic Photomultiplication Photodetectors with 220% External Quantum Efficiency at 1240 nm
Source: Adv Sci (Weinh). 2025 Apr 3;12(26):2502320. doi: 10.1002/advs.202502320 (PMC12245026; doi:10.1002/advs.202502320)
Supplement: Supplementary file 1 — Supporting Information [file ADVS-12-2502320-s001.docx]

Supporting Information

**Squaraine Dyes for Organic Photomultiplication Photodetectors with 220% External Quantum Efficiency at 1240 nm**

Joshua Csucker, Elodie Didier, João Pedro Ferreira Assunção, Daniel Rentsch, Radha Kothandaraman, Dominik Bachmann, Ivan Shorubalko, Frank Nüesch, Roland Hany^*^, Michael Bauer

* roland.hany@empa.ch

Table of Contents

[1. General Procedures 1](#_Toc184799209)

[2. Synthetic Procedures 4](#_Toc184799210)

[3. TGA Data 16](#_Toc184799211)

[4. Optical Spectroscopy Data and Calculations 17](#_Toc184799212)

[5. Cyclic Voltammetry Data 21](#_Toc184799213)

[6. Photodetector Data 22](#_Toc184799214)

[7. NMR Spectra 28](#_Toc184799215)

[8. MS Spectra 45](#_Toc184799216)

# General Procedures

Chemicals and solvents were purchased from Merck, TCI, Fluorochem and VWR and were used without further purification. Column chromatography was done using silica gel (pore size 40-63 μm, Normasil 60® from VWR).

^1^H, ^13^C and ^31^P NMR data were recorded at 400.2 MHz, 100.6 MHz and 162.0 MHz using a 5 mm CryoProbe™ Prodigy probe equipped with z-gradient on a Bruker Avance III 400 NMR spectrometer (Bruker Biospin AG, Fällanden, Switzerland). 1D and 2D NMR experiments were performed at 298 K (unless otherwise stated) using the Bruker standard pulse programs and parameter sets applying 90° pulse lengths of 11.4 µs (^1^H), 10.0 µs (^13^C) and 10.7 μs (^31^P). Chemical shifts (δ in ppm) were calibrated to residual solvent peaks of THF-d_8_ (δ = 1.72 and 25.2 ppm) and CDCl_3_ (δ = 7.26 and 77.16 ppm). For ^31^P the chemical shift (δ = 0 ppm) was referenced against an external sample of H_3_PO_4_. Coupling constants *J* are reported in Hz and patterns are described as s = singlet, d = doublet, t = triplet, q = quartet, quint = quintet, sept = septet, m = multiplet, br = broad and for ^13^C NMR data s = quaternary carbon, d = CH, t = CH_2_, and q = CH_3_. NMR spectra of **RSQ3** were recorded at 258 K, as a broadening of resonances was observed at higher temperatures (up to 333 K), particularly in the chemical shift region of aromatic protons.

High-resolution electrospray ionization mass spectra (HR-ESI-MS) were acquired on a Bruker Daltonics maXis ESI-QTOF at ETH Zürich. The mass spectrometer was operated in the positive electrospray ionization mode at 4500 V capillary voltage and -500 V endplate offset. A nebulizer pressure of 1.6 bar and a dry gas flow of 7 L min^-1^ at 230 °C were used. Mass spectra were acquired in a mass range from m/z 100 to 5000 and the mass analyzer was calibrated between m/z 118 and 2721 using a tunemix solution.

Thermogravimetric analysis was performed on a Netzsch TG 209 F1 Iris with Mettler-24123 aluminium oxide crucibles at a heating rate of 20 °C min^-1^ under nitrogen atmosphere.

Cyclic voltammetry was measured on a computer-controlled μAutolab Type III potentiostat^[S1]^ using a three electrode system with a GC working electrode, a Pt counter electrode and an Ag/AgCl pseudo reference electrode in dichloromethane with 0.1 M of (*n*Bu)_4_NPF_6_ as supporting electrolyte. The scan rate was 100 mV s^-1^. The potentials were internally referenced to the ferrocene/ferrocenium (Fc/Fc^+^) redox pair assuming a HOMO energy level of -5.1 eV vs. vacuum.^[S2]^ Half-wave potentials E_1/2_ were determined with the help of the cathodic (E_pc_) and anodic (E_pa_) peak potentials with E_1/2_=1/2(E_pc_+E_pa_). HOMO and LUMO energies were determined with the onset of the oxidation and reduction, respectively, with E_HOMO_ = (-5.1 eV - E_ox,ons_) and E_LUMO_ = (-5.1 eV – E_red,ons_).

Absorption spectra of solutions and thin films were recorded on a Perkin Elmer Lambda 950 or a Shimadzu UV-VIS3600 spectrophotometer. A cuvette filled with solvent or the glass substrate was used as the baseline. To obtain the extinction coefficient a stock solution was diluted to different concentrations. Extinction coefficients were calculated by plotting absorbance versus concentration and using a linear fit.

Quantum chemical calculations were carried out using Orca 5.^[S3,S4]^ Geometry optimization was done using the B3LYP hybrid functional with a def2-SVP base set utilizing the resolution of identity approximation.^[S5,S6]^ Excited states were calculated using the M062X functional with a def2-TZVP functional and the CPCM solvation model with toluene as the solvent.^[S7,S8]^ Results were visualized with Avogadro, Version 1.20.^[S9]^

For the fabrication of OPDs, TiO_2_ (35 nm) was coated on cleaned ITO substrates via a sol-gel process.^[S10]^ NiO layers (5, 7 and 10 nm) were deposited by RF sputtering (CT200, Alliance concept) using a ceramic NiO target at r.t., with an Ar gas flow of 100 standard cubic centimeter per minutes at a power of 250 W. For the active layer, precursor solutions were prepared by dissolving individually PCBM and the squaraine dye in anhydrous chloroform (17.5 mg mL^-1^) and solutions were then stirred for 30 min in a glovebox. Dyes were added to the PCBM solution in amounts of 1, 3 and 5 wt-%. PCBM:dye films were spin-coated from the filtered (0.45 µm, hydrophilic PTFE) solutions at 3000 rpm (30 s) for a 100 nm thick film, and at 1000 rpm (20 s) for a 200 nm thick film. Bathocuproine (BCP, 5 nm, Merck) was spin-coated from a 4 mg mL^-1^ anhydrous isopropanol solution at 4000 rpm for 60 s. 1,3,5-Tris(1-phenyl-1*H*-benzimidazol-2-yl)benzene (TPBi, Lumtec, 4 nm, 0.1 Å s^-1^) was thermally evaporated on the whole substrate at a pressure below 5x10^-6^ mbar. Al (Kurt J. Lesker) was thermally evaporated (0.2-0.5 Å s^-1^, 70 nm) through a shadow mask defining eight cells with area 3.1 and 7.1 mm^2^ per substrate. Energy levels indicated in Fig. 3a (main text) were taken from the literature: TiO_2_,^[S11]^ NiO,^[S12]^ PCBM,^[S13]^ BCP,^[S14]^ , TPBi.^[S15]^

Current-Voltage curves and the device response were measured inside a glovebox using the Paios measurement system (Fluxim AG, Switzerland). Varying light intensities were applied using LEDs at 950 nm (maximum 147.5 W m^-2^) and 1200 nm (maximum ≈8.06 W m^-2^). The indicated light intensity at 1200 nm is approximate to some extent because of the used LED (MTSM0012-843-IR from Marktech Optoelectronics), for which the spectral width (≈100 nm) and the peak wavelength (from 1150 to 1250 nm) can vary from batch to batch. In addition, it was not possible to precisely match the distance between the lamp and the sample, and between the lamp and the Ge photodiode used for calibration, respectively. EQE spectra were measured on a commercial setup (SpeQuest, ReRa solutions BV) using chopped monochromatic light with an optional voltage bias (maximum allowed -10 V) applied. The maximum EQE light intensity at 1240 nm was 3.26 W m^-2^ and was calibrated with a Ge standard photodiode (S122C, Thorlabs).

The noise spectral density was measured in the dark using a battery-powered SR570 JV-Amplifier (Stanford Research) that allows a maximum voltage of -5 V to be applied. The device current was amplified with the same amplifier and, subsequently, time traces (100 s) of the amplifier's output were recorded using a USB-6281 data acquisition system (National Instruments). Recorded time traces were split in segments of 1 s and for each segment the DC component was removed, the noise power spectral density was evaluated and subsequently data were averaged. The device was disconnected for measuring the instrumental noise of the setup.

The noise spectral density displayed in Figure 3f (main text) accounts for all noise components, including the frequency-dependent 1/f noise as well as the frequency-independent thermal and shot noise. The thermal noise current was (I_thermal_)^2^ = 4 x k_b_ x T / R_sh_ = 2 x 10^-25^ A^2^ Hz^-1^. (I_thermal_)^2^ was close to the shot noise current (I_shot_)^2^ = 2 x q x I_d_ = 5 x 10^-25^ A^2^ Hz^-1^ at -1 V, but (I_shot_)^2^ dominated over the thermal noise current at -3 V (= 1.4 x 10^-24^ A^2^ Hz^-1^) and -5 V (= 3.2 x 10^-24^ A^2^ Hz^-1^).

# Synthetic Procedures

**1-Octylbenzo[*cd*]indol-2(1*H*)-one (1)**

A two-necked round bottom flask (100 mL) was charged with sulfolane (20 mL) and benzo[*cd*]indol-2(1*H*)-one (5.07 g, 30.0 mmol, 1.0 eq). Potassium iodide (KI, 0.99 g, 6.0 mmol, 0.2 eq) and 4-dimethylaminopyridine (DMAP, 0.51 g, 4.2 mmol, 0.14 eq) were added. A reflux condenser was equipped and the reaction mixture was heated to 70 °C. N-bromooctane (7.77 mL, 45 mmol, 1.5 eq) and potassium hydroxide (KOH, 3.03 g, 53.9 mmol, 1.8 eq) were added in three portions at 1 h intervals. Reaction monitoring by TLC indicated full consumption of the starting material after 3.25 h after which the reaction mixture was cooled to r.t.. The crude mixture was diluted with ethyl acetate (EtOAc, 400 mL) and washed with H_2_O (60 mL). The aqueous (aq.) phase was extracted with EtOAc (3x20 mL), the combined organic fractions were dried over sodium sulfate (Na_2_SO_4_) and the solvent removed *in vacuo*. The lactam was purified by flash column chromatography (SiO_2_, hexane/dichloromethane (CH_2_Cl_2_), 1:1) and the final product was dried under high vacuum. The alkylated lactam **1** (8.4 g, 29.9 mmol, quant.) was obtained as yellow oil. The reaction was adapted from a previously published procedure.^[S10]^

**R*_f_*** (SiO_2_, hexane/CH_2_Cl_2_, 1:1): 0.25.

**^1^H NMR** (400.2 MHz, CDCl_3_): δ = 8.05 (d, *J* = 7.0, 1H); 8.00 (d, *J* = 8.1, 1H); 7.70 (dd, *J* = 8.1, 7.0, 1H); 7.52 (d, *J* = 8.4, 1H); 7.46 (dd, *J* = 8.4, 6.9, 1H); 6.90 (d, *J* = 6.9, 1H); 3.91 (t, *J* = 7.3, 2H); 1.78 (m, 2H); 1.50 – 1.26 (m, 10H); 0.86 (t, *J* = 7.0, 3H).

**6-Bromo-1-octylbenzo[*cd*]indol-2(1*H*)-one (2)**

A three-necked round bottom flask (250 mL) was equipped with a stir bar, a graduated dropping funnel (50 mL), an argon inlet and a septum. 1-Octylbenzo[*cd*]indol-2(1*H*)-one **1** (8.4 g, 29.9 mmol, 1.0 eq) was dissolved in CH_2_Cl_2_ (100 mL). The yellow solution was cooled to -5 °C with a water ice bath to which sodium chloride (NaCl) was added. N-bromosuccinimide (NBS, 4.9 g, 27.5 mmol, 0.92 eq) was dissolved in acetonitrile (CH_3_CN, 50 mL). The reactant solution was added dropwise to the reaction mixture via the graduated dropping funnel over the course of 1.5 h. After addition was complete, the yellow solution was stirred over night for a total reaction time of 18 h. During this time, the temperature gradually increased to r.t.. The solvent was removed *in vacuo*. The crude solids were dissolved in EtOAc (100 mL) and washed with sat. aq. sodium bicarbonate (NaHCO_3_, 2x100 mL). The organic layer was dried over Na_2_SO_4_ and the solvent removed *in vacuo*. The aryl bromide **2** was purified by flash column chromatography (SiO_2_, hexane/CH_2_Cl_2_, 1:2), dried under high vacuum and **2** was obtained as a yellow solid (8.8 g, 24.4 mmol, 89% based on added NBS).

**R*_f_*** (SiO_2_, hexane/CH_2_Cl_2_, 1:2): 0.23.

**^1^H NMR** (400.2 MHz, CDCl_3_): δ = 8.14 (d, *J* = 8.3, 1H); 8.08 (d, *J* = 7.0, 1H); 7.79 (dd, *J* = 8.3, 7.0, 1H); 7.65 (d, *J* = 7.5, 1H); 6.76 (d, *J* = 7.5, 1H); 3.88 (t, *J* = 7.3, 2H); 1.76 (m, 2H); 1.5 – 1.2 (m, 10H); 0.86 (t, *J* = 7.1, 3H). Data agree with reported values.^[S1]^

**6-(Bis(4-methoxyphenyl)amino)-1-octylbenzo[*cd*]indol-2(1*H*)-one (3)**

A Schlenk round bottom flask (250 mL) was equipped with a stir bar. Tris(dibenzylideneacetone)dipalladium(0) (Pd_2_(dba)_3_, 0.5 g, 0.55 mmol, 0.05 eq) and tri-*tert*-butylphosphonium tetrafluoroborate (P(tBu)_3_HBF_4_, 0.16 g, 0.55 mmol, 0.05 eq) were added to the flask in a glovebox. Degassed dry toluene (40 mL) was added to the Schlenk flask resulting in a deep red solution. Premixed **2** (4.0 g, 11.1 mmol, 1.0 eq), bis(4-methoxyphenyl)amine (3.1 g, 13.3 mmol, 1.2 eq) and potassium *tert*-butoxide (KOtBu, 3.7 g, 33 mmol, 3 eq) were added to the reaction vessel. A reflux condenser was attached and the red reaction mixture was stirred at 110 °C for 16 h. The solvent was removed after cooling to r.t. *in vacuo*. The red sticky crude was dissolved in EtOAc (100 mL) and washed with H_2_O (2x200 mL). The aq. phase was filtered over Celite® to remove residual Pd. The aq. layer was extracted with EtOAc (3x100 mL). The combined organic fractions were dried over Na_2_SO_4_ and the solvent removed *in vacuo*. The aryl amine was purified by flash column chromatography (SiO_2_, manual gradient of 0% MeOH in CH_2_Cl_2_ to 5% MeOH in CH_2_Cl_2_) and the final product was dried on high vacuum. Aryl amine **3** (4.8 g, 9.44 mmol, 85%) was obtained as a sticky red solid.

**R*_f_*** (SiO_2_, CH_2_Cl_2_): 0.17.

**^1^H NMR** (400.2 MHz, CDCl_3_): δ = 7.99 (d, *J* = 6.9, 1H); 7.79 (d, *J* = 8.2, 1H); 7.49 (dd, *J* = 8.2, 6.9, 1H); 7.06 (d, *J* = 7.6, 1H); 6.98 (m, 4H); 6.78 (m, 5H); 3.89 (t, *J* = 7.2, 2H); 3.77 (s, 6H); 1.79 (m, 2H); 1.5 – 1.2 (m, 10H); 0.88 (t, *J* = 6.9, 3H). **^13^C NMR** (100.6 MHz, CDCl_3_): δ = 168.1 (s); 155.0 (s, 2C); 142.9 (s, 2C); 140.2 (s); 136.3 (s); 129.0 (d); 128.2 (d); 127.4 (s); 127.1 (s); 126.6 (s); 125.8 (d); 124.3 (d); 124.2 (d, 4C); 114.7 (d, 4C); 106.1 (d); 55.6 (q, 2C); 40.4 (t); 31.9 (t); 29.4 (t); 29.3 (t); 29.0 (t); 27.1 (t); 22.7 (t); 14.2 (q).

**6-(Bis(4-methoxyphenyl)amino)-2-methyl-1-octylbenzo[*cd*]indol-1-ium hexafluorophosphate (4)**

A three necked round bottom flask was equipped with a low temperature thermometer, an argon inlet and a septum. Dry tetrahydrofurane (THF, 15 mL) and lactam **3** (1.00 g, 1.97 mmol, 1.0 eq) was added to the reaction vessel and the red solution was cooled to 0 °C. Methyl magnesium chloride (MeMgCl, 1.3 mL, 3 M in THF, 3.90 mmol, 2 eq) was added dropwise via syringe. After stirring the red solution at 0 °C for 30 min, the solution was heated to 60 °C for 2.5 h. Reaction control was performed by quenching an aliquot with HCl which turned the analyte solution deep blue, followed by TLC. Upon complete consumption of lactam **3**, the crude reaction mixture was cooled to r.t. and added to cold aq. HCl (0.5 M, 10 mL). The blue solution was concentrated *in vacuo* to a third of the original volume and potassium hexafluorophosphate (KPF_6_, 0.7 g, 3.80 mmol, 2 eq) was added. The aq. layer was extracted with CH_2_Cl_2_ (2x10 mL). The combined org. fractions were washed with H_2_O (2x20 mL). The solvent was removed *in vacuo*. The iminium salt **4** (1.3 g, 1.97 mmol) was used in subsequent steps without purification.

**^1^H NMR** (400.2 MHz, CDCl_3_): δ = 8.39 (d, *J* = 7.4, 1H); 7.78 (d, *J* = 8.2, 1H); 7.69 (d, *J* = 8.6, 1H); 7.53 (dd, *J* = 8.2, 7.4, 1H); 7.18 (m, 4H); 6.92 (m, 5H); 4.51 (t, *J* = 7.5, 2H); 3.85 (s, 6H); 3.03 (s, 3H); 1.94 (m, 2H); 1.5 – 1.2 (m, 10H); 0.86 (t, *J* = 6.9, 3H). **^13^C NMR** (100.6 MHz, CDCl_3_): δ = 160.0 (s); 158.3 (s, 2C); 153.0 (s); 140.7 (s, 2C); 136.5 (d); 133.4 (d); 130.3 (s); 128.7 (s); 128.3 (d); 127.4 (d, 4C); 124.5 (s); 123.8 (s); 123.2 (d); 119.3 (d); 115.9 (d, 4C); 55.7 (q, 2C); 47.0 (t); 31.8 (t); 30.8 (t); 29.2 (t); 29.1 (t); 26.9 t); 22.7 (t); 14.2 (q); 12.9 (q). **^31^P NMR** (162.0 MHz, CDCl_3_): δ = -144.3 (sept, *J* = 713, PF_6_).

**HR-ESI-MS**: [M]^+^ C_34_H_39_N_2_O_2_; calc 507.3006 m/z; found 507.2999 m/z (1.4 Δppm).

**RSQ1** the synthesis of **RSQ1** is reported in reference [S16].

**(3E,4Z)-4-((6-(bis(4-methoxyphenyl)amino)-1-octylbenzo[cd]indol-1-ium-2-yl)methylene)-2-((E)-(6-(bis(4-methoxyphenyl)amino)-1-octylbenzo[cd]indol-2(1H)-ylidene)methyl)-3-(3-ethyl-4-oxo-2-thioxothiazolidin-5-ylidene)cyclobut-1-en-1-olate (RSQ2)**

A three-necked round bottom flask was equipped with a septum, reflux condenser and an argon inlet. The reaction vessel was charged with toluene (20 mL), n-butanol (*n*BuOH, 20 mL) and **4** (515 mg, 0.79 mmol, 2 eq). The blue solution was stirred at 60 °C for 10 min. Triethylammonium 2-butoxy-3-(3-ethyl-4-oxo-2-thioxothiazolidin-5-ylidene)-4-oxo-cyclobut-1-en-1-olate **9**^[S16]^ (2 mL, 0.20 M (in PhMe), 0.39 mmol, 1.0 eq) was added via syringe. The reaction mixture turned green immediately. The reaction mixture was stirred at 60 °C for 6 h. The solvent was removed *in vacuo*. The dark crude material was purified by SiO_2_ flash column chromatography (flush with 5:1 hexane/EtOAc followed by collection of the **RSQ2** by flushing with CH_2_Cl_2_), followed by solvent evaporation. Resulting black solids were suspended in heptane and filtered. The filter cake was collected and purified by automated flash column chromatography (SiO_2_, linear gradient of 1:1 heptane/CH_2_Cl_2_ to 100% CH_2_Cl_2_ over 15 min, 12 g cartridge, 30 mL min^-1^). Fractions containing the title compound were combined and the solvent was removed *in vacuo*. The purple crude solids were dissolved in minimal amounts of CH_2_Cl_2_ and precipitated by addition of heptane. **RSQ2** (88 mg, 71 μmol, 18%) was obtained as dark solid.

**^1^H NMR** (400.2 MHz, d_8_-THF): δ = 8.77 (d, *J* = 7.5, 1H); 8.57 (s, 1H); 8.40 (d, *J* = 7.4, 1H); 7.69 (d, *J* = 8.3, 1H); 7.64 (d, *J* = 8.3, 1H); 7.54 (m, 2H); 7.23 (d, *J* = 8.0, 1H); 7.09 (d, *J* = 8.0, 1H); 7.0 (m, 10H); 6.8 (m, 8H); 6.33 (s, 1H); 4.36 (t, *J* = 7.6, 2H); 4.25(t, *J* = 7.3, 2H); 4.19 (q, *J* = 7.0, 2H); 3.73 (s, 6H); 3.73 (s, 6H); 2.0 (m, 4H); 1.6 (m, 4H); 1.5 – 1.2 (m, 19H); 0.9 (m, 6H). **^13^C NMR** (100.6 MHz, d_8_-THF): δ = 189.2 (s); 175.9 (s); 164.9 (s); 160.7 (s); 157.9 (s); 156.9 (s, 2C); 156.5(s, 2C); 150.9 (s); 149.1 (s); 144.0 (s); 143.5 (s, 2C); 143.4 (s, 2C); 142.1 (s, 2C); 138.4 (s); 137.4 (s); 132.9 (d); 131.3 (s); 131.1 (s); 131.1 (d); 129.0 (d); 128.9 (d, 2C); 127.7 (d); 127.6 (s); 127.5 (s); 127.1 (s); 126.2 (d); 125.9 (d, 4C); 125.4 (d, 4C); 124.9 (d); 115.4 (d, 4C); 115.3 (d, 4C); 110.9 (d); 108.7 (d); 101.2 (d); 94.8 (d); 90.2 (s); 55.5 (q, 4C); 45.2 (t); 44.6 (t); 39.5 (t); 32.7 (t, 2C); 30.3 (t); 30.2 (t, 4C); 29.7 (t); 28.1 (t); 27.8 (t); 23.4 (t, 2C); 14.4 (q, 2C); 12.6 (q).

**Absorption** (CH_2_Cl_2_): λ_max_ = 1118 nm; λ_max_ = 659 nm; λ_max_ = 515 nm; **Absorption** (PhMe): λ_max_ = 1165 nm (80000 M^-1^cm^-1^); λ_max_ = 669 nm; λ_max_ = 510 nm.

**HR-ESI-MS**: [M+H]^+^ C_77_H_80_N_5_O_6_S_2_; calc 1234.5545 m/z; found 1234.5514 m/z (2.4 Δppm).

Attempts to crystallize **RSQ2** were not successful.

**6-(3,6-Dimethoxy-9*H*-carbazol-9-yl)-2-methyl-1-octylbenzo[cd]indol-1-ium hexafluorophosphate (6)**

A Schlenk flask (250 mL) was charged with [Pd_2_(dba)_3_] (666 mg, 727 μmol, 0.09 eq), P(tBu)_3_HBF_4_ (342 mg, 1.18 mmol, 0.14 eq), 3,6-dimethoxy-9*H*-carbazole (2.27 g, 10 mmol, 1.2 eq) and **2** (3.0 g, 8.33 mmol, 1.0 eq). The solids were degassed by high vacuum for 30 min. Argon-degassed toluene (150 mL) was added via cannula. Sodium *tert*-butylate (NaOtBu, 2.4 g, 25 mmol, 3.0 eq) was added and the red-brown solution was stirred at 110 °C for 16 h. The solvent was removed *in vacuo*. The crude material was flushed over a silica gel plug (6:1 hexane/EtOAc). Fractions containing the title compound (contaminated with 3,6-dimethoxy-9H-carbazole) were combined and the solvent removed *in vacuo*. The aryl amine **5** (3.3 g, 6.51 mmol, 78% crude yield) was isolated as an orange solid. A three-necked round bottom flask was equipped with a stir bar, reflux condenser, gas inlet and septum. The reaction vessel was charged with crude lactam **5** (265 mg, 523 μmol, 1.0 eq) and dry THF (10 mL). The resulting yellow solution was cooled to 0 °C and MeMgCl (3 M in THF, 0.4 mL, 1.20 mmol, 2.3 eq) was added to the reaction mixture. The temperature was maintained at 0 °C for 1 h and subsequently heated to 60 °C for 1.5 h. The pale yellow reaction mixture was cooled to r.t. upon complete consumption of the starting material. Addition of the crude material to an aq. solution of KPF_6_ (200 mg in 50 mL) caused a yellow precipitate to form. The pH was adjusted to 1 by addition of aq. HCl (1 M, 5 mL) which turned the suspension deep blue. The aq. phase was extracted into CH_2_Cl_2_ (2x10 mL) with 2 mL CH_3_CN to aid with phase separation. The combined organic fractions were washed with H_2_O (2x10 mL), dried with Na_2_SO_4_, and the solvent removed *in vacuo*. Iminium salt **6** (317 mg, 487 μmol) was obtained as a deep blue solid. The material was used in subsequent steps without further purification.

**^1^H NMR** (400.2 MHz, CDCl_3_): δ = 8.69 (d, *J* = 7.3, 1H); 8.34 (d, *J* = 8.3, 1H); 8.21 (d, *J* = 7.9, 1H); 7.91 (d, *J* = 7.9, 1H); 7.90 (dd, *J* = 8.3, 7,3, 1H); 7.57 (d, *J* = 2.5, 2H); 7.22 (d, *J* = 8.9, 2H); 7.00 (dd, *J* = 8.9, 2.5, 2H); 4.70 (t, *J* = 7.6, 2H); 3.96 (s, 6H); 3.25 (s, 3H); 2.07 (m, 2H); 1.55 (m, 2H); 1.5-1.2 (m, 8H); 0.87 (t, *J* = 6.9, 3H). **^13^C NMR** (100.6 MHz, CDCl_3_): δ = 171.0 (s); 155.4 (s, 2C); 140.8 (s); 137.1 (s, 2C); 137.0 (s); 136.5 (d); 135.9 (d); 131.0 (d); 130.0 (s); 128.1 (d); 126.5 (s); 125.0 (s, 2C); 124.7 (s); 122.0 (d); 115.7 (d, 2C); 110.8 (d, 2C); 103.4 (d2, 2C); 56.2 (q, 2C); 47.9 (t); 31.8 (t); 30.6 (t); 29.3 (t); 29.2 (t); 27.1 (t); 22.7 (t); 14.2 (q); 14.1 (q). **^31^P NMR** (162.0 MHz, CDCl_3_): δ = -144.5 (sept, *J* = 713).

**HR-ESI-MS**: [M]^+^ C_34_H_37_N_2_O_2_; calc 505.2850 m/z; found 505.2839 m/z (2.0 Δppm).

**(3Z,4Z)-4-((6-(3,6-dimethoxy-9H-carbazol-9-yl)-1-octylbenzo[cd]indol-1-ium-2-yl)methylene)-2-((E)-(6-(3,6-dimethoxy-9H-carbazol-9-yl)-1-octylbenzo[cd]indol-2(1H)-ylidene)methyl)-3-(3-ethyl-4-oxo-2-thioxothiazolidin-5-ylidene)cyclobut-1-en-1-olate (RSQ3)**

A Schlenk round bottom flask was equipped with a septum. The reaction vessel was charged with PhMe (5 mL), *n*BuOH (5 mL) and iminium salt **6** (174 mg, 267 μmol, 2.3 eq). The blue solution was stirred at 60 °C for 10 min. Triethylammonium 2-butoxy-3-(3-ethyl-4-oxo-2-thioxothiazolidin-5-ylidene)-4-oxo-cyclobut-1-en-1-olate **9** (0.24 mL, 0.49 M in PhMe, 0.12 mmol, 1.0 eq) was added via syringe. The reaction mixture turned dark immediately. The reaction mixture was stirred at 60 °C for 1.75 h. The reaction mixture was cooled to r.t. and diluted with CH_2_Cl_2_ (50 mL). The organic phase was washed with H_2_O (50 mL), dried over Na_2_SO_4_ and the solvent was removed *in vacuo*. The dark crude material was purified by SiO_2_ flash column chromatography (1:1 hexane/EtOAc) and the solvent was removed *in vacuo*. Resulting black solids were suspended in heptane and filtered. The filter cake was collected and purified by automated flash column chromatography (SiO_2_, linear gradient of 1:10 heptane/EtOAc to 1:1 heptane/EtOAc). Fractions containing **RSQ3** were combined and the solvent was removed *in vacuo*. The purple crude solids were dissolved in minimal amounts of CH_2_Cl_2_ and precipitated by addition of heptane. The title compound **RSQ3** (99 mg, 80 μmol, 68%) was obtained as dark blue solid.

Chemical structure of **RSQ3** with numbering of positions.

**^1^H NMR** (400.2 MHz, d_8_-THF), measured at 258 K: δ = 8.91 (d, *J* = 7.4, 1H, H-16b); 8.74 (s, 1H, H-27b); 8.51 (d, *J* = 7.4, 1H, H-16a); 7.75 (m, 1H, H-9b); 7.73 (m, 4H, H-3); 7.72 (m, 1H, H-15b); 7.71 (m, 1H, H-9a); 7.69 (m, 1H, H-15a); 7.63 (d, *J* = 7.8, 1H, H-10b); 7.52 (d, *J* = 8.3, 1H, H-14b); 7.50 (d, *J* = 8, 1H, H-10a); 7.43 (d, *J* = 8.2, 1H, H-14a); 7.09 (m, 4H, H-6); 6.92 (m, 4H, H-7); 6.46 (s, 1H, H-27a); 4.47 (t, *J* = 7.7, 2H, H-19b); 4.37 (t, *J* = 7.7, 2H, H-19a); 4.19 (q, *J* = 7.2, 2H, H-34); 3.88 (s, 12H, H-1); 2.05 (m, 2H, H-20b); 1.99 (m, 2H, H-20a); 1.64 (m, 2H, H-21b); 1.61 (m, 2H, H-21a); 1.50-1.30 (m, 8H, H-22a, 22b, 23a, 23b); 1.30 (m, 8H, H-24a, 24b, 25a, 25b); 1.27 (m, 3H, H-35); 0.88 (m, 6H, H-26a, 26b). **^13^C NMR** (100.6 MHz, d_8_-THF), measured at 258 K: δ = 189.3 (s, C-33); 175.2 (s, C-29); 167.1 (s, C-28b); 165.1 (s, C-32); 163.3 (s, C-28a); 157.3 (s, C-30); 155.4-155.3 (s, 4C, C-2); 152.3 (s, C-18b); 150.6 (s, C-18a); 141.7 (s, C-11a); 141.1 (s, C-11b); 138.2-138.1 (s, 4C, C-5); 133.9 (d, C-16b); 132.5 (d, C-16a); 131.7 (s, C-8b); 131.2 (s, C-17a); 131.1 (s, C-17b); 130.4 (s, C-8a); 130.4 (d, C-15b); 130.3 (d, C-15a); 129.2 (d, C-9a); 129.1 (d, C-9b); 128.0 (s, C-13a); 128.0 (d, C-14b); 127.8 (s, C-13b); 127.3 (s, C-12a); 127.2 (s, C-12b); 126.9 (d, C-14a); 124.7-124.5 (s, 4C, C-4); 115.8 (d, 4C, C-7); 111.4 (d, 4C, C-6); 110.2 (d, C-10b); 108.5 (d, C-10a); 103.0 (d, 4C, C-3); 102.2 (d, C-27b); 96.0 (d, C-27a); 90.8 (s, C-31); 55.7 (q, 4C, C-1); 45.3 (t, C-19b); 44.7 (t, C-19a); 39.7 (t, C-34); 32.8 (t, 2C, C-24a, 24b); 30.5-30.4 (t, 4C, C-22a, 22b, 23a, 23b); 30.1 (t, C-20b); 29.8 (t, C-20a); 28.2 (t, C-21a); 27.9 (t, C-21b); 23.6 (t, 2C, C-25a, 25b); 14.6-14.5 (q, 2C, C-26a, 26b); 12.6 (q, C-35). **HMBC correlations:** H-1→C-(2); H-3→C-(2, 4, 5, 7); H-6→C-(2, 4); H-7→C-(2, 3, 5); H-9a→C-(11a, 13a); H-10a→C-(8a, 11a, 12a); H-10b→C-(8b, 11b, 12b); H-14a→C-(8a, 12a, 16a); H-14b→C-(8b, 12b, 16b); H-15a→C-(13a, 17a); H-15b→C-(13b, 17b); H-16a→C-(12a, 14a, 18a); H-16b→C-(12b, 14b, 18b); H-19a→C-(11a, 18a, 20a, 21a); H-19b→C-(11b, 18b, 20b, 21b); H-20a→C-(19a, 21a, 22a); H-20b→C-(19b, 21b, 22b); H-26a→C-(24a, 25a); H-26b→C-(24b, 25b); H-27a→C-(17a, 18a, 28a, 29, 30); H-27b→C-(17b, 18b, 28b, 29, 30); H-34→C-(32, 33, 35); H-35→C-(34). **DQF-COSY correlations:** H-3→H-(7); H-6→H-(7); H-7→H-(3, 6); H-9a→H-(10a); H-9b→H-(10b); H-10a→H-(9a); H-10b→H-(9b); H-14a→H-(15a); H-14b→H-(15b); H-15a→H-(14a, 16a); H-15b→H-(14b, 16b); H-16a→H-(15a); H-16b→H-(15b); H-19a→H-(20a); H-19b→H-(20b); H-20a→H-(19a, 21a); H-20b→H-(19b, 21b); H-21a→H-(20a ); H-21b→H-(20b ); H-25a→H-(26a); H-25b→H-(26b); H-26a→H-(25a); H-26b→H-(25b); H-34→H-(35); H-35→H-(34).

Comment: Due to the asymmetric substitution at the squaraine moiety, two sets of differing NMR resonances can be observed for the hydrogens and carbons at many of the positions 8a-28a and 8b-28b, respectively. For the two carbazole substituents, only 4-fold intensity resonances were observed for each of the positions 1-7 by ^1^H and ^13^C NMR, probably due to the rapid rotation around the N-C bonds. ^1^H-^13^C HMBC long-range correlations were used to assign the resonances of all carbons. Only for the carbon at position 31 no long-range correlation was observed but the signal at 90.8 ppm observed in the 1D ^13^C NMR spectrum was assigned to this position, as already observed for **RSQ1** investigated earlier.^[S16]^ From the NMR experiments performed, it is not possible to unambiguously decide whether the chemical structure shown in the figure is correct or whether positions "a" should be replaced with "b" (and vice versa). In the present case, all NMR chemical shifts were tentatively assigned such that for positions 27b and 28b the ^1^H and ^13^C NMR chemical shifts appear at significantly higher frequencies than for positions 27a and 28a. Due to the amide carbonyl at position 32, this is quite plausible for the molecule shown above.

**Absorption** (CH_2_Cl_2_): λ_max_ = 1074 nm; λ_max_ = 612 nm; λ_max_ = 506 nm; **Absorption** (PhMe): λ_max_ = 1134 nm (64000 M^-1^cm^-1^); λ_max_ = 656 nm; λ_max_ = 506 nm.

**HR-ESI-MS**: [M]^2+^ C_77_H_75_N_5_O_6_S_2_; calc 614.7577 m/z; found 614.7597 m/z (3.3 Δppm).

Attempts to crystallize **RSQ3** were not successful.

**(Z)-3-(2-(dicyanomethylene)-3-ethyl-4-oxothiazolidin-5-ylidene)-2-ethoxy-4-oxocyclobut-1-en-1-olate (7)**

A 100 mL Schlenk tube was charged with dry PhMe (40 mL), 2-(3-ethyl-4-oxothiazolidin-2-ylidene)malononitrile (3.41 g, 17.6 mmol, 1.0 eq), 3,4-diethoxycyclobut-3-ene-1,2-dione (3.0 g, 17.6 mmol, 1.0 eq) and triethylamine (NEt_3_, 2.5 mL, 17.6 mmol, 1.0 eq). The deep brown solution was stirred at 50 °C for 2 h after which a brown precipitate was formed. The precipitate was collected by filtration, was washed with cold hexane and dried *in vacuo*. The squarate **7** (6.1 g, 14.6 mmol) was used in subsequent steps without further purification.

**(3Z,4Z)-3-(2-(dicyanomethylene)-3-ethyl-4-oxothiazolidin-5-ylidene)-4-((1-octylbenzo[cd]indol-1-ium-2-yl)methylene)-2-((E)-(1-octylbenzo[cd]indol-2(1H)-ylidene)methyl)cyclobut-1-en-1-olate (DCRSQ1)**

A Schlenk round bottom flask equipped with a septum was charged with 3 Å molecular sieve, dry nBuOH (10 mL) and dry PhMe (10 mL). 2-Methyl-1-octyl-benz[*cd*]indolium iodide **8**^[S10]^ (210 mg, 516 μmol, 2.2 eq) and squarate **7** (100 mg, 239 μmol, 1 eq) was added to the reaction mixture and the dark green solution was stirred at 60 °C for 18 h. The crude reaction mixture was cooled to r.t. and diluted with CH_2_Cl_2_ (50 mL). Celite® was added to the dark green solution and the solvents were removed *in vacuo*. **DCRSQ1** was purified by flash column chromatography (SiO_2_, linear gradient 100% heptane to 50% EtOAc in heptane). Fractions containing the title compound were combined and the solvent removed *in vacuo*. The title compound was dissolved in minimal amounts of CH_2_Cl_2_ and precipitated from heptane. Filtration and drying under high vacuum delivered **DCRSQ1** (82 mg, 101 μmol, 42%) as a dark-purple solid.

**^1^H NMR** (400.2 MHz, CDCl_3_): δ = 8.86 (d (br), *J* = 7.2, 1H); 8.67 (d, *J* = 7.5, 1H); 8.19 (s (br), 1H); 8.00 (d, *J* = 8.0, 1H); 7.94 (d, *J* = 8.0, 1H); 7.80 (m, 2H); 7.64 (d, *J* = 8.2, 1H); 7.55 (d, *J* = 8.0, 1H); 7.51 (m, 1H); 7.44 (m, 1H); 7.05 (d, *J* = 7.5, 1H); 6.85 (d, *J* = 7.3, 1H); 6.12 (s, 1H); 4.15 (t, *J* = 7.5, 2H); 3.93 (t, *J* = 7.0, 2H); 3.87 (q, *J* = 7.1, 2H); 1.91 (m, 2H); 1.84 (m, 2H); 1.55-1.25 (m, 20H); 1.20 (t, *J* = 7.1, 3H); 0.87 (m, 6H). **^13^C NMR** (100.6 MHz, CDCl_3_): δ = 175.5 (s); 166.2 (s); 165.2 (s); 163.4 (s); 161.5 (s); 159.5 (s); 152.3 (s); 150.4 (s); 141.1 (s); 140.7 (s); 132.4 (d); 130.8 (d, 2C); 129.9 (d); 129.8 (s, 2C); 129.7 (s); 129.7 (d); 129.6 (s); 129.5 (d); 128.7 (d, 2C); 125.4 (s); 125.3 (s); 123.1 (d); 121.8 (d); 116.2 (s); 115.3 (s); 108.8 (d); 107.3 (d); 100.3 (d); 93.8 (d); 84.7 (s); 46.2 (s); 44.9 (t); 44.3 (t); 39.2 (t); 32.0 (t); 31.9 (t); 29.4-29.3 (t, 5C); 28.8 (t); 27.3 (t, 2C); 22.8 (t, 2C); 14.3 (q); 14.2 (q, 2C).

**Absorption** (CH_2_Cl_2_): λ_max_ = 990 nm; λ_max_ = 605 nm; λ_max_ = 510 nm. **Absorption** (PhMe): λ_max_ = 1035 nm (54000 M^-1^cm^-1^); λ_max_ = 624 nm; λ_max_ = 511 nm.

**HR-ESI-MS**: [M+H]^+^ C_52_H_54_N_5_O_2_S; calc 812.3993 m/z; found 812.3977 m/z (1.9 Δppm).

**(3Z,4Z)-4-((6-(bis(4-methoxyphenyl)amino)-1-octylbenzo[cd]indol-1-ium-2-yl)methylene)-2-((E)-(6-(bis(4-methoxyphenyl)amino)-1-octylbenzo[cd]indol-2(1H)-ylidene)methyl)-3-(2-(dicyanomethylene)-3-ethyl-4-oxothiazolidin-5-ylidene)cyclobut-1-en-1-olate (DCRSQ2)**

A Schlenk round bottom flask equipped with a septum was charged with 3 Å molecular sieve, dry n-BuOH (10 mL) and dry PhMe (10 mL). **4** (514 mg, 788 μmol, 2.2 eq) and **7** (150 mg, 358 μmol, 1 eq) was added to the reaction mixture and the dark green solution was stirred at 60 °C for 2 h. The crude reaction mixture was cooled to r.t. and diluted with CH_2_Cl_2_ (100 mL). Celite® was added to the dark green solution and the solvents were removed *in vacuo*. The title compound was purified by flash column chromatography (SiO_2_, linear gradient 100% heptane to 30% EtOAc in heptane) and the solvent removed *in vacuo*. The title compound was dissolved in minimal amounts of CH_2_Cl_2_ and precipitated from heptane. Filtration and drying under high vacuum delivered **DCRSQ2** (86 mg, 68 μmol, 19%) as a dark-purple solid.

Chemical structure of **DCRSQ2** with numbering of positions.

**^1^H NMR** (400.2 MHz, CDCl_3_): δ = 8.47 (d (br), *J* = 7.1, 1H, H-14b); 8.30 (d, *J* = 7.5, 1H, H-14a); 8.10 (s, 1H, H-25b); 7.68 (d, *J* = 8.2, 1H, H-12b); 7.64 (d, *J* = 8.2, 1H, H-12a); 7.49 (m, 2H, H-13a, 13b); 7.00 (m, 11H, H-4, 7a, 7b, 8b); 6.90 (d, *J* = 8.0, 1H, H-8a); 6.79 (m, 8H, H-3); 6.18 (s, 1H, H-25a); 4.26 (q, *J* = 7.1, 2H, H-35); 4.21 (t, *J* = 7.8, 2H, H-17b); 4.11 (t, *J* = 7.1, 2H, H-17a); 3.79 (s, 12H, H-1); 1.96 (m, 2H, H-18b); 1.92 (m, 2H, H-18a); 1.53 (m, 4H, H-19a, 19b); 1.5-1.2 (m, 19H, H-20a-23a, 20b-23b, 36); 0.87 (m, 6H, H-24a, 24b).

**^13^C NMR** (100.6 MHz, CDCl_3_): δ = 176.4 (s, C-27); 165.9 (s, C-30); 163.7 (s, C-26b); 163.6 (s, C-31); 159.5 (s, C-28); 158.9 (s, C-26a); 155.9 (s, 2C, C-2); 155.5 (s, 2C, C-2); 150.9 (s, C-16b); 149.3 (s, C-16a); 143.9 (s, C-6b); 142.8 (s, 2C, C-5); 142.6 (s, 2C, C-5); 142.0 (s, C-6a); 137.6 (s, C-9a); 136.5 (s, C-9b); 131.9 (d, C-14b); 130.6 (s, C-15a); 130.5 (s, C-15b); 130.0 (d, C-14a); 129.3 (d, C-12b); 128.6 (d, 2C, C-13a, 13b); 128.1 (d, C-12a); 127.1 (s, C-10a); 127.0 (s, C-11a); 126.9 (s, C-10b); 126.4 (s, C-11b); 125.4 (d, 4C, C-4); 125.0 (d, C-7a); 124.9 (d, 4C, C-4); 124.1 (d, C-7b); 116.5 (s, C-33); 115.8 (s, C-34); 115.0 (d, 4C, C-3); 114.8 (d, 4C, C-3); 110.5 (d, C-8b); 108.4 (d, C-8a); 99.3 (d, C-25b); 92.8 (d, C-25a); 84.6 (s, C-29); 55.7 (q, 4C, C-1); 46.1 (s, C-32); 45.0 (t, C-17b); 44.5 (t, C-17a); 39.6 (t, C-35); 32.0 (t, 2C, C-22a, 22b); 29.5 – 29.3 (t, 5C, C-20a, 21a, 18b, 20b, 21b); 29.0 (t, C-18a);27.4 (t, C-19a); 27.3 (t, C-19b); 22.8 (t, 2C, C-23a, 23b); 14.5 (q, C-36); 14.3 & 14.2 (q, 2C, C-24a, 24b). **^1^H-^1^H DQF-COSY correlations:** H-3→H-(4); H-4→H-(3); H-7a→H-(8a); H-8a → H-(7a); H-12a → H-(13a); H-13a → H-(12a, 14a); H-14a → H-(13a); H-17a → H-(18a); H-18a → H-(17a, 19a); H-19a → H-(18a); H-35 → H-(35); H-36 → H-(35). **^1^H-^13^C HMBC correlations:** H-1 → C-(2); H-3 → C-(2, 3, 4, 5); H-4 → C-(2, 3, 4, 5); H-7a → C-(6a, 9a, 11a); H-7b → C-(6b, 9b, 11b); H-8a → C-(6a, 9a, 10a); H-8b → C-(6b, 9b, 10b); H-12a → C-(6a, 9a(w), 10a, 14a); H-12b → C-(6b, 9b(w), 10b, 14b); H-13a → C-(11a, 12a(w), 15a, 16a(w)); H-13b → C-(11b, 14b(w), 15b, 16b(w)); H-14a → C-(10a, 12a, 16a); H-14b → C-(10b, 12b); H-17a → C-(9a, 16a, 18a, 19a); H-17b → C-(9b, 16b, 18b, 19b); H-18a → C-(17a, 19a, 20a); H-18b → C-(17b, 19b, 20b); H-24a → C-(22a, 23a); H-24b → C-(22b, 23b); H-25a → C-(15a, 16a, 26a, 27, 28); H-25b → C-(15b, 16b, 26b, 27, 28); H-35 → C-(30, 31, 36); H-36 → C-(35).

Comment: As previously noted for **RSQ3**, two distinct sets of NMR resonances were observed for the hydrogens and carbons at positions 6a-26a and 6b-26b, respectively. NMR resonances at positions 1-5 of the two bis(4-methoxyphenyl)amine substituents were partly resolved but could not be assigned to individual parts "a" and "b" of the chemical structure. For resonance assignment, ^1^H-^13^C HMBC long-range correlations were employed, using the olefinic proton signals H-25a (δ 1H = 8.10 ppm) and H-25b (δ 1H = 6.18 ppm) as reference points. In the case of part “a”, positions 15a and 16a were assigned, with correlations to positions 26a, 27, and 28, providing further insight into the squaraine unit’s substituent. The HMBC correlation between H-17a and C-16a, as well as C-9a, was subsequently used to initiate the subsequent assignment of the whole indolone substituent. No long-range correlations were observed for carbons at positions 29, 32, 33, and 34. Utilizing the information from **RSQ2**, the signal at 84.8 ppm in the 1D ^13^C NMR spectrum was assigned to position 29. The signal for a quaternary carbon at 46.1 ppm was assigned to C-32 based on NMR chemical shift considerations. Finally, C-33 and C-34 were assigned to the two CN groups in the molecule, again supported by NMR chemical shift arguments. The NMR experiments do not definitively confirm whether the shown chemical structure is correct or if positions “a” and “b” should be swapped. However, based on tentative assignments, the ^1^H and ^13^C NMR shifts for positions 25b and 26b appear at significantly higher frequencies than for 25a and 26a, which is plausible given the amide carbonyl at position 30.

**Absorption** (CH_2_Cl_2_): λ_max_ = 1106 nm; λ_max_ = 660 nm; λ_max_ = 518 nm. **Absorption** (PhMe): λ_max_ = 1142 nm (117000 M^-1^cm^-1^); λ_max_ = 668 nm; λ_max_ = 516 nm.

**HR-ESI-MS**: [M+H]^+^ C_80_H_80_N_7_O_6_S; calc 1266.5885 m/z; found 1266.5830 m/z (4.3 Δppm).

**(3Z,4Z)-3-(2-(dicyanomethylene)-3-ethyl-4-oxothiazolidin-5-ylidene)-4-((6-(3,6-dimethoxy-9H-carbazol-9-yl)-1-octylbenzo[cd]indol-1-ium-2-yl)methylene)-2-((E)-(6-(3,6-dimethoxy-9H-carbazol-9-yl)-1-octylbenzo[cd]indol-2(1H)-ylidene)methyl)cyclobut-1-en-1-olate (DCRSQ3)**

A Schlenk round bottom flask equipped with a septum was charged with 3 Å molecular sieve, dry nBuOH (10 mL) and dry PhMe (10 mL). Iminium salt **6** (320 mg, 492 μmol, 2.2 eq) and **7** (100 mg, 239 μmol, 1 eq) were added to the reaction mixture and the dark green solution was stirred at 60 °C for 2 h. The crude reaction mixture was cooled to r.t. and diluted with CH_2_Cl_2_ (100 mL). Celite® was added to the dark blue solution and the solvents were removed *in vacuo*. **DCRSQ3** was purified by flash column chromatography (SiO_2_, linear gradient 100% heptane to 40% EtOAc in heptane). Fractions containing the title compound were combined and the solvent removed *in vacuo*. The title compound was dissolved in minimal amounts of CH_2_Cl_2_ and precipitated from heptane. Filtration and drying under high vacuum delivered **DCRSQ3** (53 mg, 42 μmol, 18%) as a dark-purple solid.

**^1^H NMR** (400.2 MHz, CDCl_3_): δ = 8.63 (d (br), *J* = 7.1, 1H); 8.46 (d, *J* = 7.5, 1H); 8.33 (s (br), 1H); 7.75 – 7.55 (m, 9H); 7.51 (d, *J* = 8.2, 1H); 7.34 (d, *J* = 7.7, 1H); 7.21 (d, *J* = 7.8, 1H); 7.08+7.06 (d, *J* = 8.9, 4H); 6.99 (dd, *J* = 8.9, 2.4, 4H); 6.36 (s, 1H); 4.37 (t, *J* = 7.6, 2H); 4.31 (q, *J* = 7.1, 2H); 4.26 (t, *J* = 7.8, 2H); 3.96 (s, 12H); 2.05 (m, 4H); 1.59 (m, 4H); 1.5 – 1.2 (m, 19H); 0.89 (m, 6H). **^13^C NMR** (100.6 MHz, CDCl_3_): δ = 175.5 (s); 166.3 (s); 166.0 (s); 164.0 (s); 161.6 (s); 159.1 (s); 154.5 (s, 2C); 154.4 (s, 2C); 152.5 (s); 150.8 (s); 141.0 (s); 140.4 (s); 137.9 (s, 2C); 137.8 (s, 2C); 132.8 (d); 131.9 (s); 131.2 (d); 130.6 (s, 2C); 130.5 (s); 130.1 (d); 129.9 (d); 128.5 (d); 128.4 (d); 128.3 (d); 127.7 (s); 127.5 (s); 127.3 (d); 126.9 (s); 126.8 (s); 124.0 (s, 2C); 123.9 (s, 2C); 116.2 (s); 115.4 (d, 4C); 115.3 (s); 111.1 (d, 2C); 111.0 (d, 2C); 109.2 (d); 107.7 (d); 103.2 (d, 4C); 100.8 (d); 94.4 (d); 85.5 (s); 56.3 (q, 4C); 47.1 (s); 45.3 (t); 44.9 (t); 39.8 (t); 32.0 (t, 2C); 29.5-29.3 (t, 5C); 29.1 (t); 27.4 (t); 27.3 (t); 22.8 (t, 2C); 14.5 (q); 14.3 (q, 2C).

**Absorption** (CH_2_Cl_2_): λ_max_ = 1050 nm; λ_max_ = 612 nm; λ_max_ = 506 nm. **Absorption** (PhMe): λ_max_ = 1096 nm (67000 M^-1^cm^-1^); λ_max_ = 645 nm; λ_max_ = 506 nm.

**HR-ESI-MS**: [M]^2+^ C_80_H_75_N_7_O_6_S; calc 630.7744 m/z; found 630.7741 m/z (0.5 Δppm).

# TGA Data


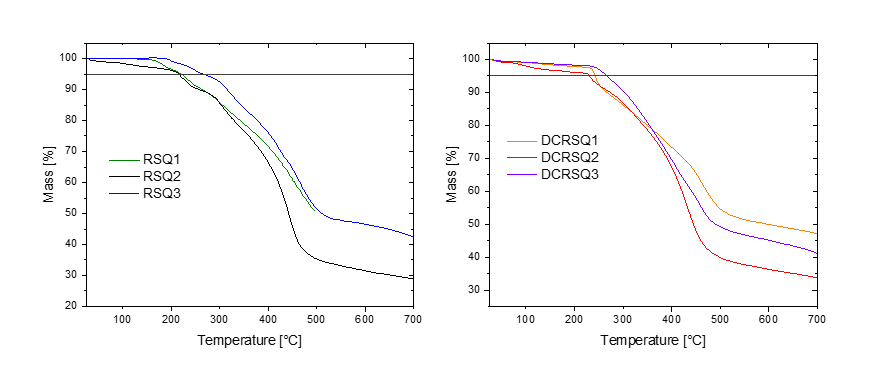


**Figure S1.** Thermal gravimetric analysis of SQ dyes.

# Optical Spectroscopy Data and Calculations


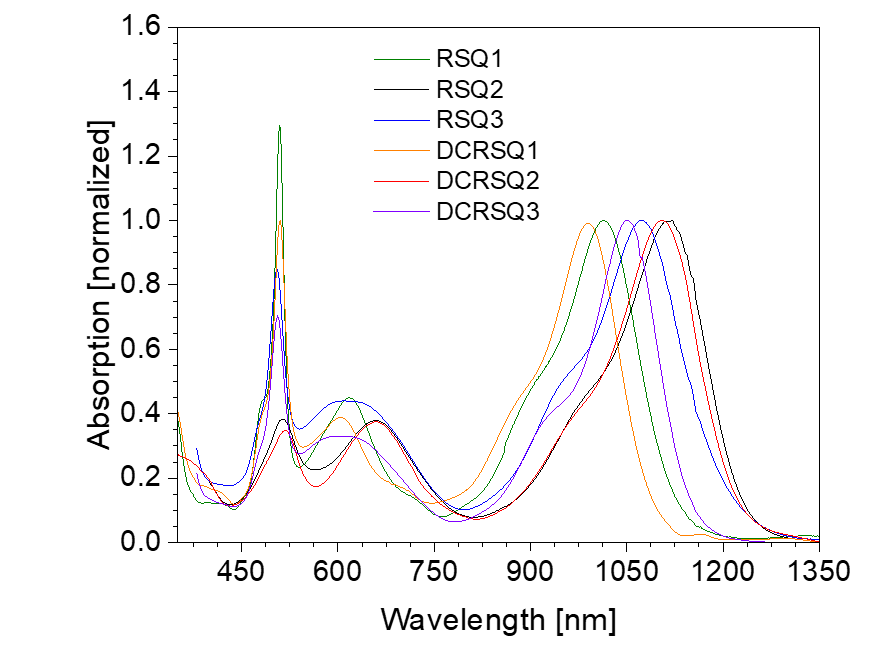


**Figure S2.** Absorption spectra of SQ dyes in dichloromethane.


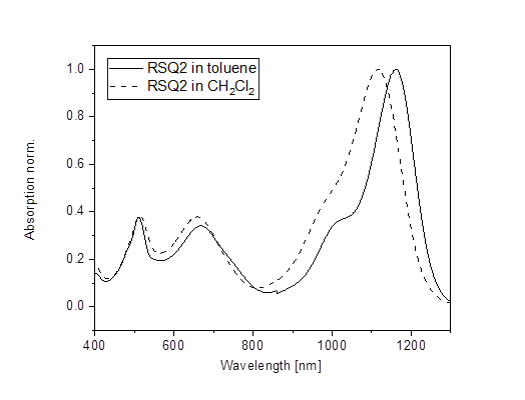


**Figure S3.** Absorption spectra of **RSQ2** in toluene and dichloromethane.


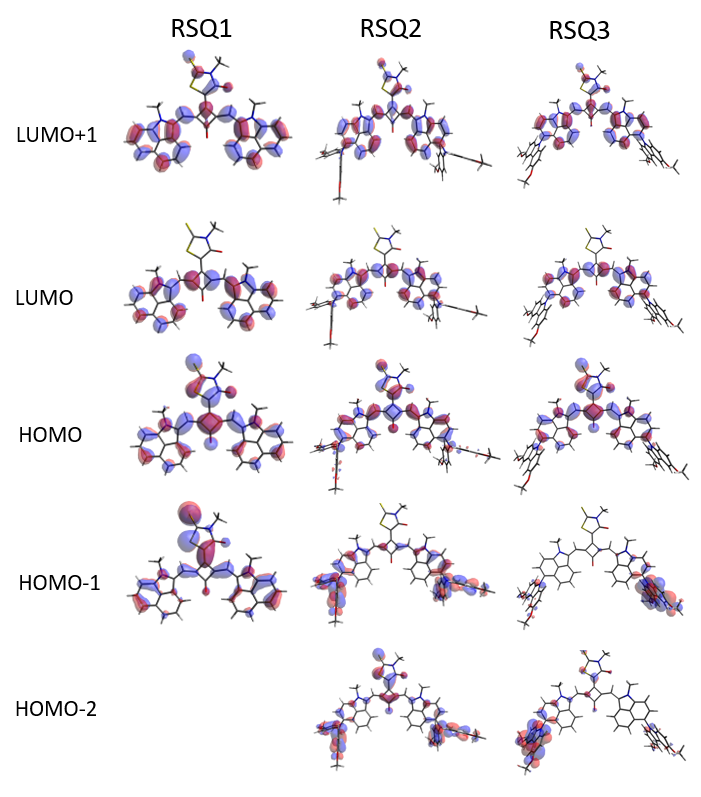


**Figure S4.** Electron density distribution of the relevant molecular orbitals for rhodanine SQs. The difference between the calculated orbital energies for HOMO-1 and HOMO-2 is < 0.7 eV.


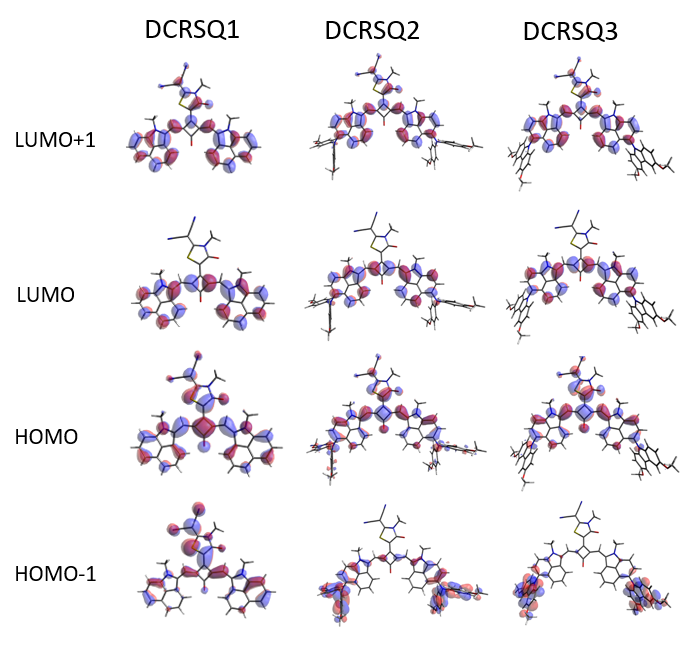


**Figure S5.** Electron density distribution of the relevant molecular orbitals for dicyano-rhodanine SQs.


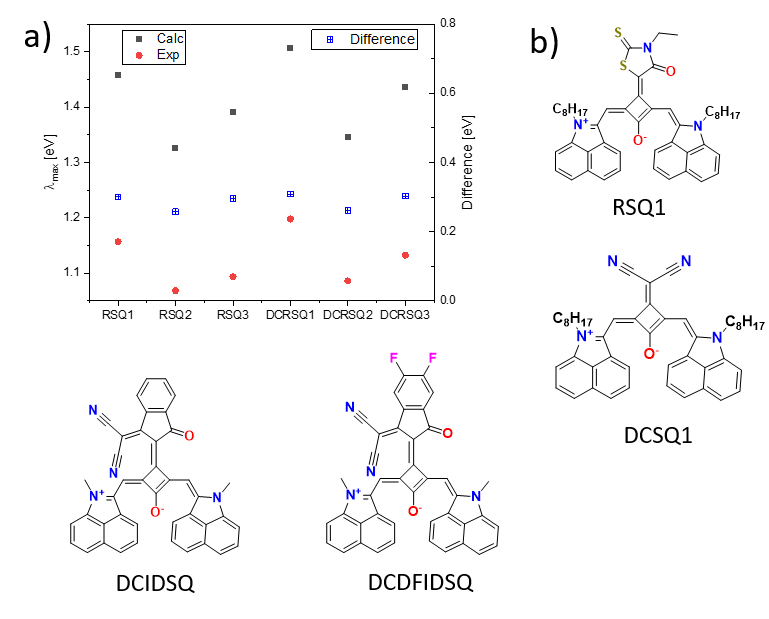


**Figure S6.** a) The calculated S0->S1 transition energy for all synthesized dyes is larger by ≈0.3 eV than the experimental value. The blue points indicate the difference between the calculated and the experimental value. b) Assuming that this difference is constant for other NIR SQ dyes as well, we calculated λ_max_ for other dyes (named DCSQ1, DCIDSQ and DCDFIDSQ) containing conceivable strong acceptor groups:

**RSQ1** Calculated 1.46 eV, subtracting 0.3 eV yields 1.16 eV ≡ 1069 nm. The experimental absorption is at 1072 nm.

**DCSQ1** Calculated 1.60 eV, subtracting 0.3 eV yields 1.30 eV ≡ 954 nm. The experimental absorption is at 958 nm.^[S1]^

**DCIDSQ** containing the dicyanoindanone acceptor group.

Calculated 1.69 eV, subtracting 0.3 eV yields 1.39 eV ≡ 892 nm.

**DCDFIDSQ** containing the difluoro-dicyanoindanone acceptor group.

Calculated 1.71 eV, subtracting 0.3 eV yield 1.41 eV ≡ 879 nm.

# Cyclic Voltammetry Data


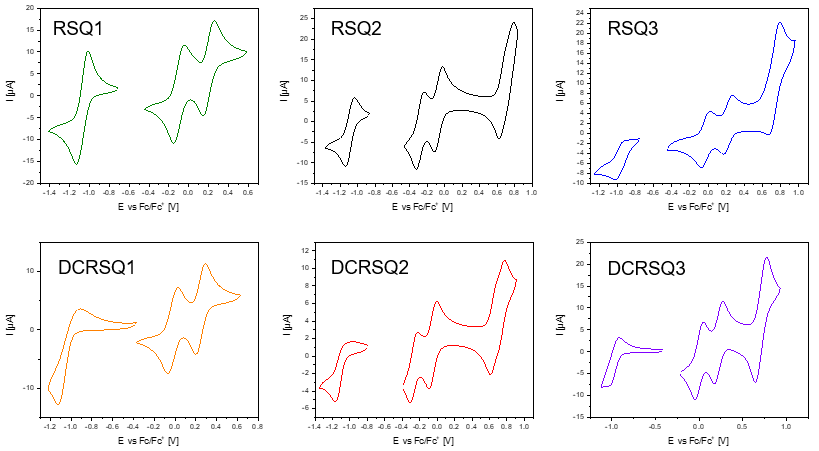


**Figure S7.** Cyclic voltammograms for SQ dyes.

**Table S1.** Data analysis from cyclic voltammgrams shown in Figure S7.

|  | E_ox1_ [V] | E_ox2_ [V] | E_ox3_ [V] | E_red1_ [V] | $E_{HOMO}$ [eV] | $E_{LUMO}$ [eV] | $E_{gap,cv}$ [eV] |
| --- | --- | --- | --- | --- | --- | --- | --- |
| **RSQ1** | -0.10  -0.08^a)^ | 0.20  0.21^a)^ |  | -1.08  -1.07^a)^ | -4.93  -5.02^a)^ | -4.10  -4.12^a)^ | 0.83  0.90^a)^ |
| **RSQ2** | -0.28 | -0.07 | 0.71 | -1.09 | -4.74 | -4.07 | 0.67 |
| **RSQ3** | -0.03 | 0.22 | 0.73 | -0.90 | -5.00 | -4.23 | 0.77 |
| **DCRSQ1** | -0.02 | -0.25 |  | -1.02 | -5.00 | -4.11 | 0.89 |
| **DCRSQ2** | -0.27 | -0.05 | 0.69 | -1.08 | -4.75 | -4.04 | 0.71 |
| **DCRSQ3** | 0.00 | 0.23 | 0.71 | -0.97 | -5.03 | -4.20 | 0.83 |

a) Data from reference [S16]. We included **RSQ1** into the measurement series for a consistent data set obtained under the same experimental conditions.

# Photodetector Data


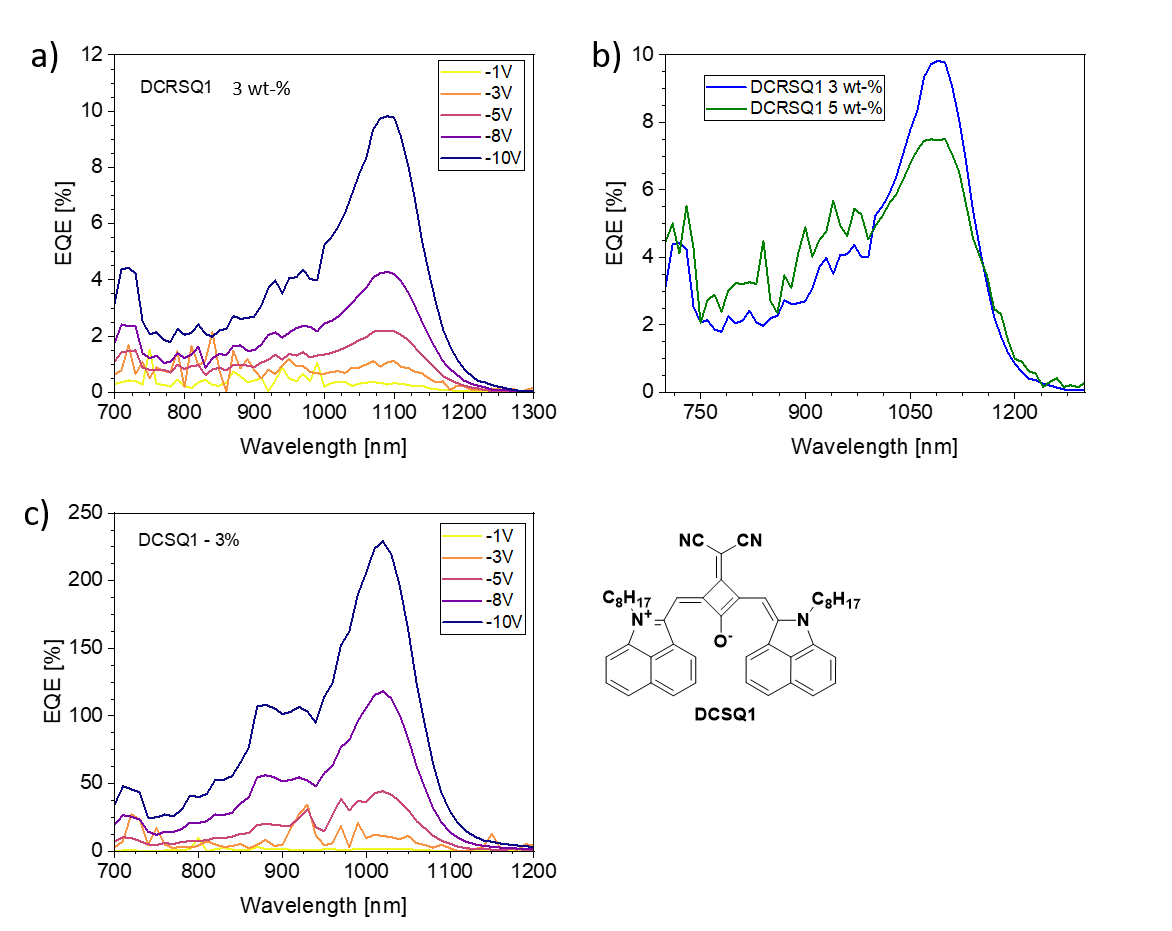


**Figure S8.** a) The strong and non-linear EQE increase with increasing voltage bias for the OPD using **DCRSQ1** confirms the device functionality. However, EQE values are lower than for **RSQ2**. This might be related to the deeper LUMO level of **DCRSQ1** compared to **RSQ2** (Table 1, main text), resulting in less efficient photocharge generation at the dye/PCBM interface. b) **DCRSQ1** exhibits also the smallest extinction coefficient among our dye series. To increase the light absorption the dye concentration was increased to 5 wt-%. However, this resulted in a decreased EQE, which can be explained with the formation of percolating paths for holes and increased charge extraction. c) As a second SQ we tested the dye **DCSQ1** that contains the dicyanomethylene acceptor group. In our hands, **DCSQ1** is the most easy accessible among all SQ dyes we synthesized so far.^[S1]^ The EQE reaches over 200% at -10 V, indicating photomultiplication gain.


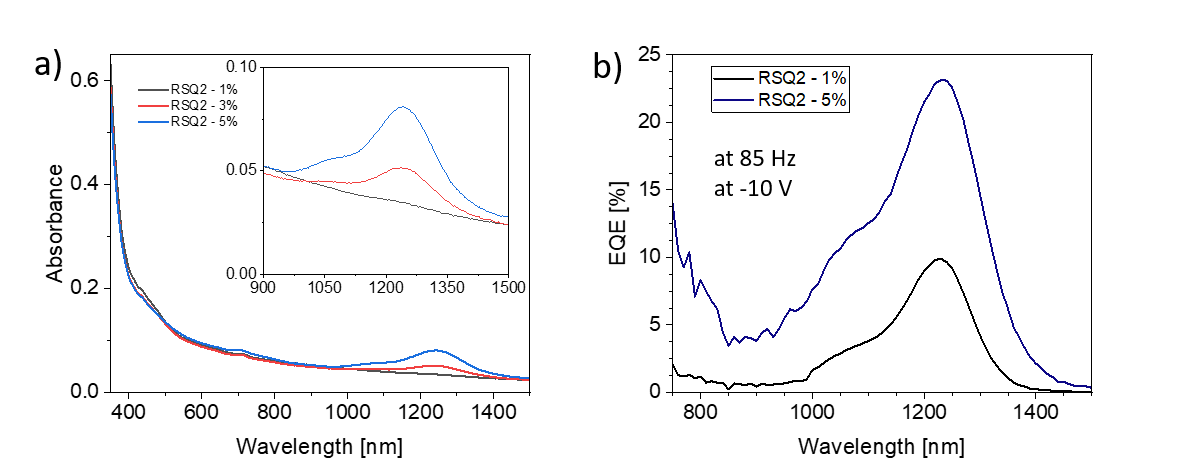


**Figure S9.** a) Absorbance spectra of PCBM:**RSQ2** (1, 3, and 5 wt-%) films, coated from CHCl_3_. b) EQE spectra for **RSQ2** concentrations of 1 and 5 wt-%.

**
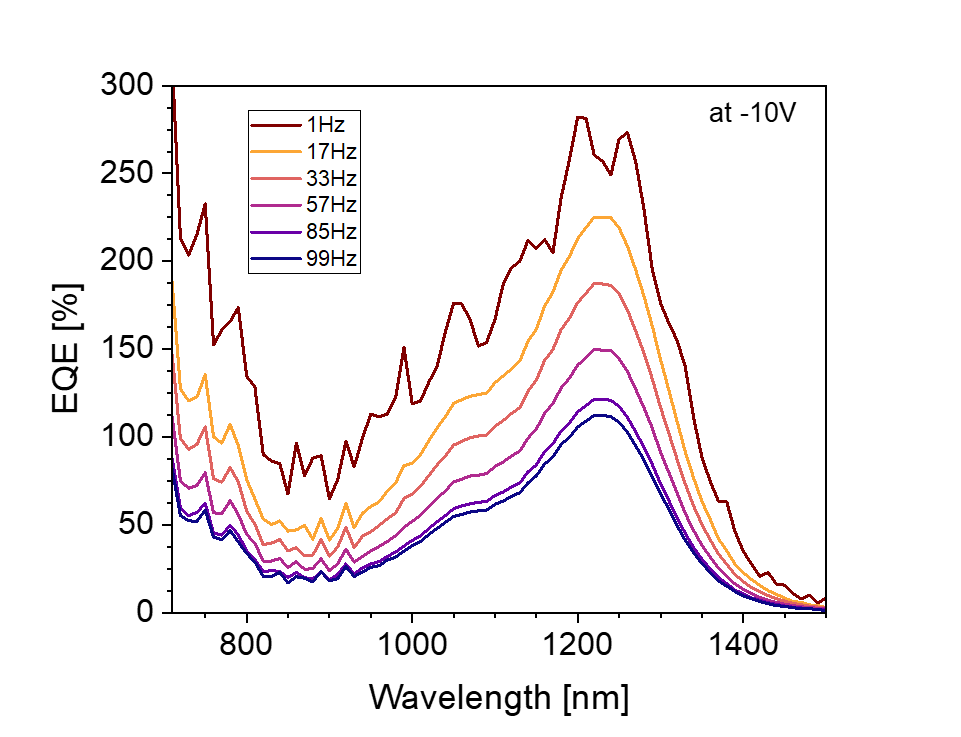
**

**Figure S10.** EQEs for different chopper frequency. Due to the slow device response, the EQE increases when decreasing the light modulation frequency. For a small chopper rotation rate, suppression of the dark current is poor and the signal gets noisy.

**Figure S11.** EQE spectra – displayed is the measured photocurrent - for varying light intensity, at -10 V and at 85 Hz. The insert magnifies the EQE for light intensity 10% down to dark. For the highest light intensity (3.26 W m^-2^ at 1240 nm), the EQE was 27.3%.


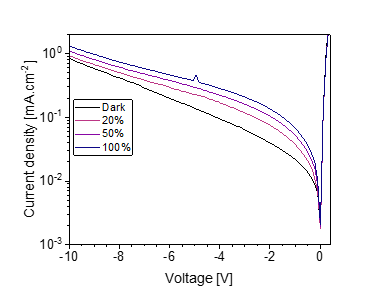


**Figure S12.** J-V trends of the ITO/NiO 5 nm/PCBM:**RSQ2** (3 wt-%) 100 nm/BCP/TPBi/Al OPD. Light at 1200 nm.


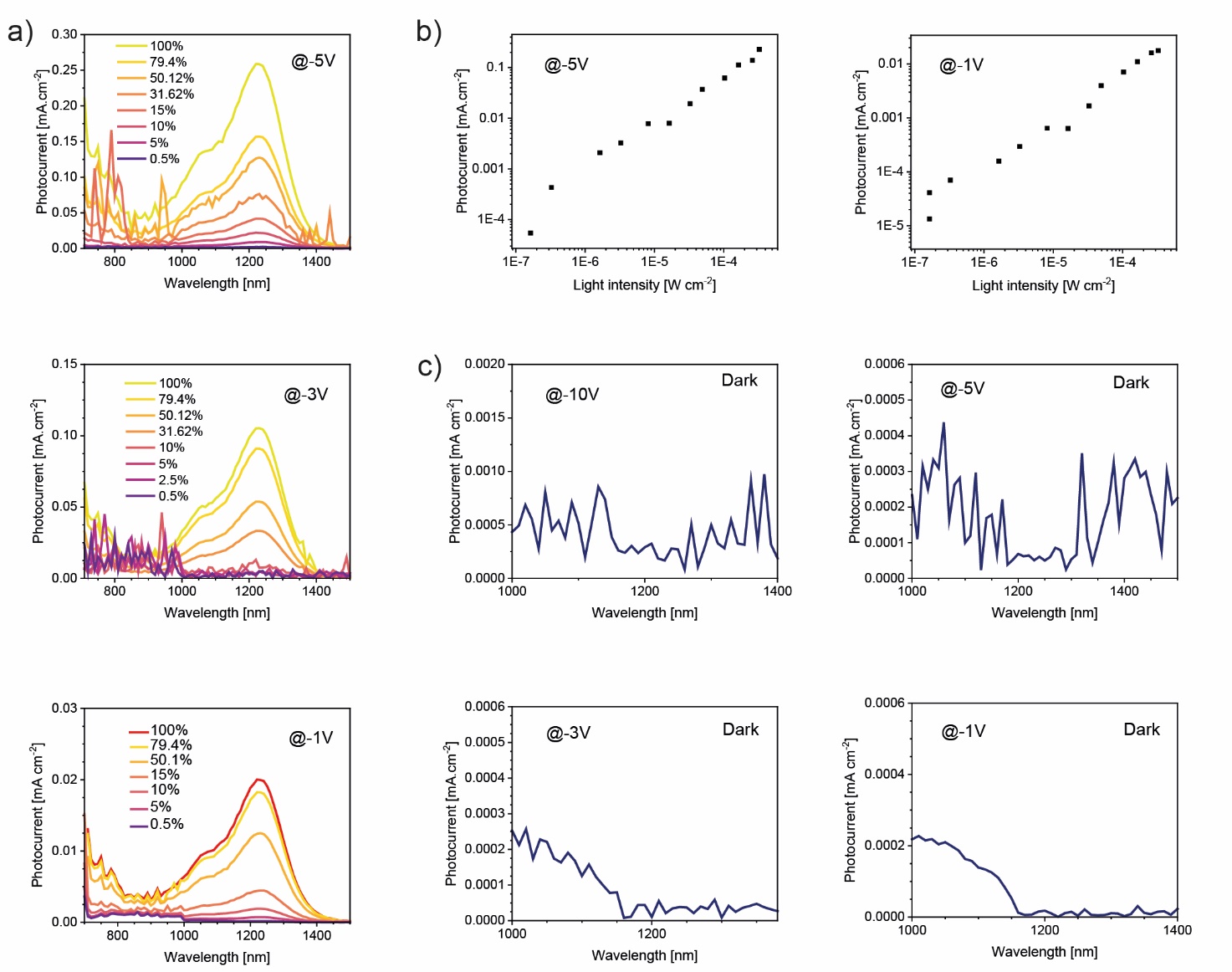


**Figure S13.** a) EQE for different light intensity at 85 Hz, at -5 V, -3 V and -1 V. Corresponding responsivities at 100% light are R = 0.78, 0.32, and 0.06 A W^-1^. b) In the voltage range -1 V to -5 V, the dynamic response is fairly linear over a light intensity range of 3 orders of magnitude. c) In the EQE measurements, the dark current drops over 2 orders of magnitude with decreasing voltage (≈1 x 10^-6^ A cm^-2^ at -10 V, 2 x 10^-8^ A cm^-2^ at -1 V).


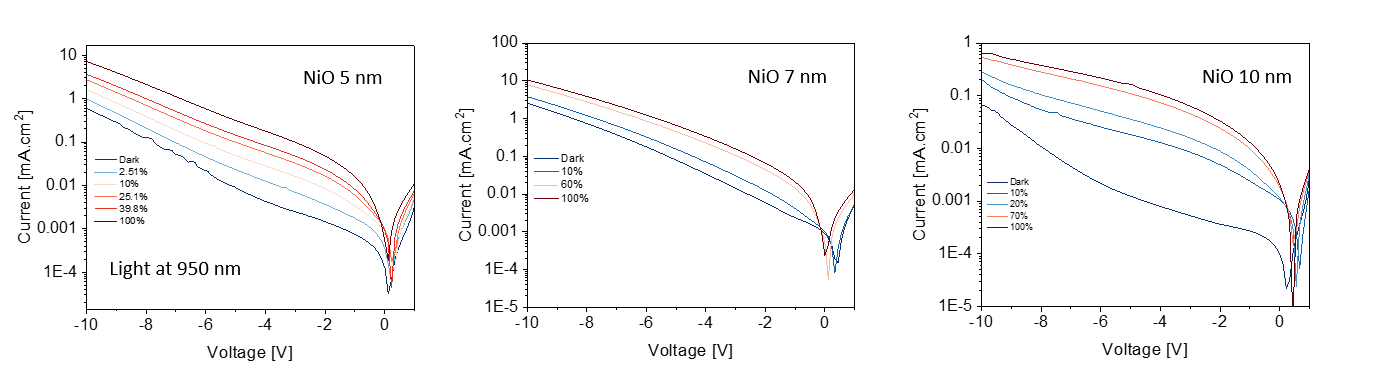


**Figure S14.** Representative J-V trends for different NiO layer thickness. For a thickness of 5 nm and 7 nm, dark currents at -10 V varied from 0.5-2 mA cm^-2^, and photocurrents varied from 5-10 mA cm^-2^. Increasing the NiO thickness to 10 nm resulted in a decreased dark current, but the photocurrent dropped to below 1 mA cm^-2^. For these experiments we used the SQ dye DCSQ1 from reference [S1], which is composed of benzindole donors and the dicyanomethylene-substituted squarine acceptor unit.


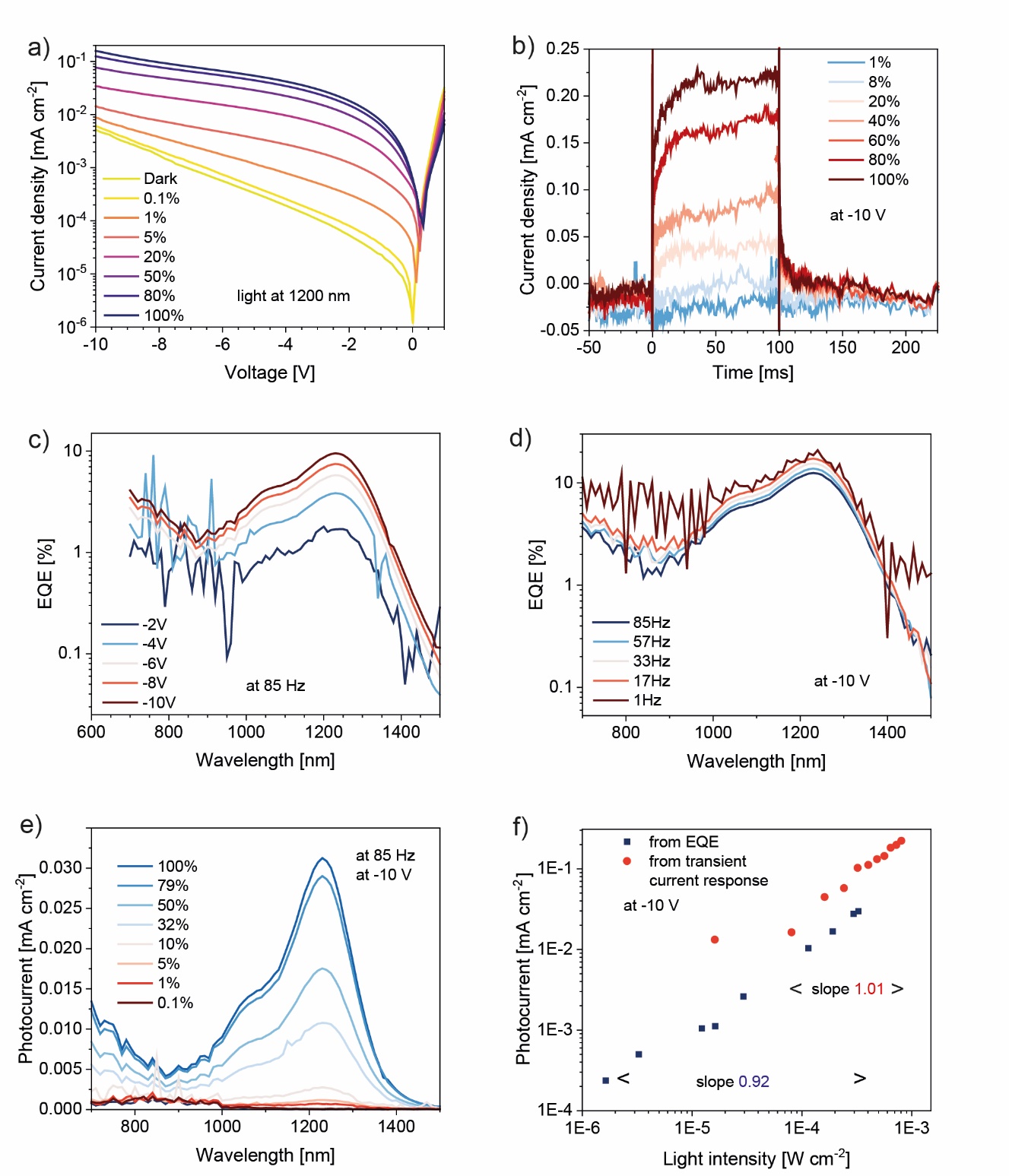


**Figure S15.** Performance of the ITO/NiO 5 nm/PCBM:**RSQ2** (3 wt-%) **200 nm**/BCP/TPBi/Al OPD. a) J-V trends for different light intensity. b) Current response to light pulses (at 1200 nm) of varying intensity. Current rise and fall times (≈ 20 ms) for the thick and thin device (Fig. 3c, main text) are the same. c) EQE as function of voltage bias and d) EQE when decreasing the chopper frequency. e) EQE for varying light intensity. At 100% light intensity, the EQE is 9.4%. f) OPD linear dynamic range at -10 V from EQE and transient photocurrent response.

# NMR Spectra


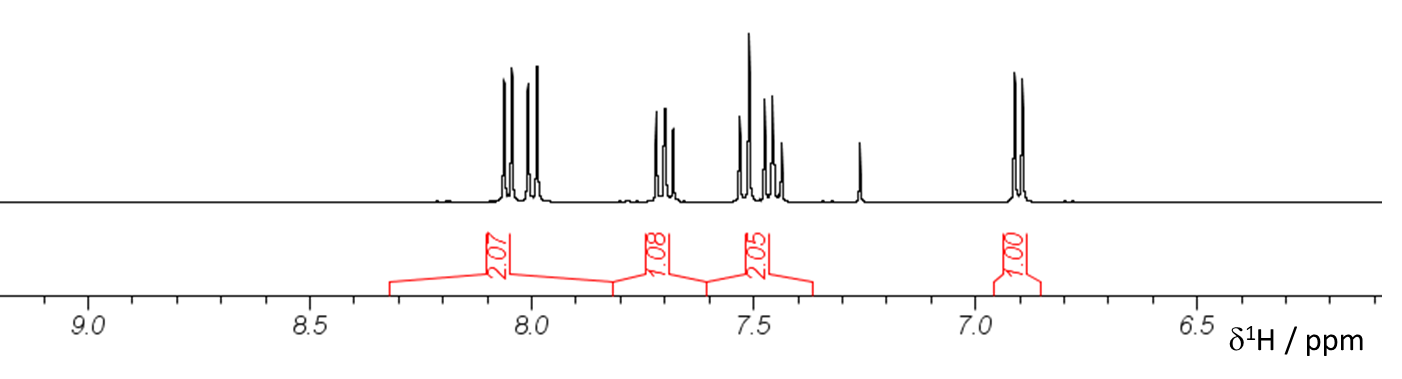


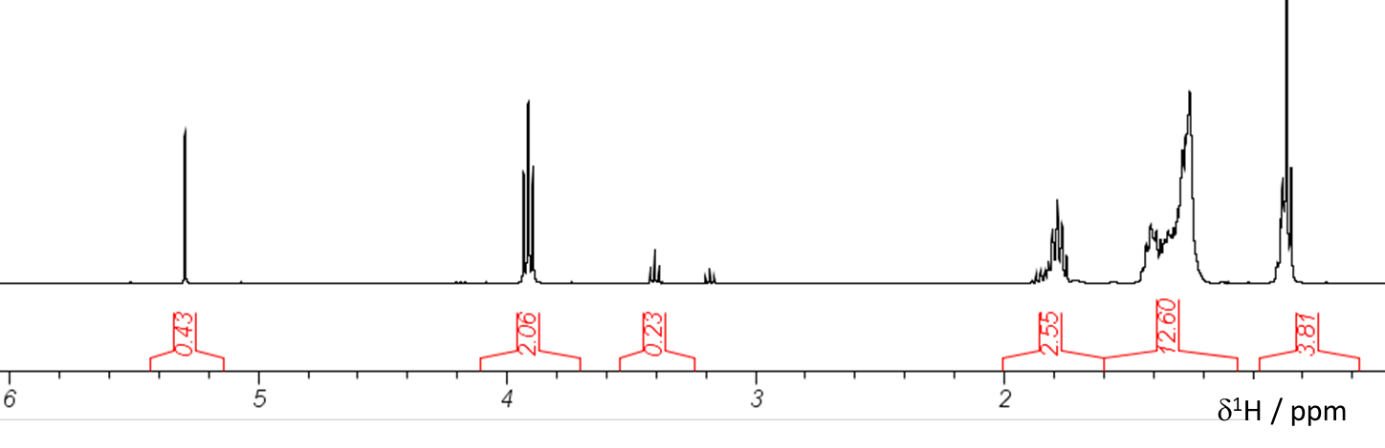


^1^H NMR spectrum of 1 (CDCl_3_, 298K).


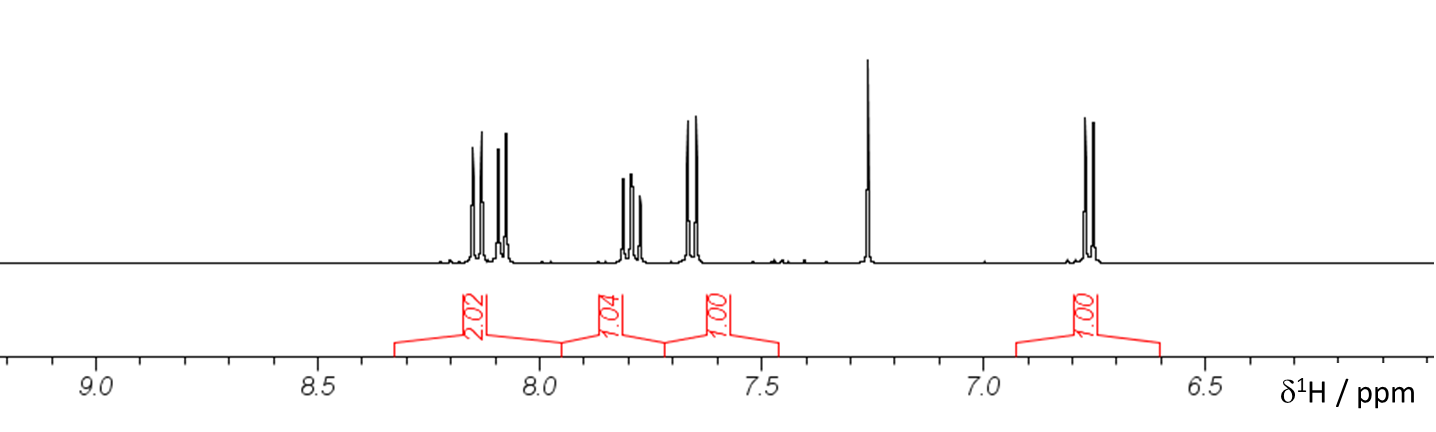


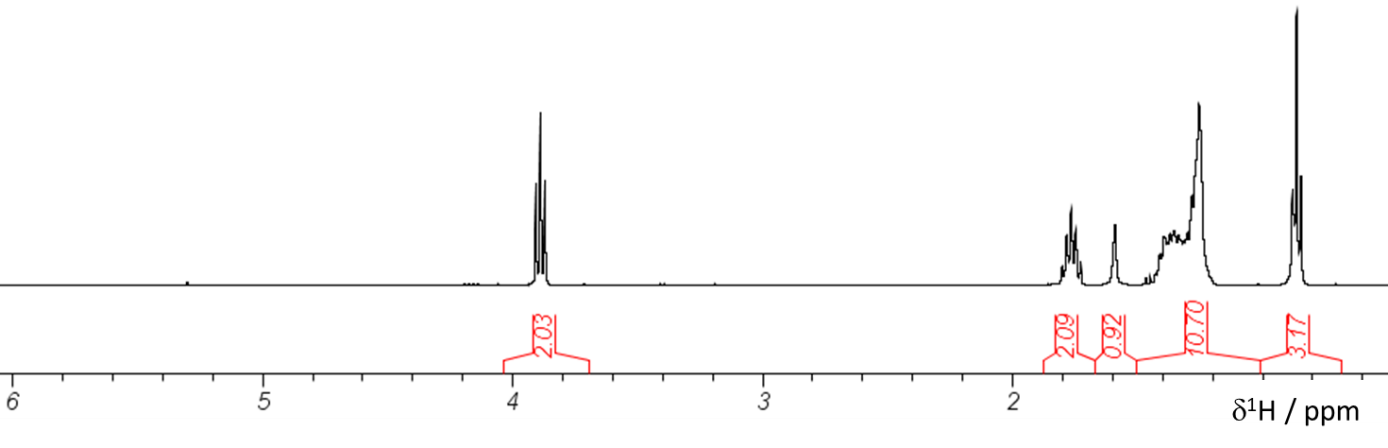


^1^H NMR spectrum of 2 (CDCl_3_, 298K).


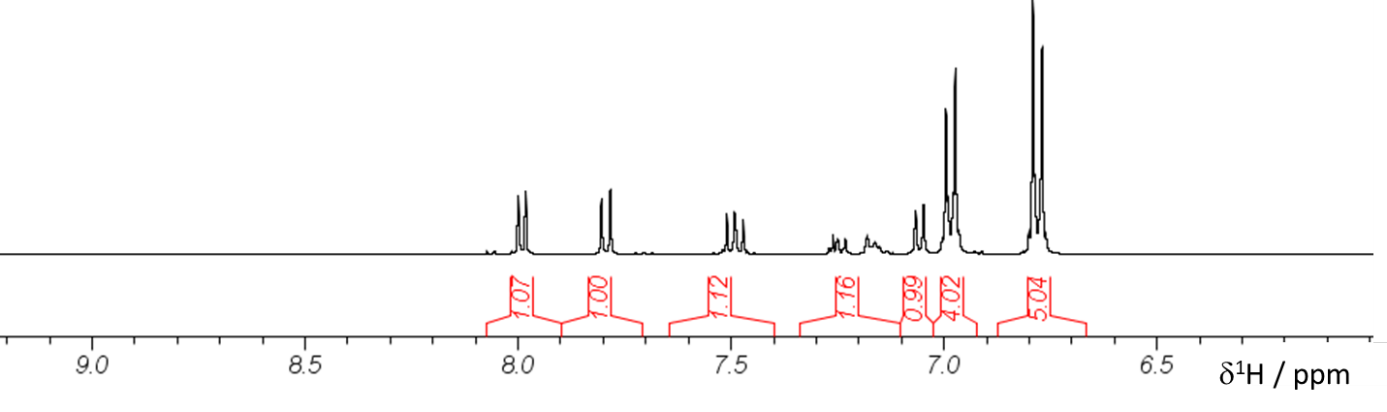

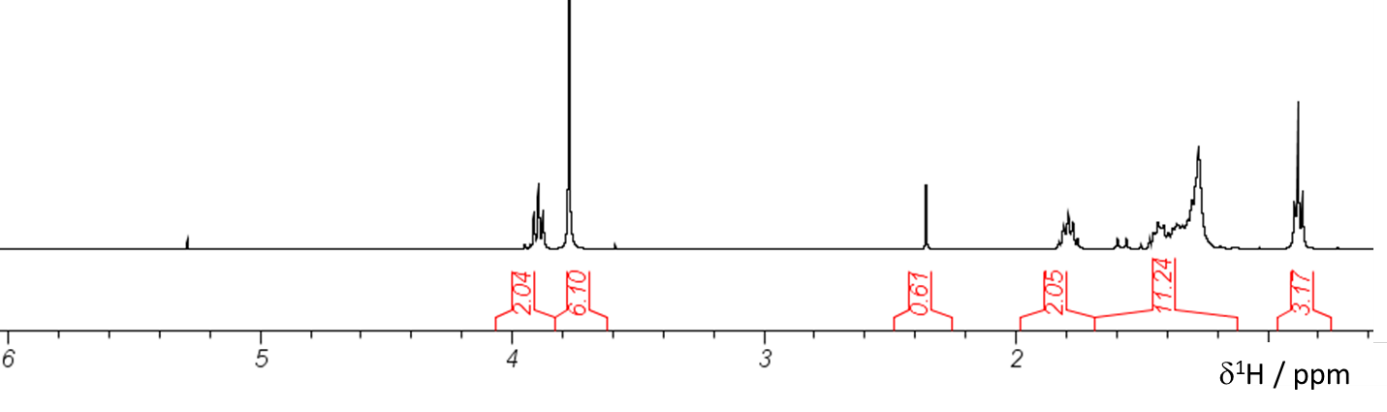


^1^H NMR spectrum of 3 (CDCl_3_, 298K).


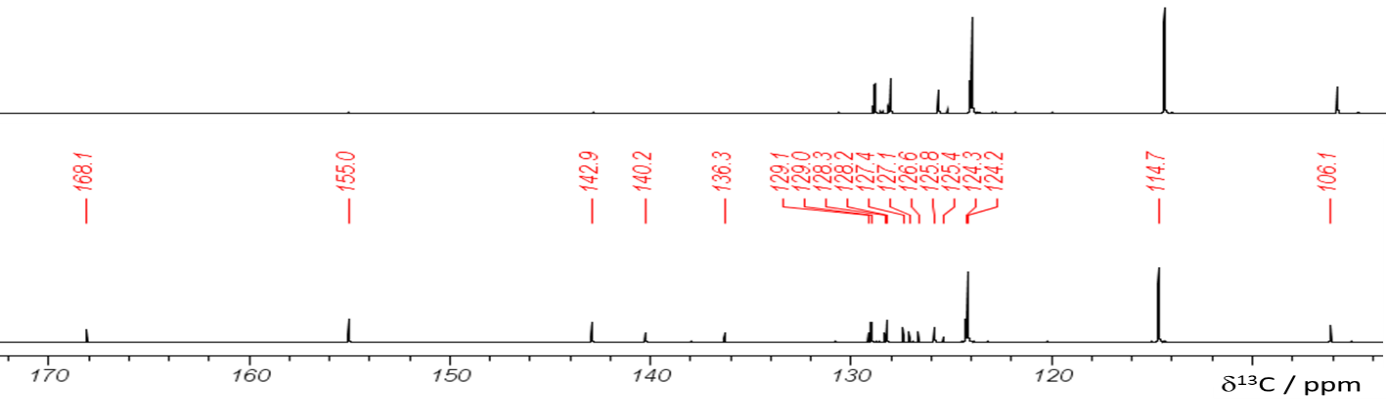


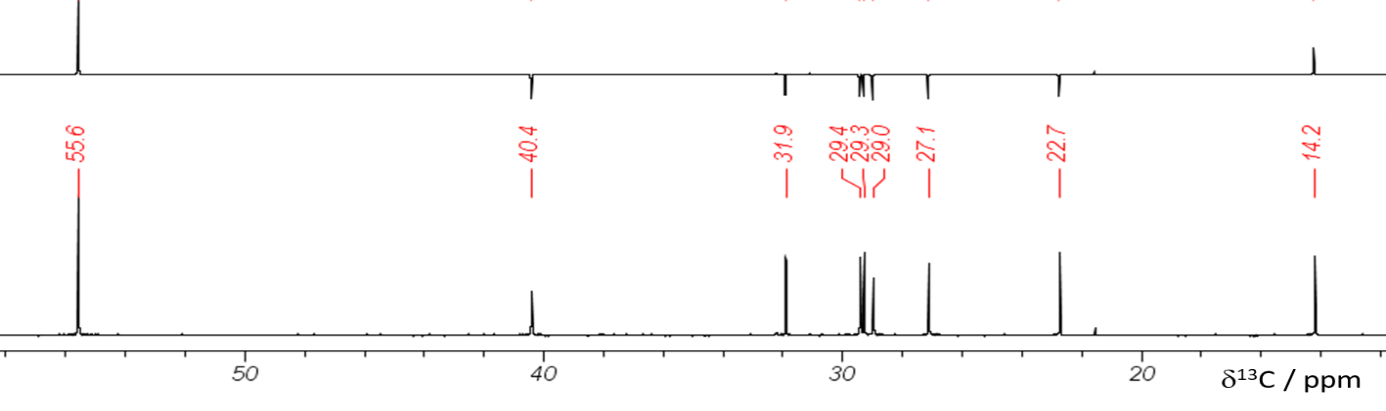


DEPT-135 (top) and ^13^C (bottom) NMR spectra of 3 (CDCl_3_, 298K).


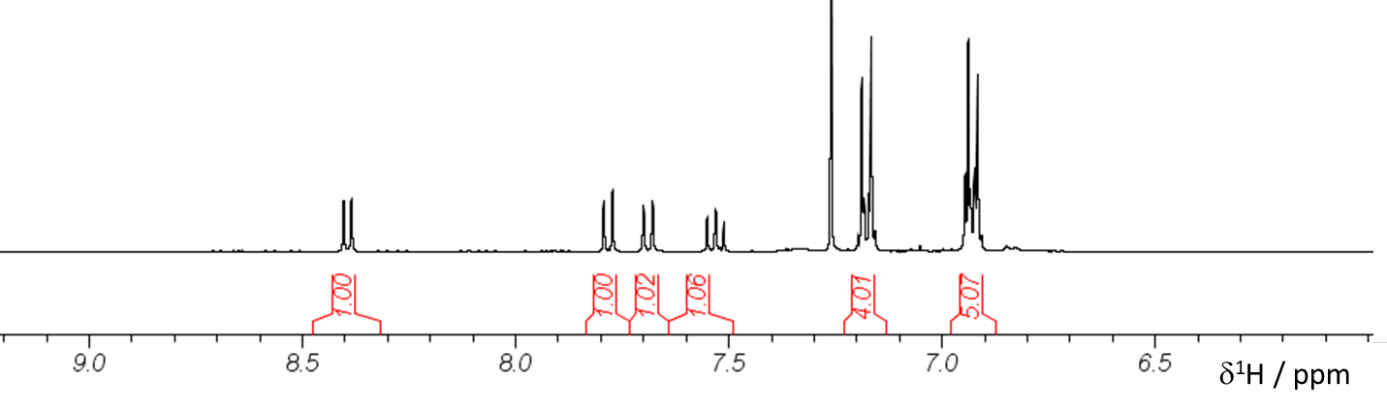


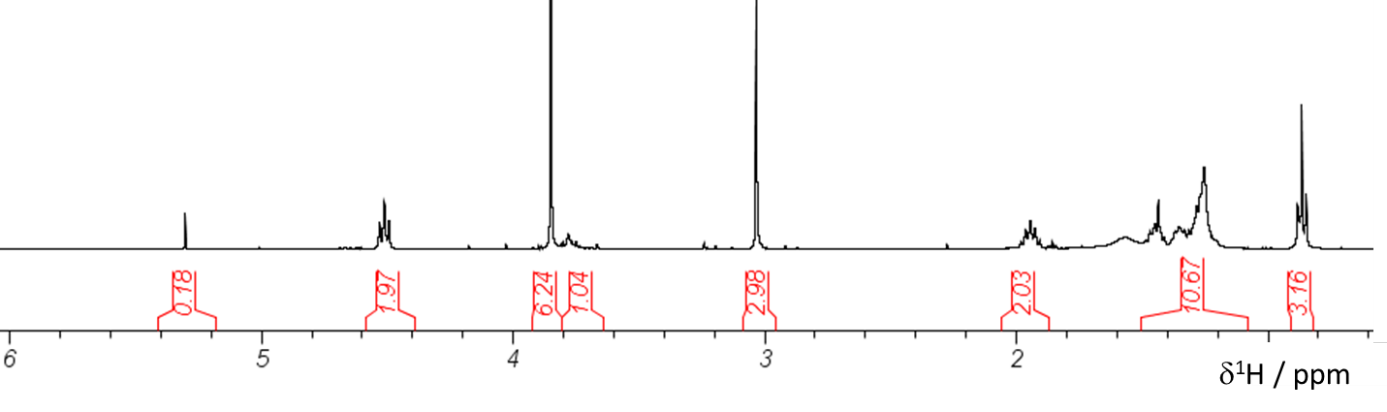


^1^H NMR spectrum of 4 (CDCl_3_, 298K).

**
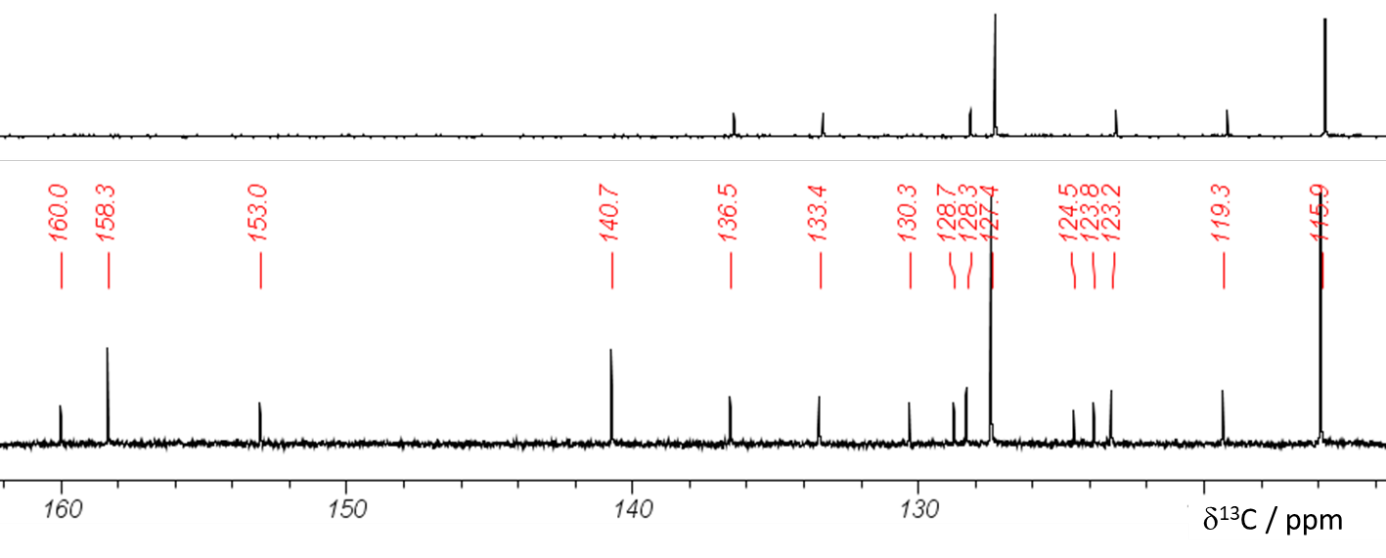
**


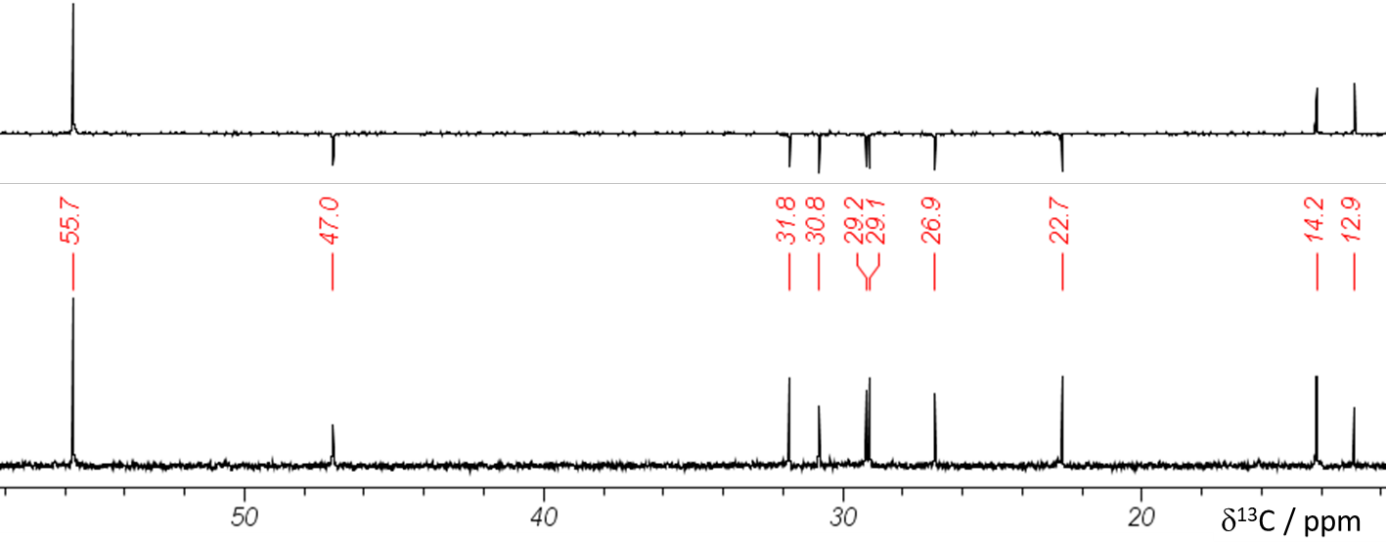


DEPT-135 (top) and ^13^C (bottom) NMR spectra of 4 (CDCl_3_, 298K).


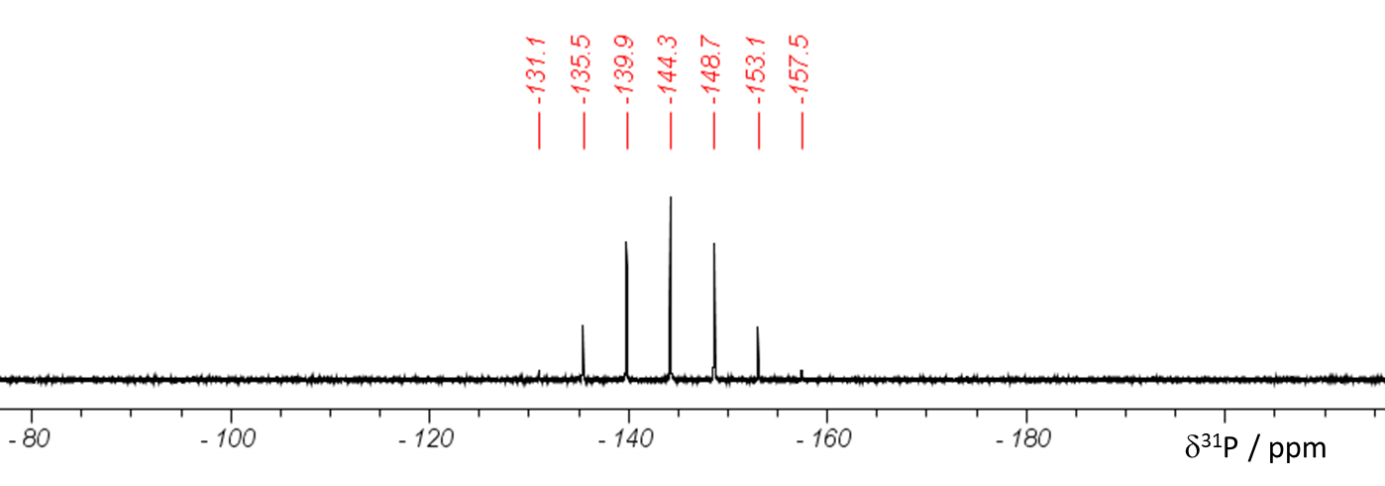


^31^P NMR spectrum of 4 (CDCl_3_, 298K).


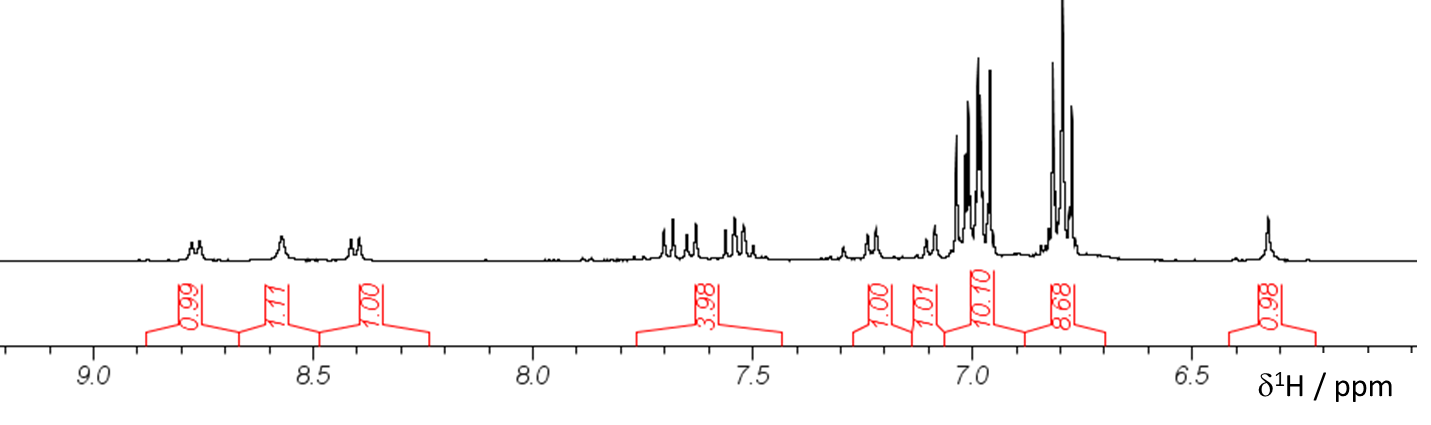


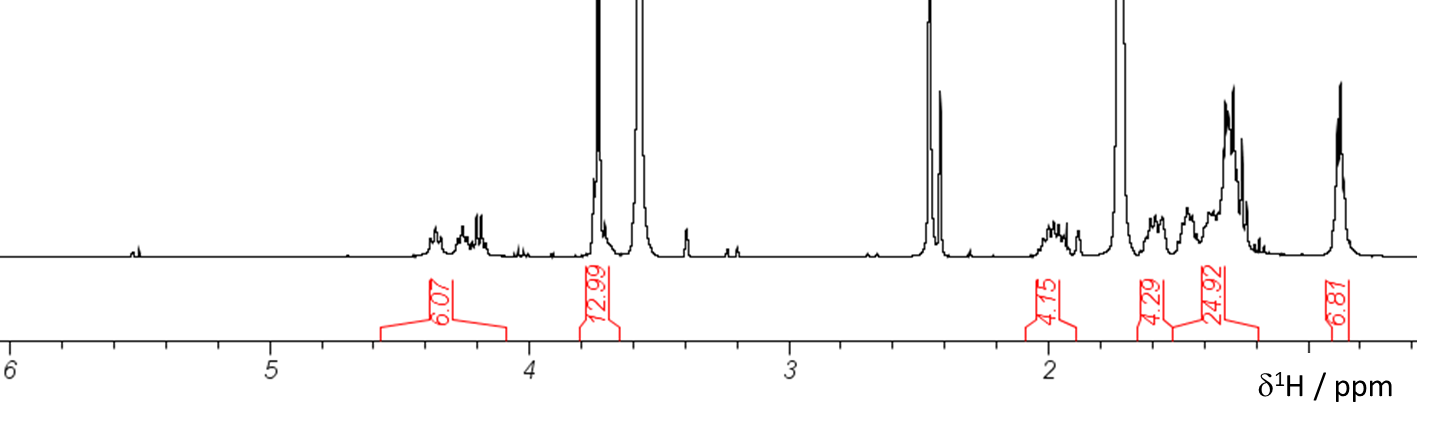


^1^H NMR spectrum of RSQ2 (THF-d_8_, 298K).


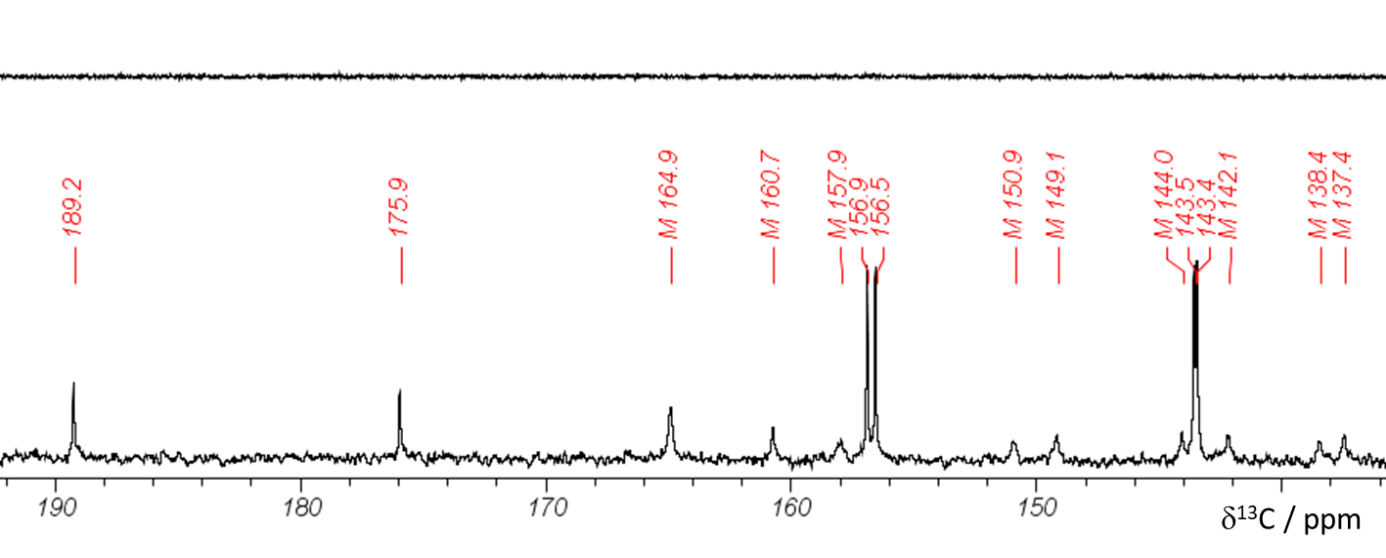


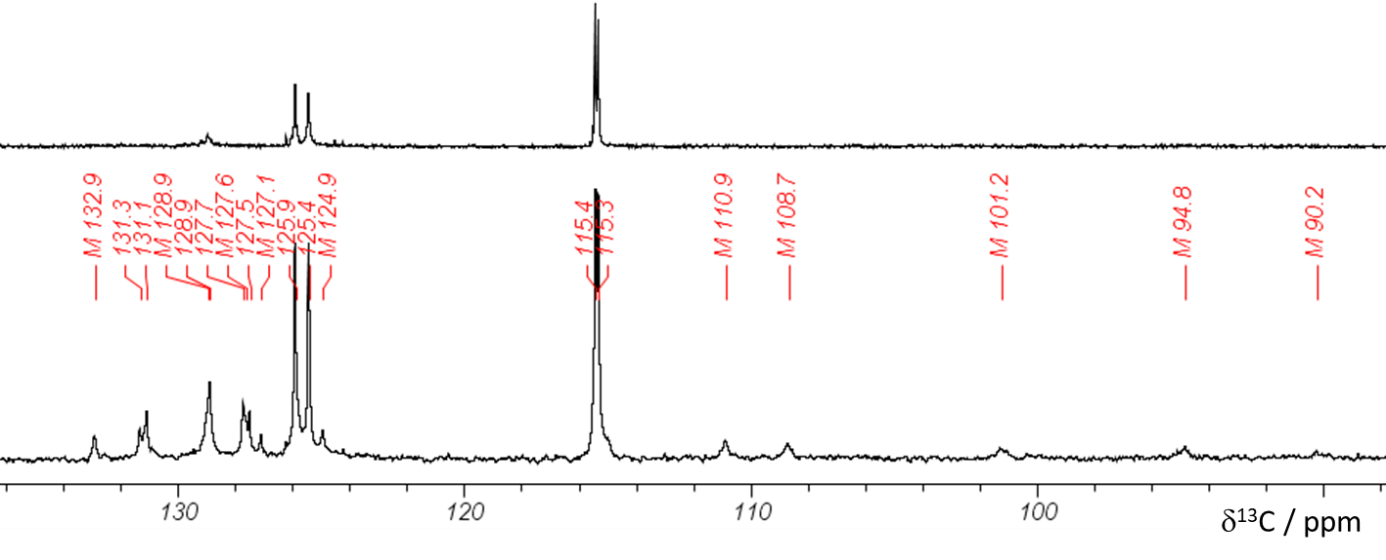


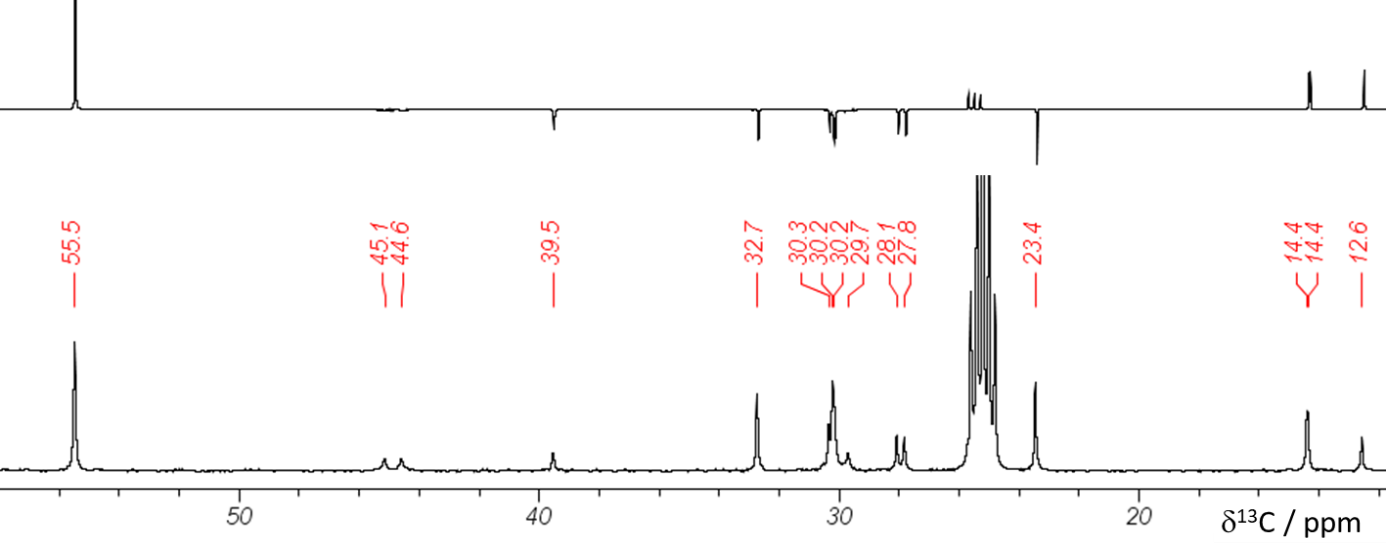


DEPT-135 (top) and ^13^C (bottom) NMR spectra of RSQ2 (THF-d_8_, 298K).

**
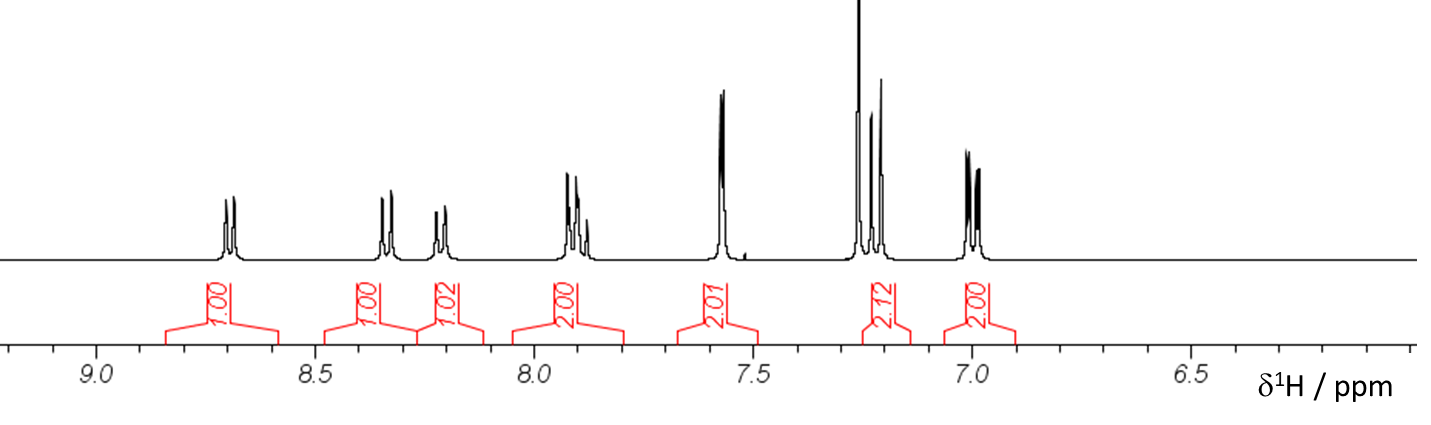
**

**
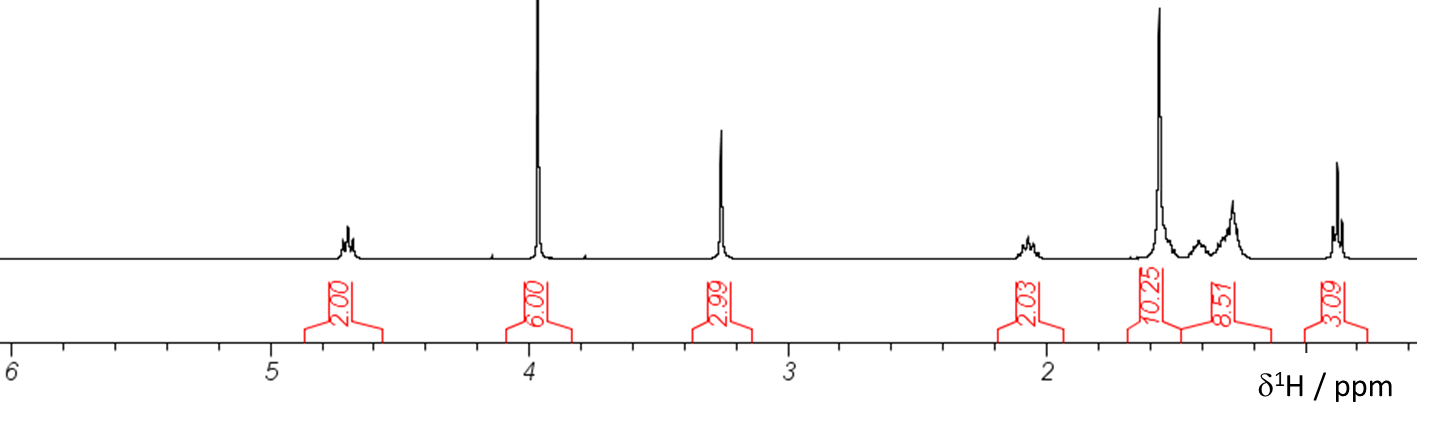
**

^1^H NMR spectrum of 6 (CDCl_3_, 298K).


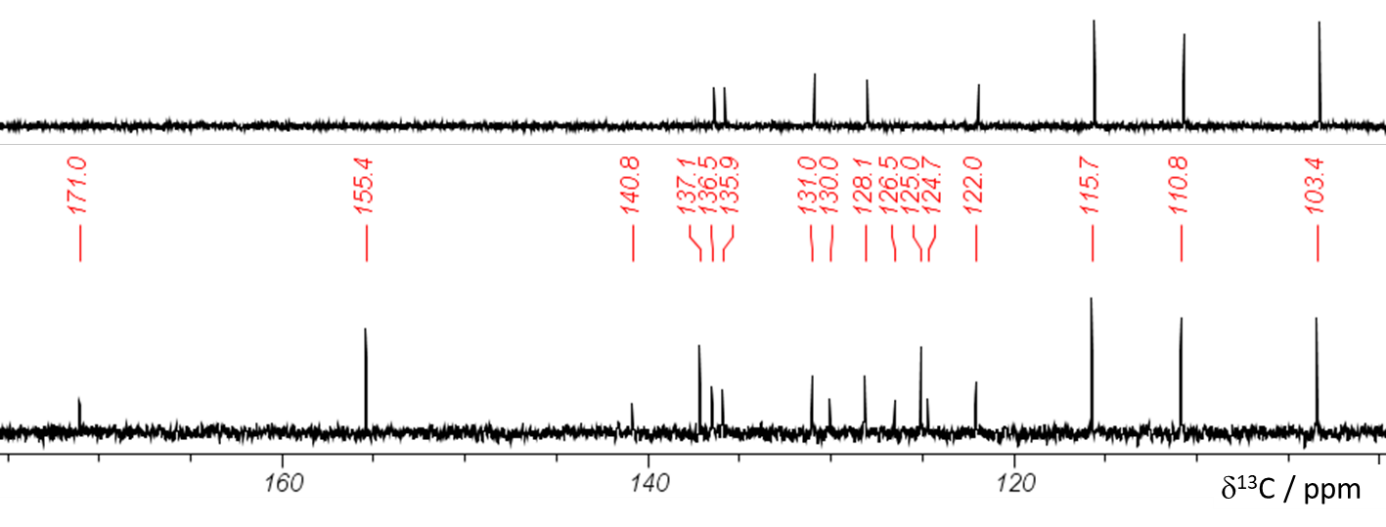


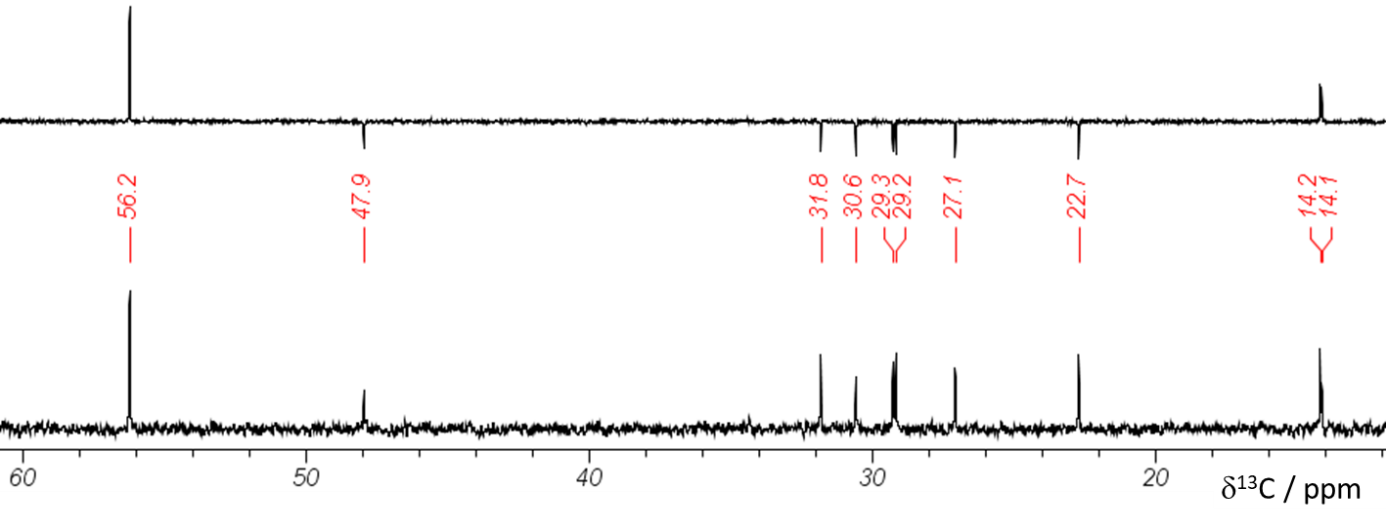


DEPT-135 (top) and ^13^C (bottom) NMR spectra of 6 (CDCl_3_, 298K).


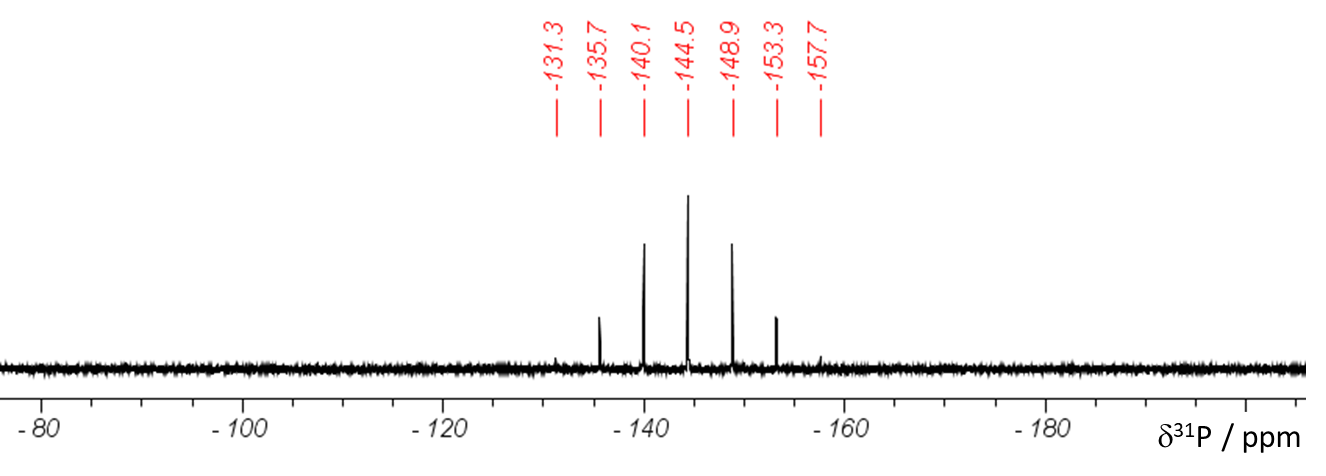


^31^P NMR spectrum of 6 (CDCl_3_, 298K).


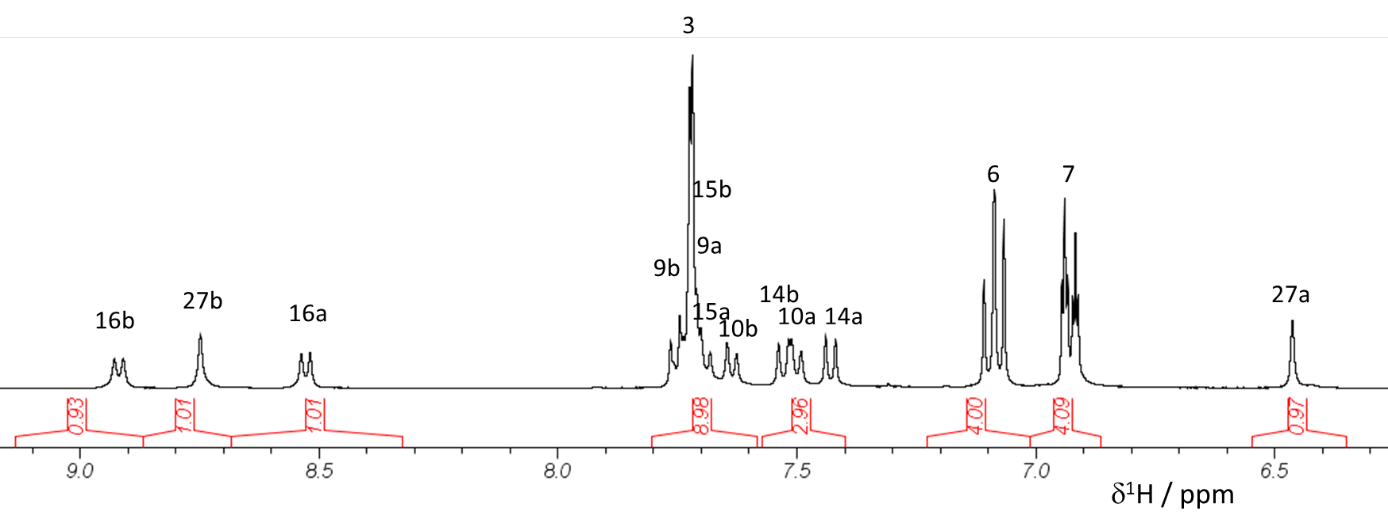


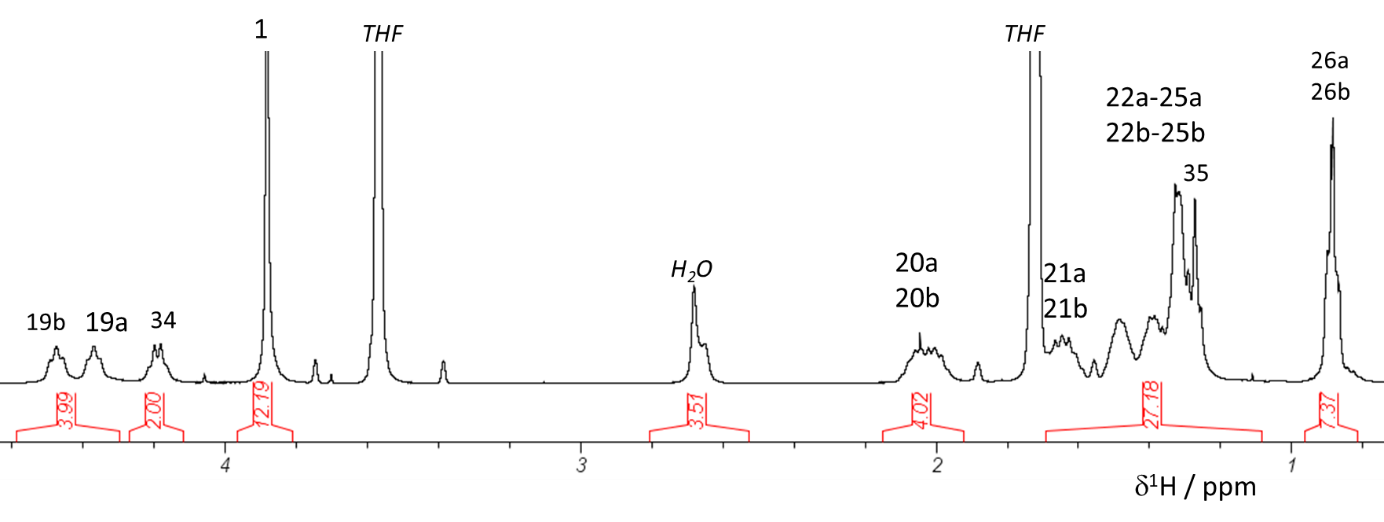


^1^H NMR spectrum of RSQ3 with assignment of resonances (d_8_-THF, 258K).


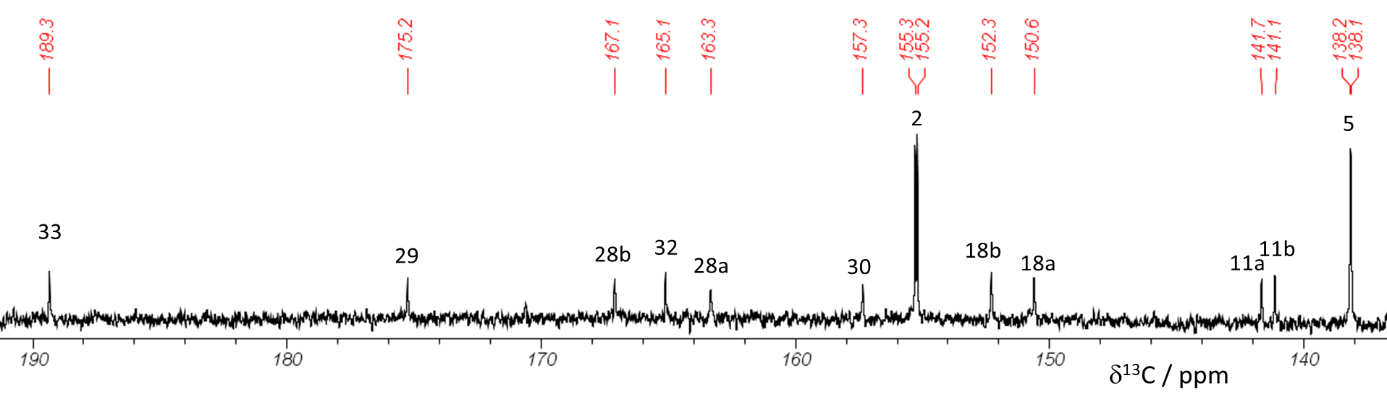


^
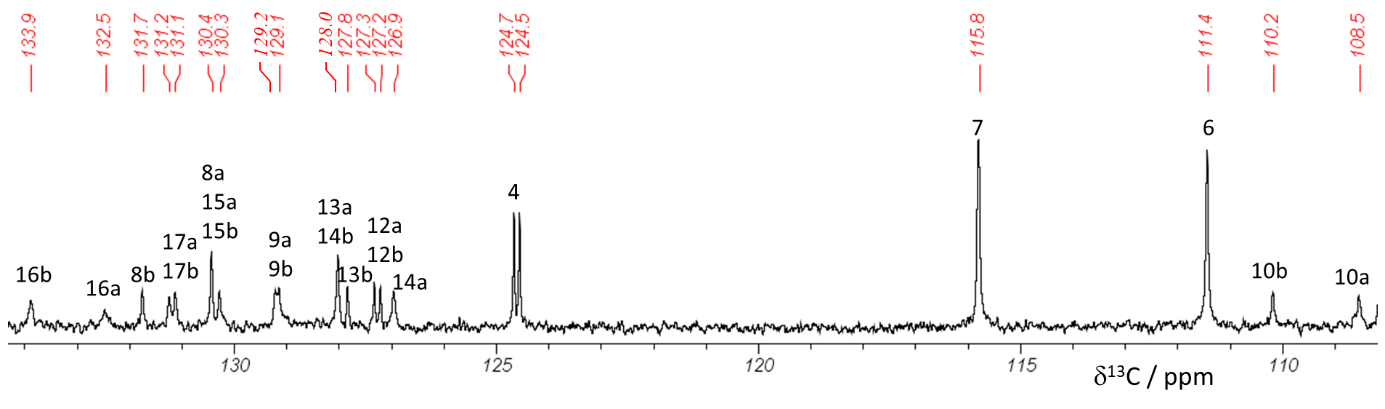
^

^
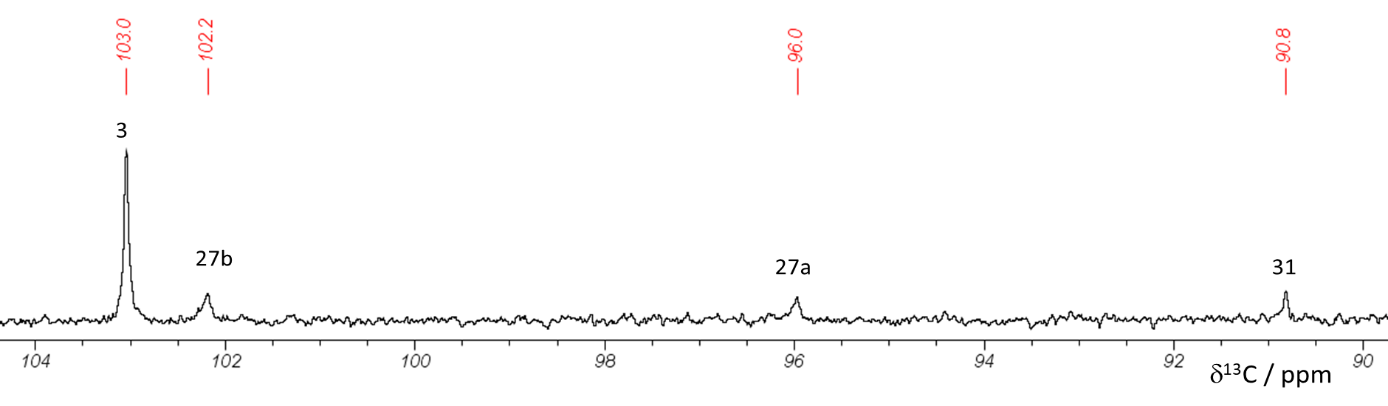
^


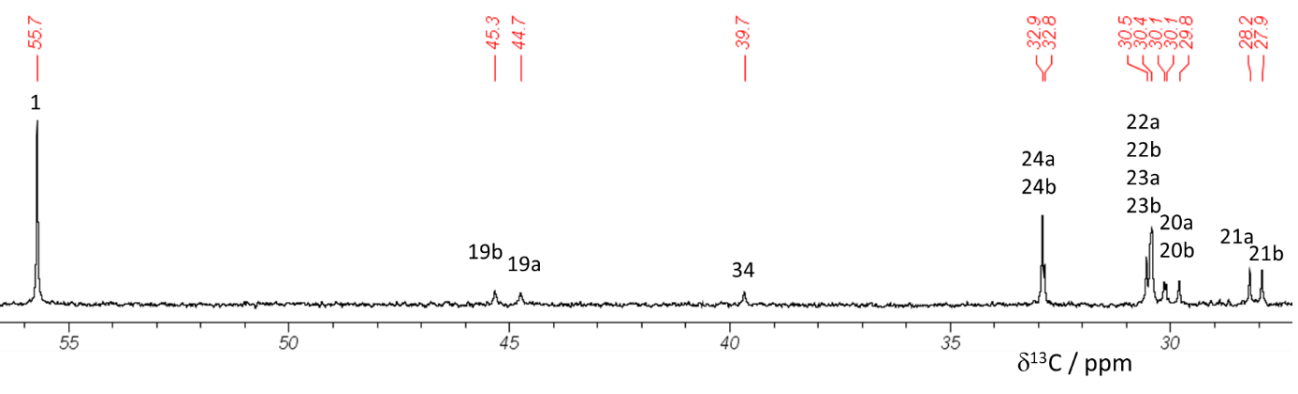


^
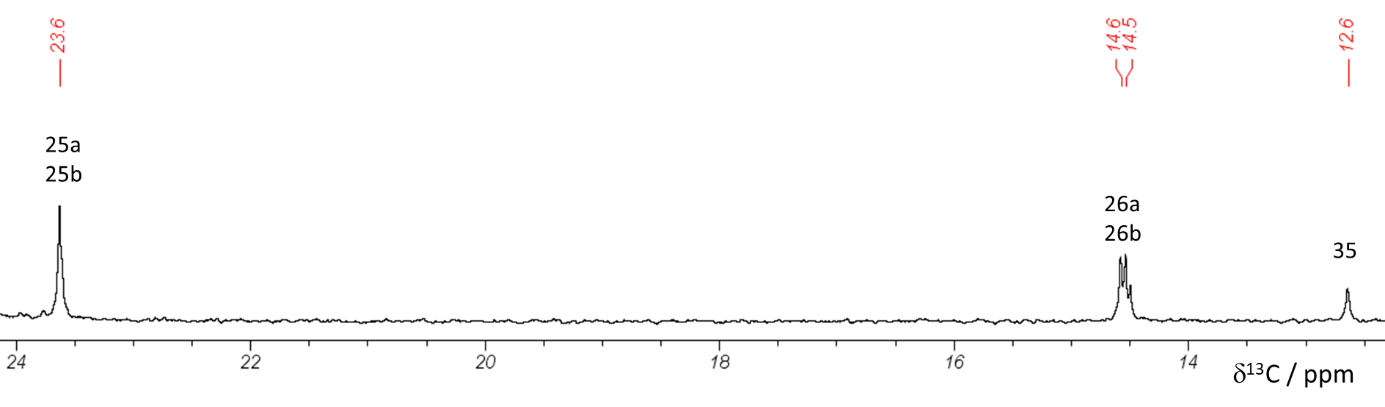
^

^13^C NMR spectrum of RSQ3 with assignment of resonances (d_8_-THF, 258K).


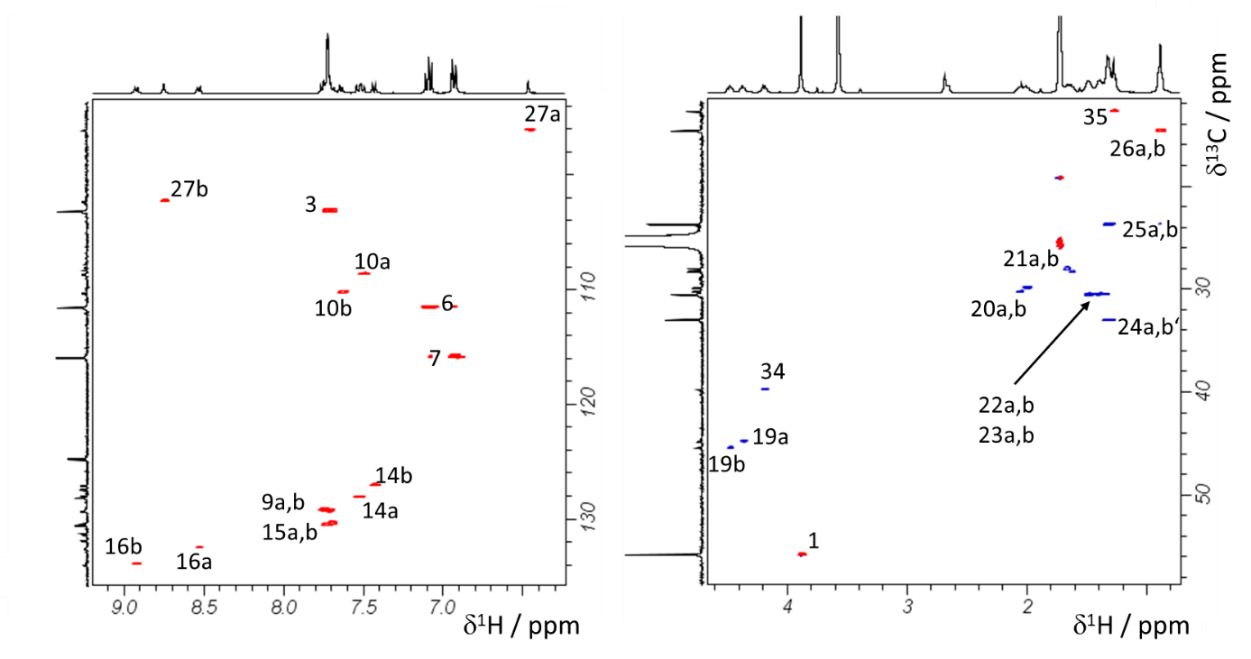


^1^H-^13^C HSQC NMR spectrum of RSQ3 with assignment of resonances (d_8_-THF, 258K).


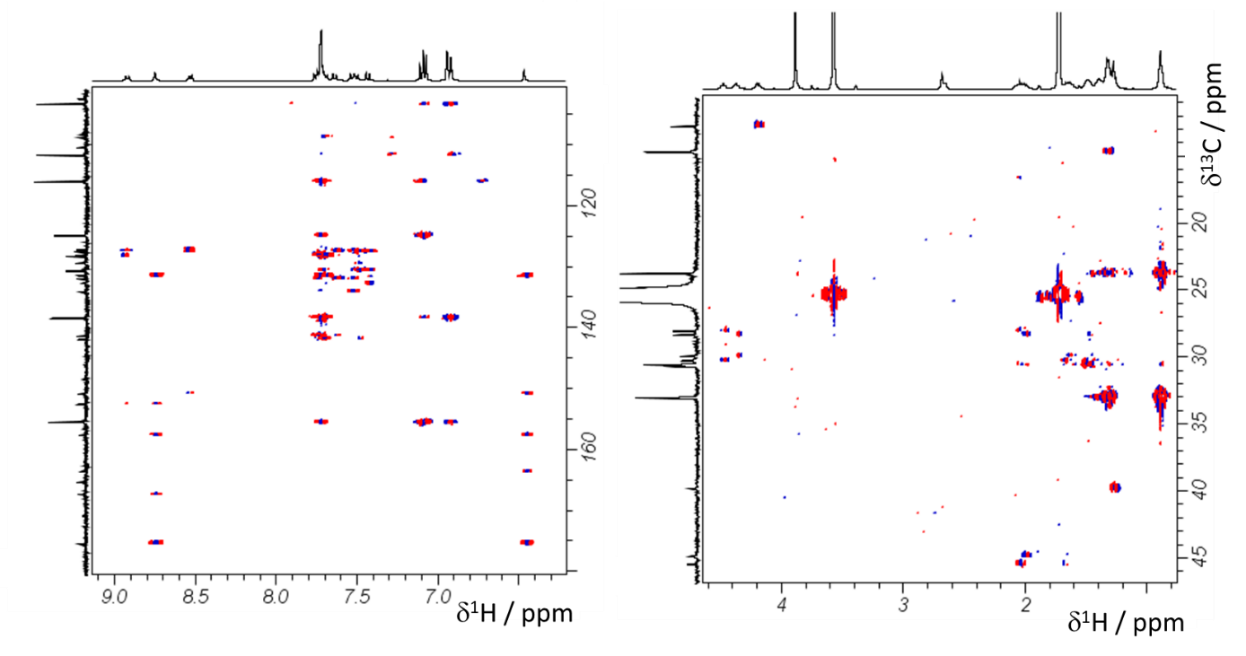


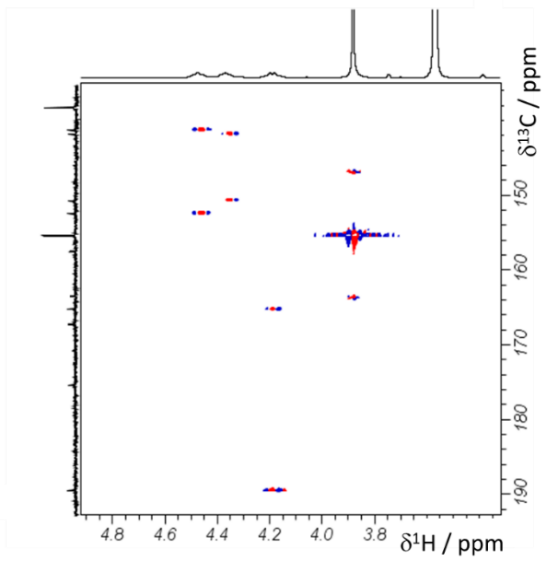


^1^H-^13^C HMBC NMR spectrum of RSQ3 (d_8_-THF, 258K).


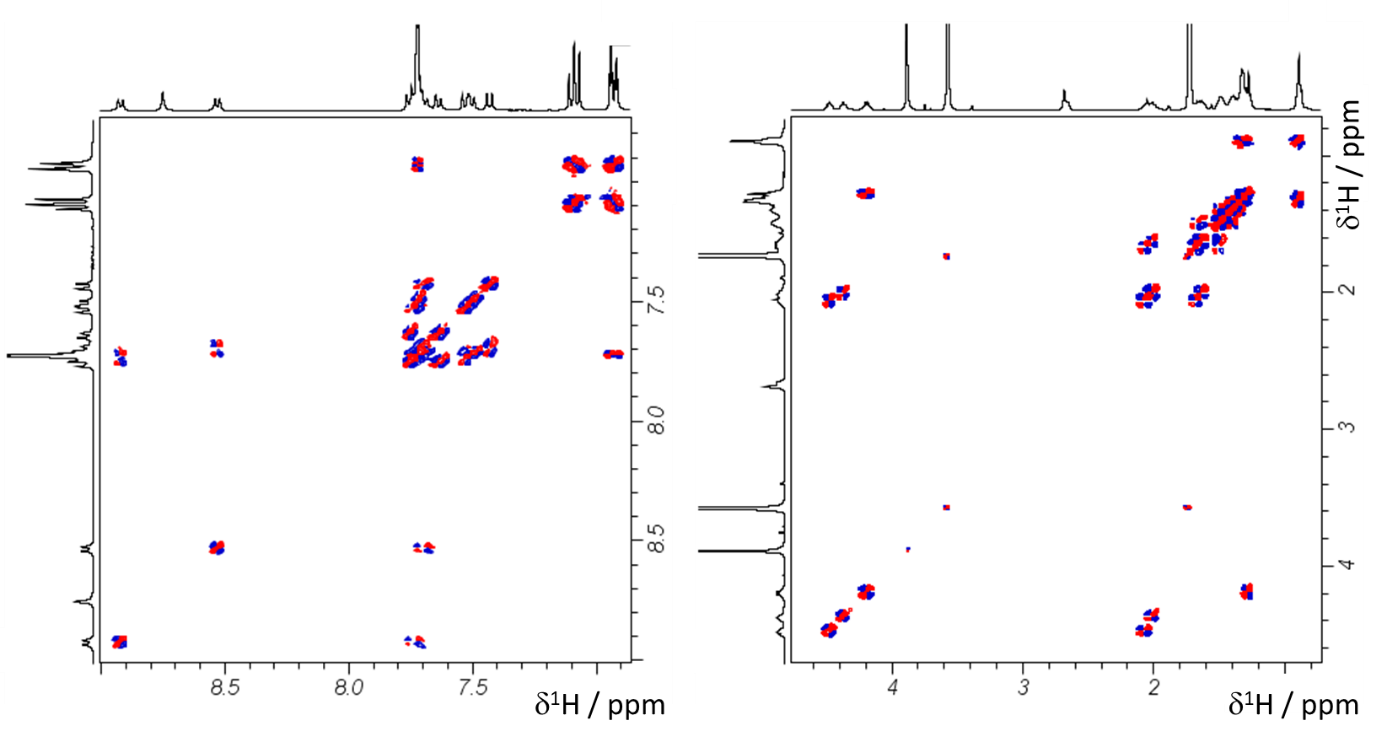


^1^H-^1^H DQF-COSY NMR spectrum of RSQ3 (d_8_-THF, 258K).


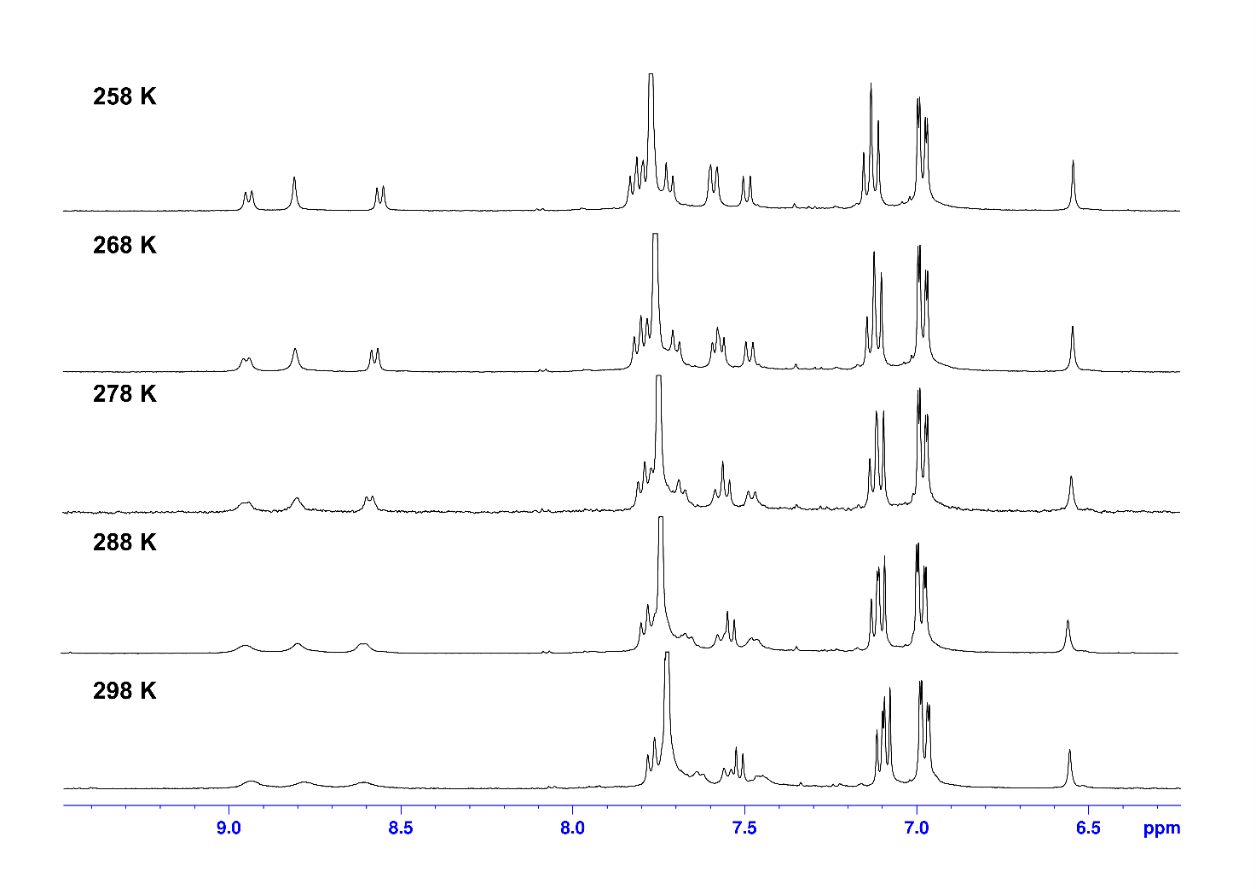


Temperature dependence of ^1^H NMR spectra of RSQ3 between 258K and 298 K. Line narrowing with decreasing temperature is most pronounced for hydrogens at positions 16a, 16b and 27b, which interact with the oxygens of the acceptor unit. Similar line broadening was observed for the most de-shielded aromatic protons and the corresponding olefinic protons in the DCRSQ dye family as well, measured in CDCl_3_ at r.t.


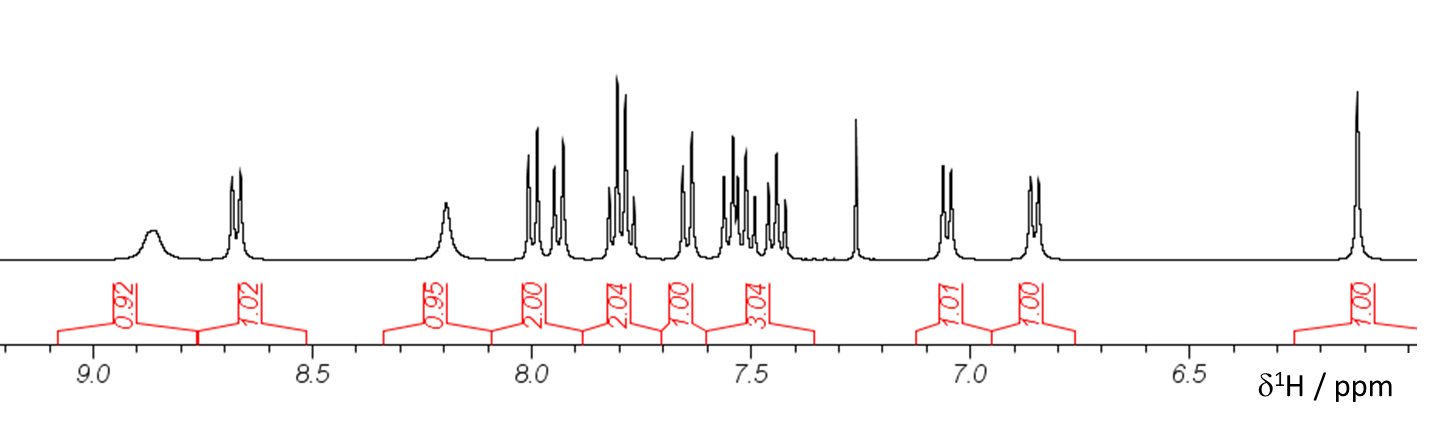


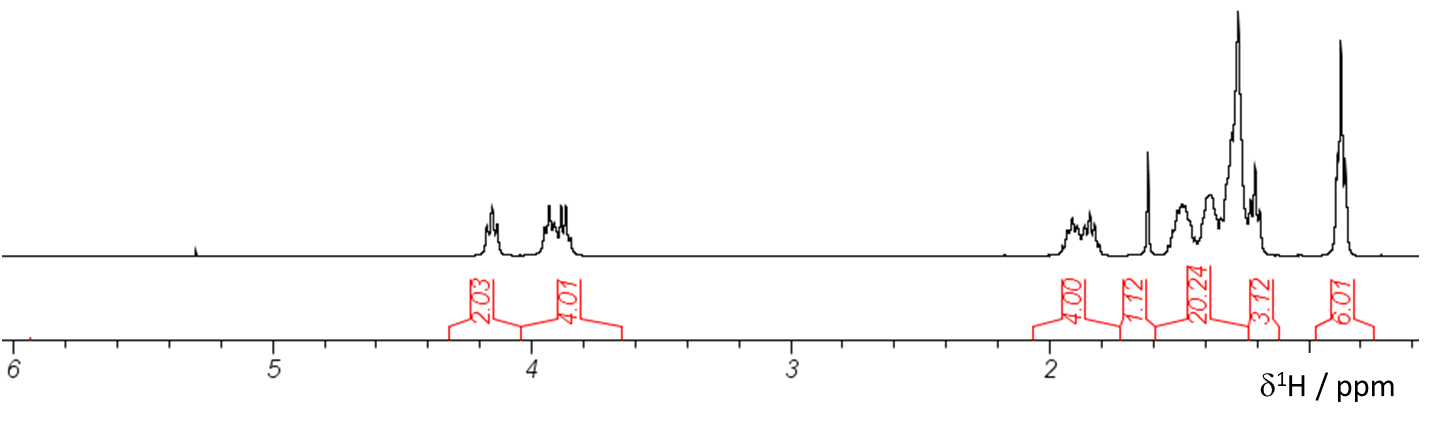


^1^H NMR spectrum of DCRSQ1 (CDCl_3_, 298K).


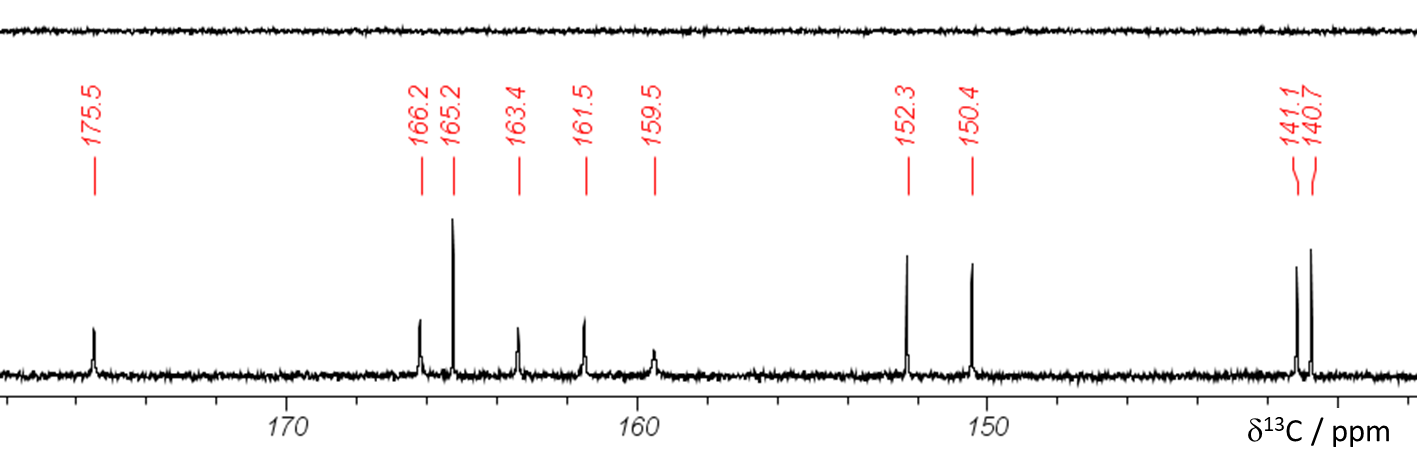


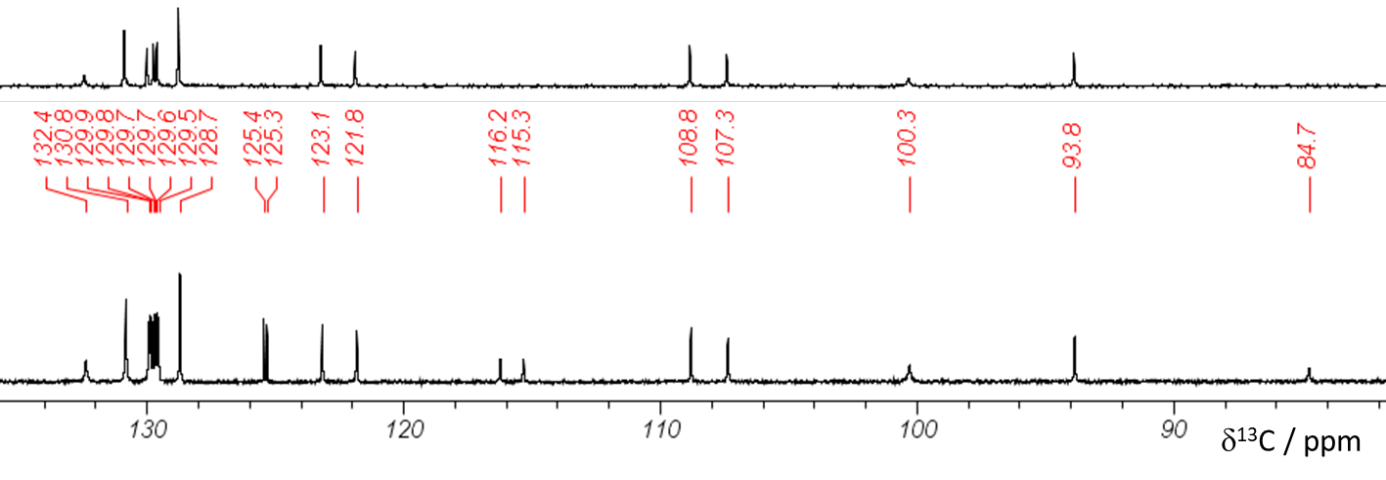


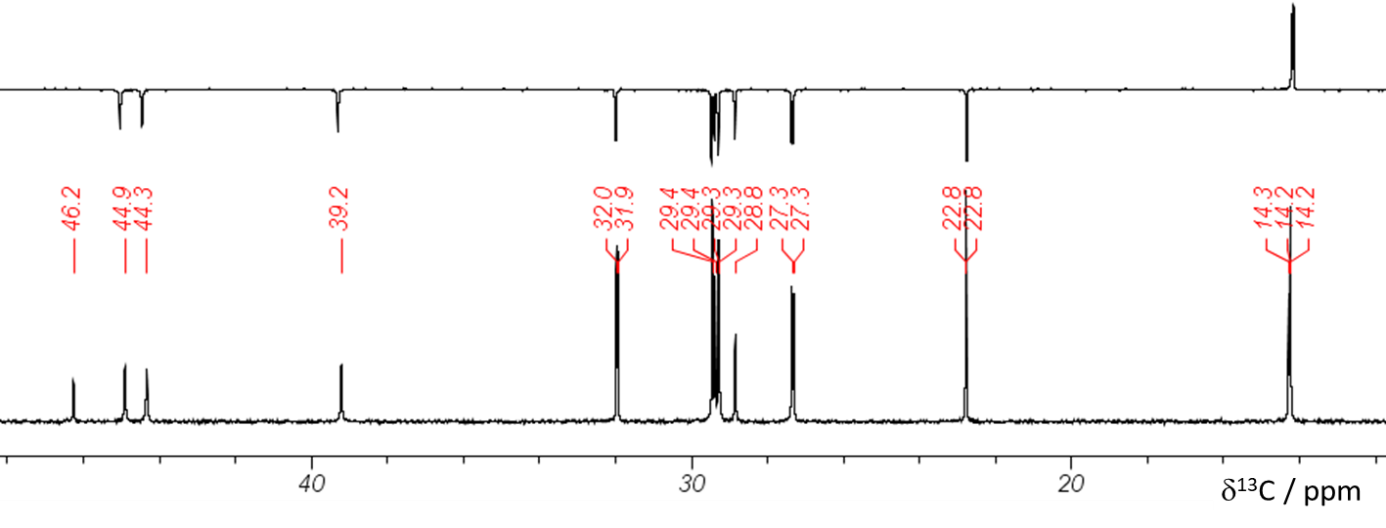


DEPT-135 (top) and ^13^C (bottom) NMR spectra of DCRSQ1 (CDCl_3_, 298K).

**
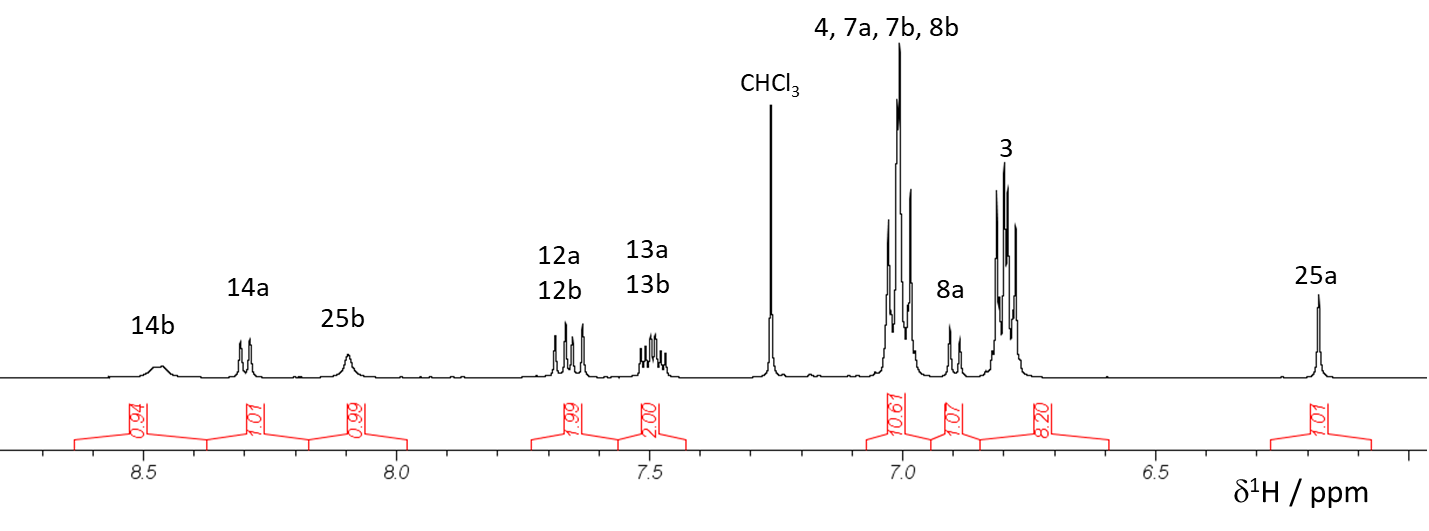
**


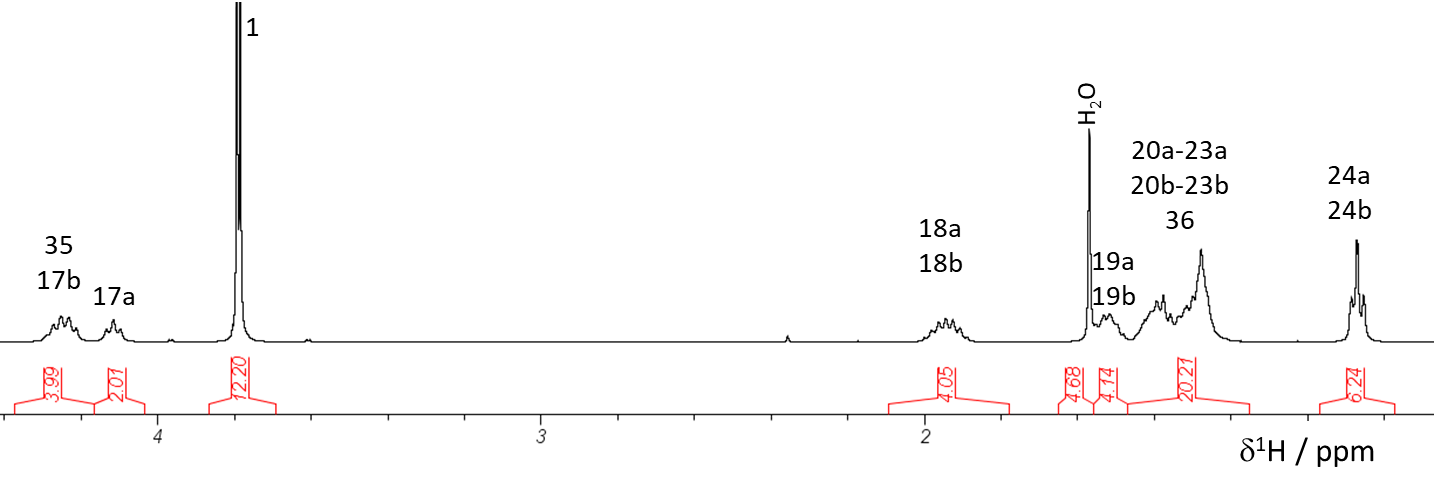


^1^H NMR spectrum of DCRSQ2 with assignment of resonances (CDCl_3_, 298K).


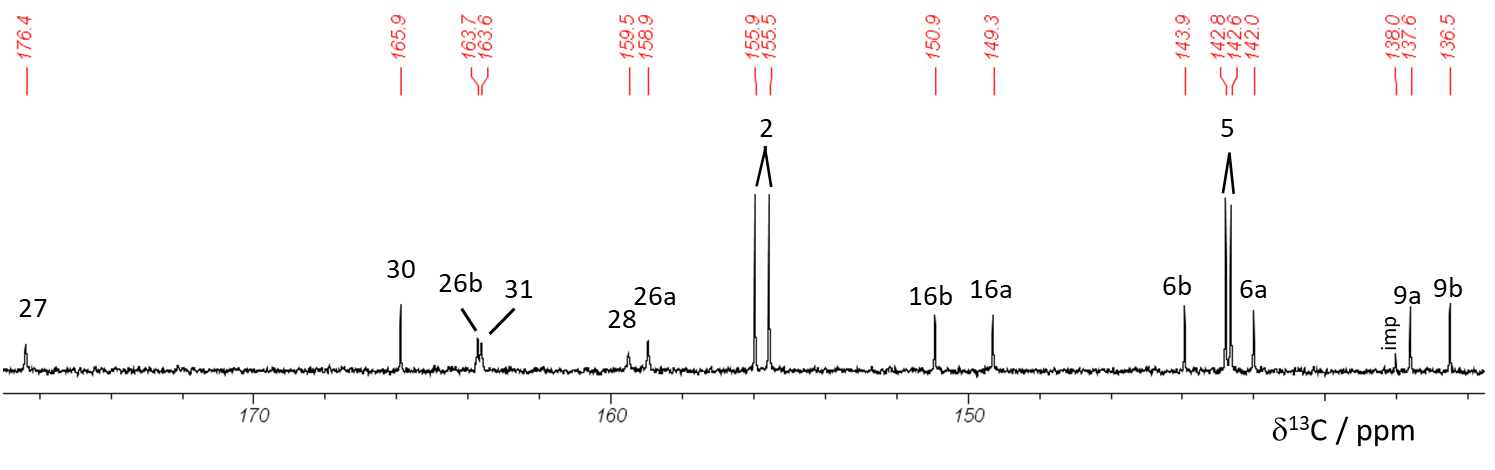


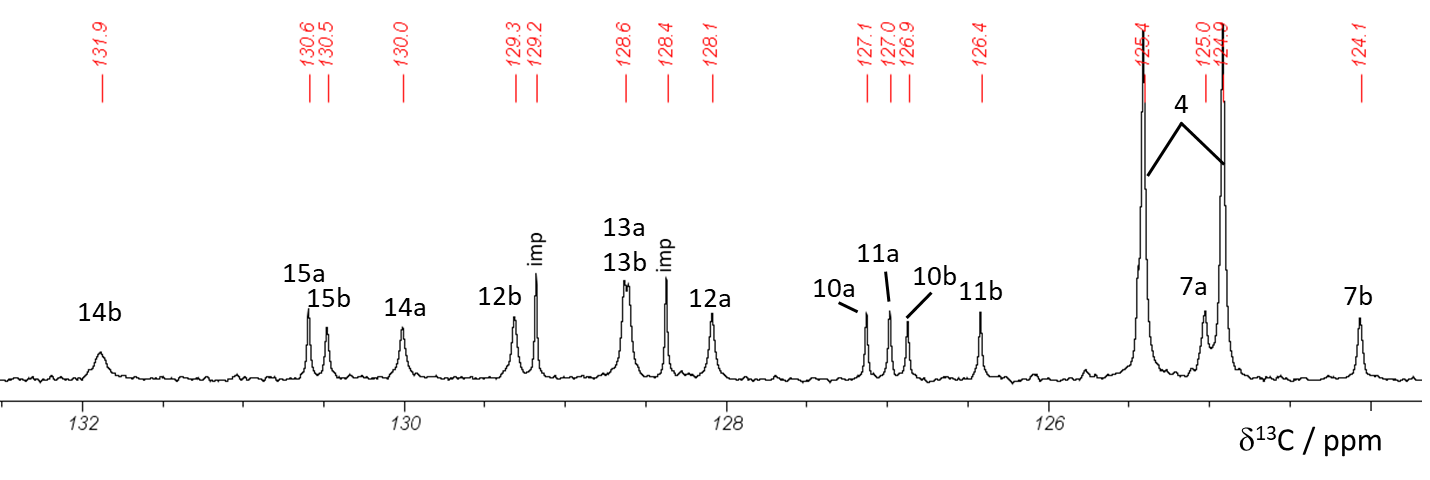


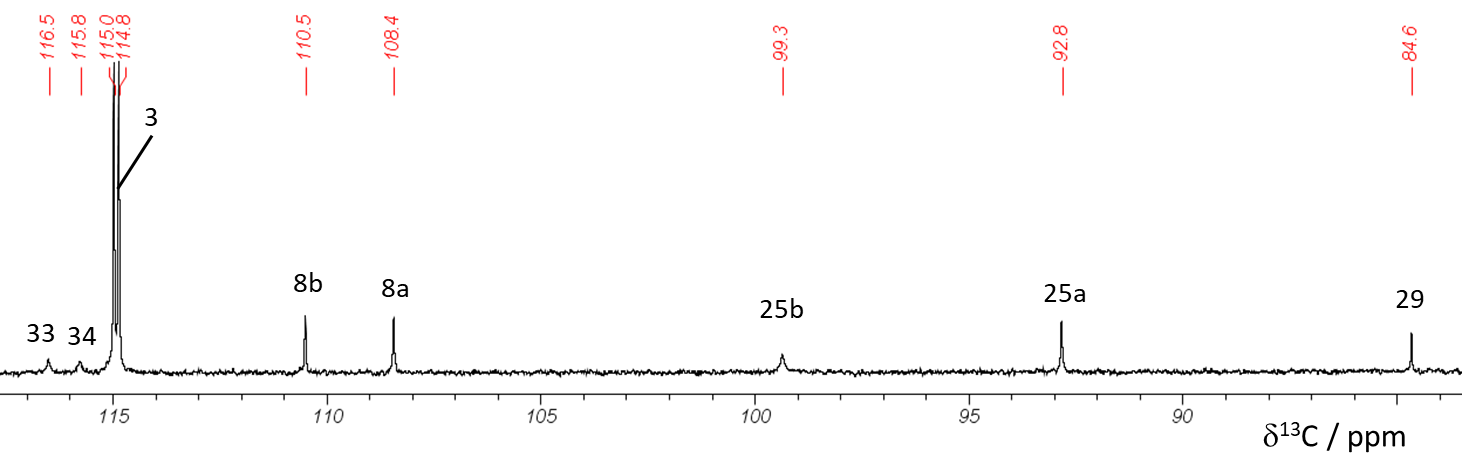


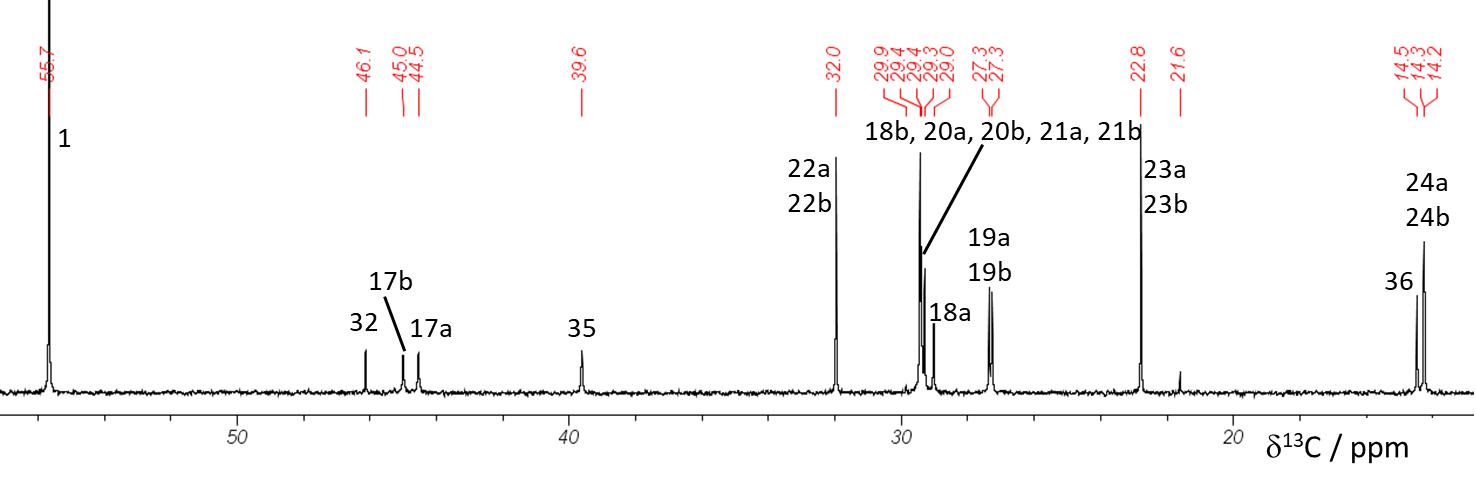


^13^C NMR spectrum of DCRSQ2 with assignment of resonances (CDCl_3_, 298K).


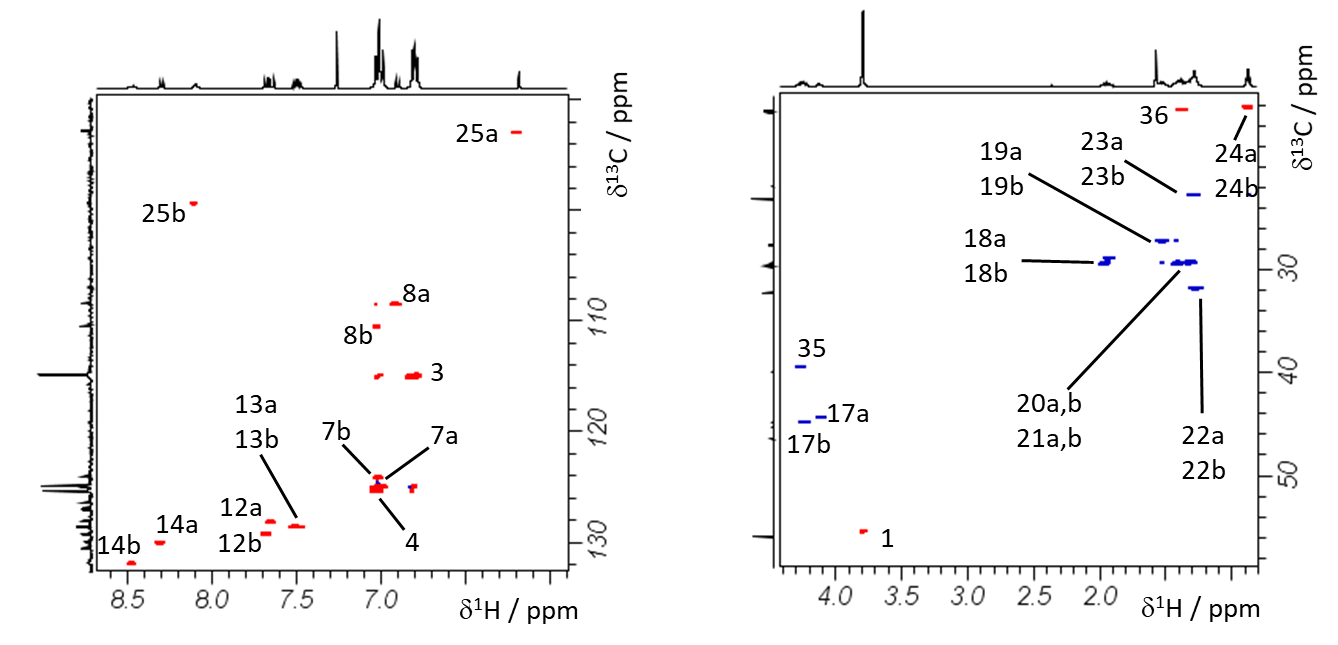


^1^H-^13^C HSQC NMR spectrum of **DCRSQ2** with assignment of resonances (CDCl_3_, 298K).


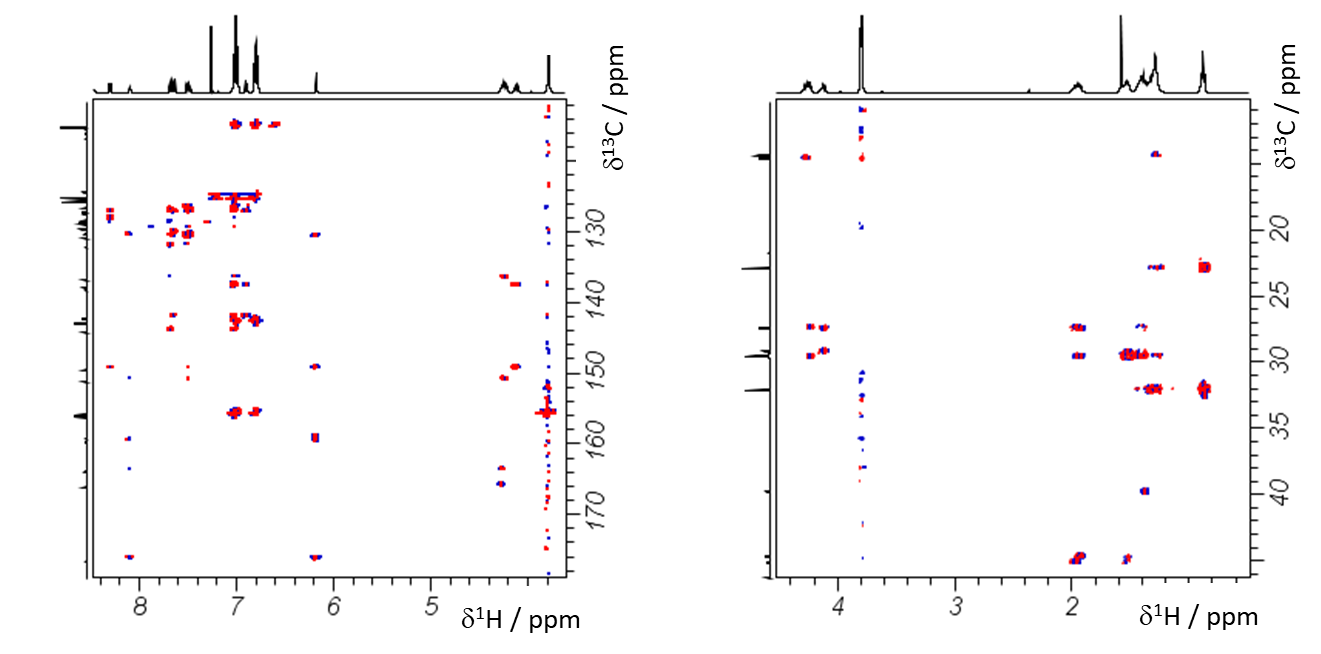


^1^H-^13^C HMBC NMR spectrum of **DCRSQ2** (CDCl_3_, 298K).


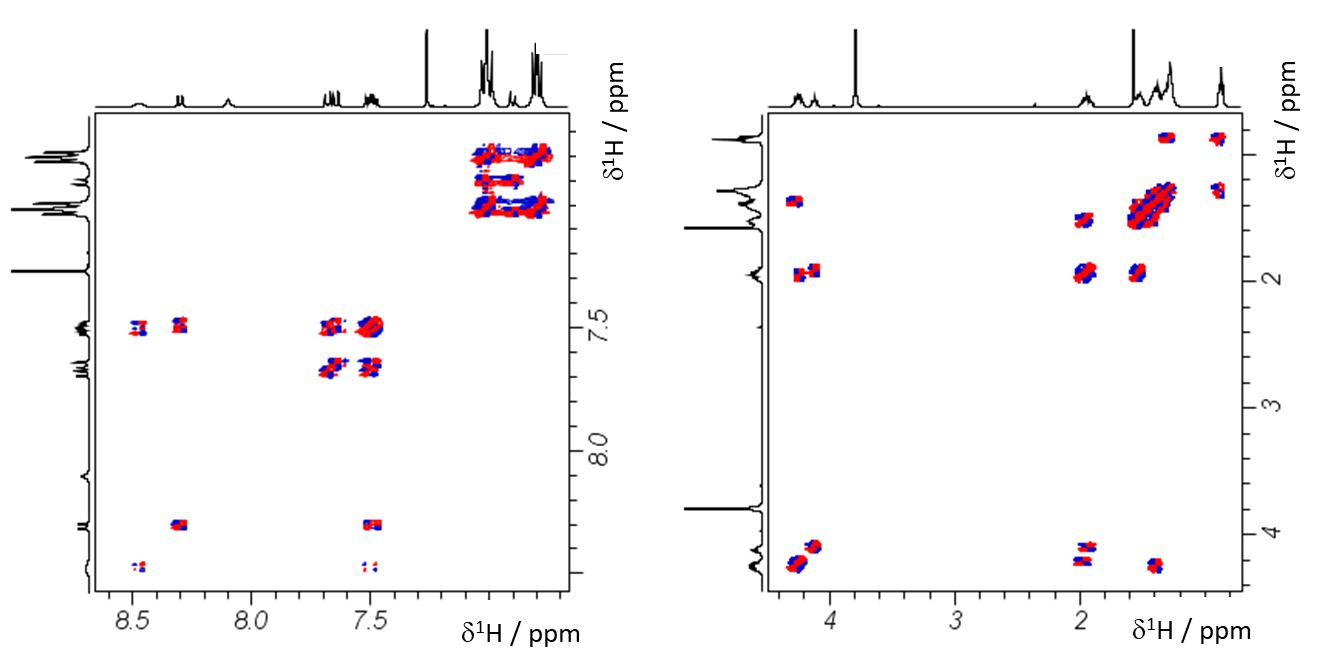


^1^H-^1^H DQF-COSY NMR spectrum of **DCRSQ2** (CDCl_3_, 298K**)**.


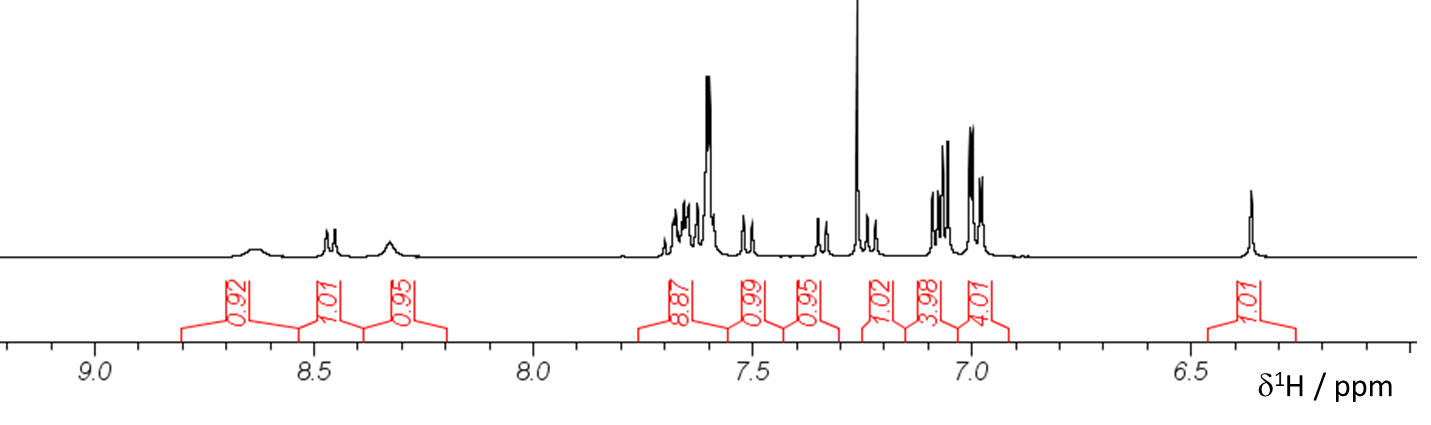


**
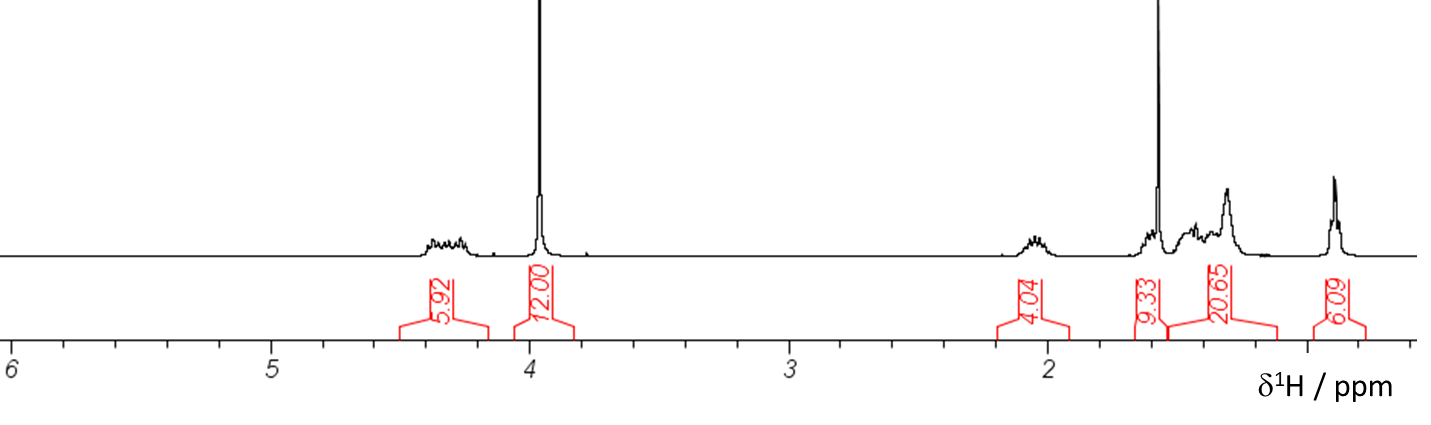
**

^1^H NMR spectrum of DCRSQ3 in (CDCl_3_, 298K).


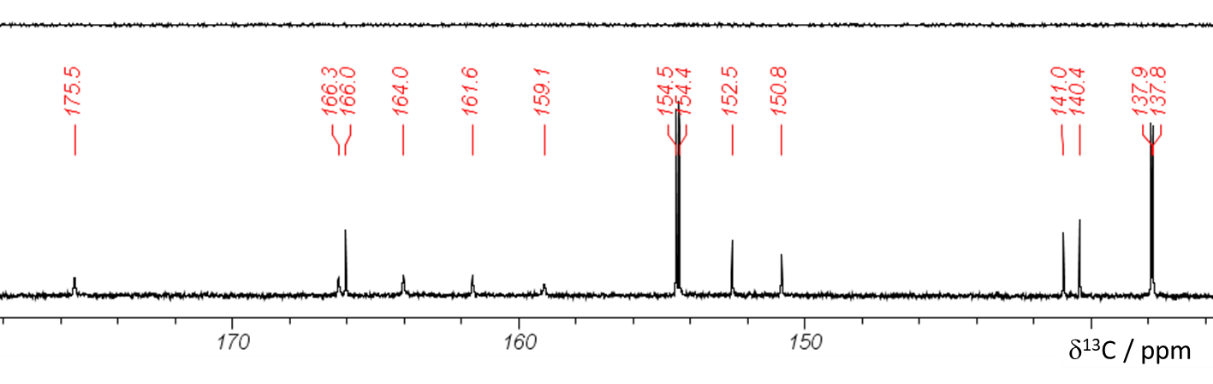


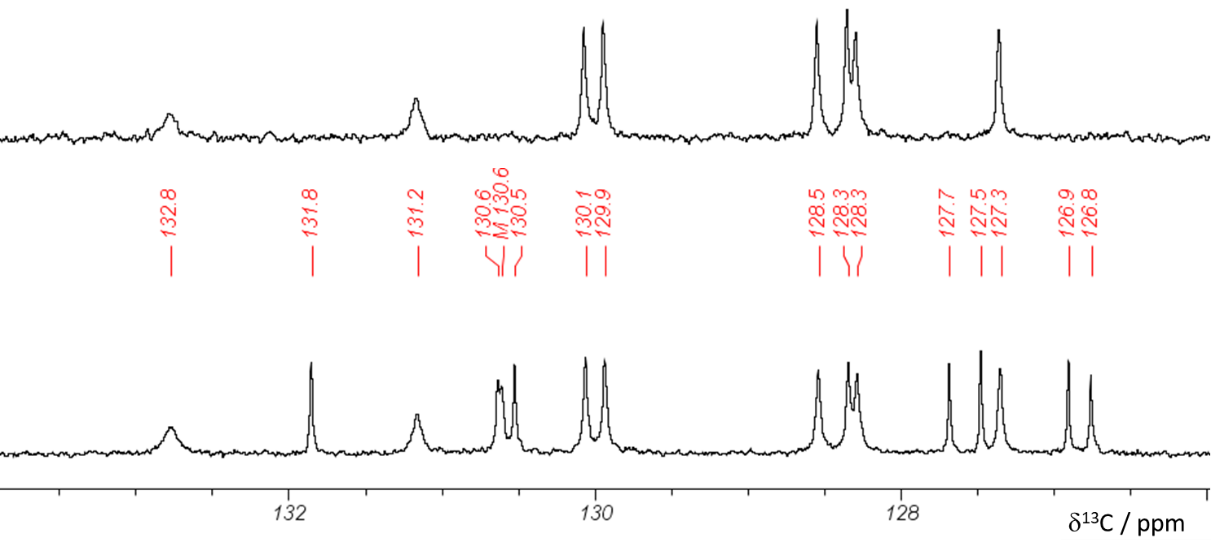


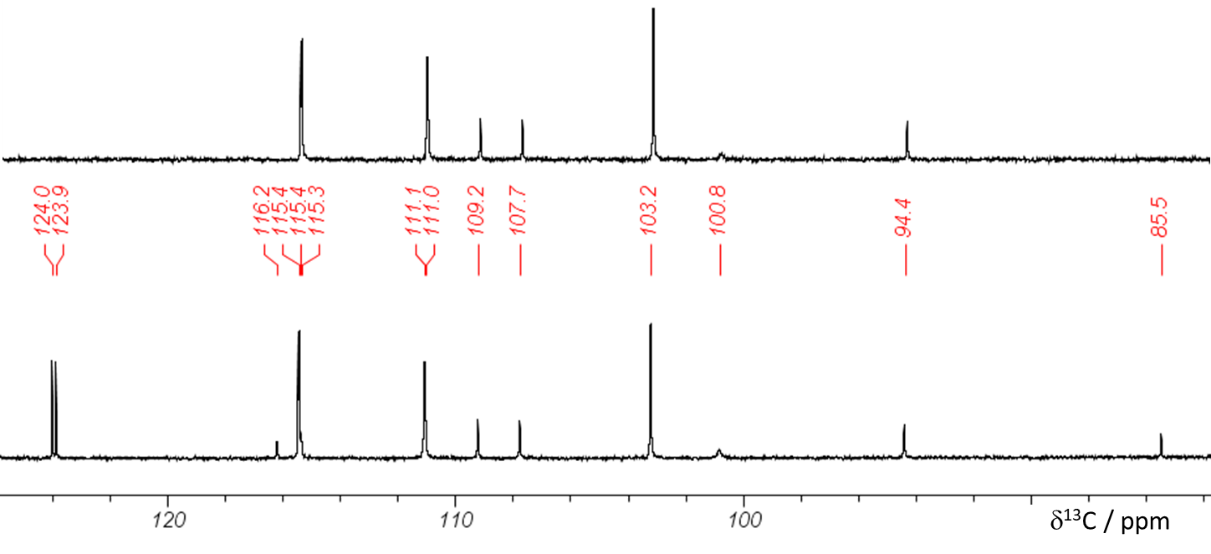


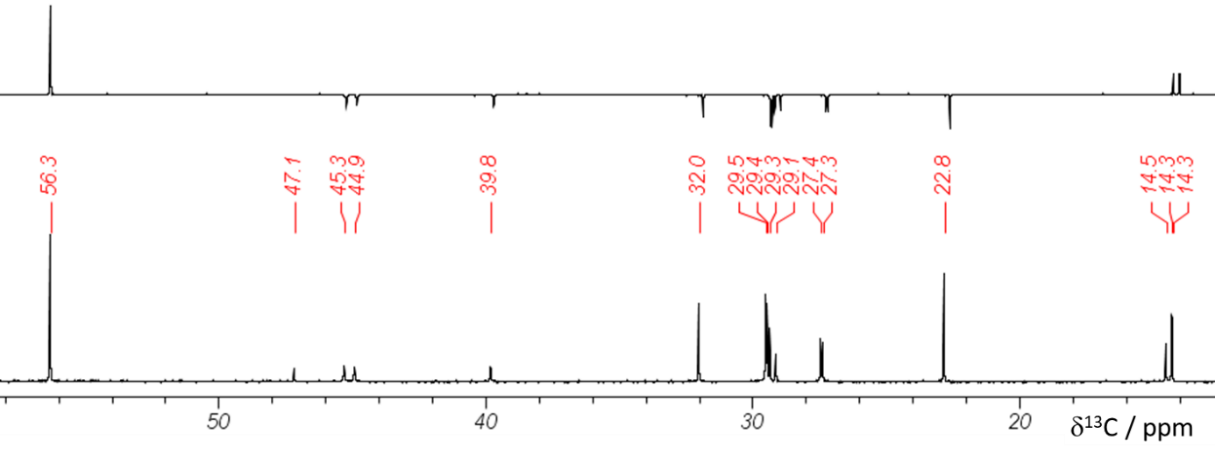


DEPT-135 (top) and ^13^C (bottom) NMR spectra of DCRSQ3 (CDCl_3_, 298K).

# MS Spectra


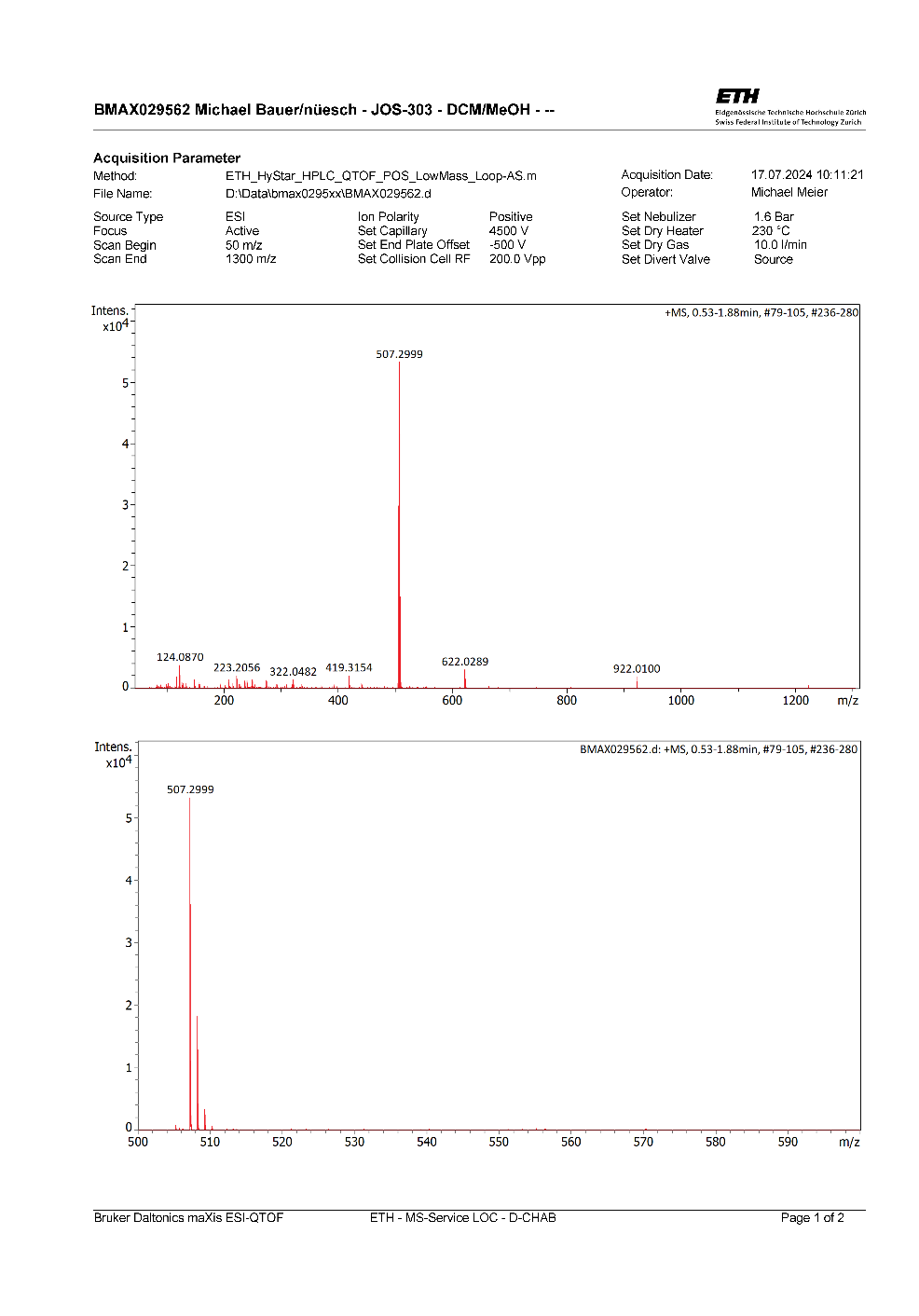

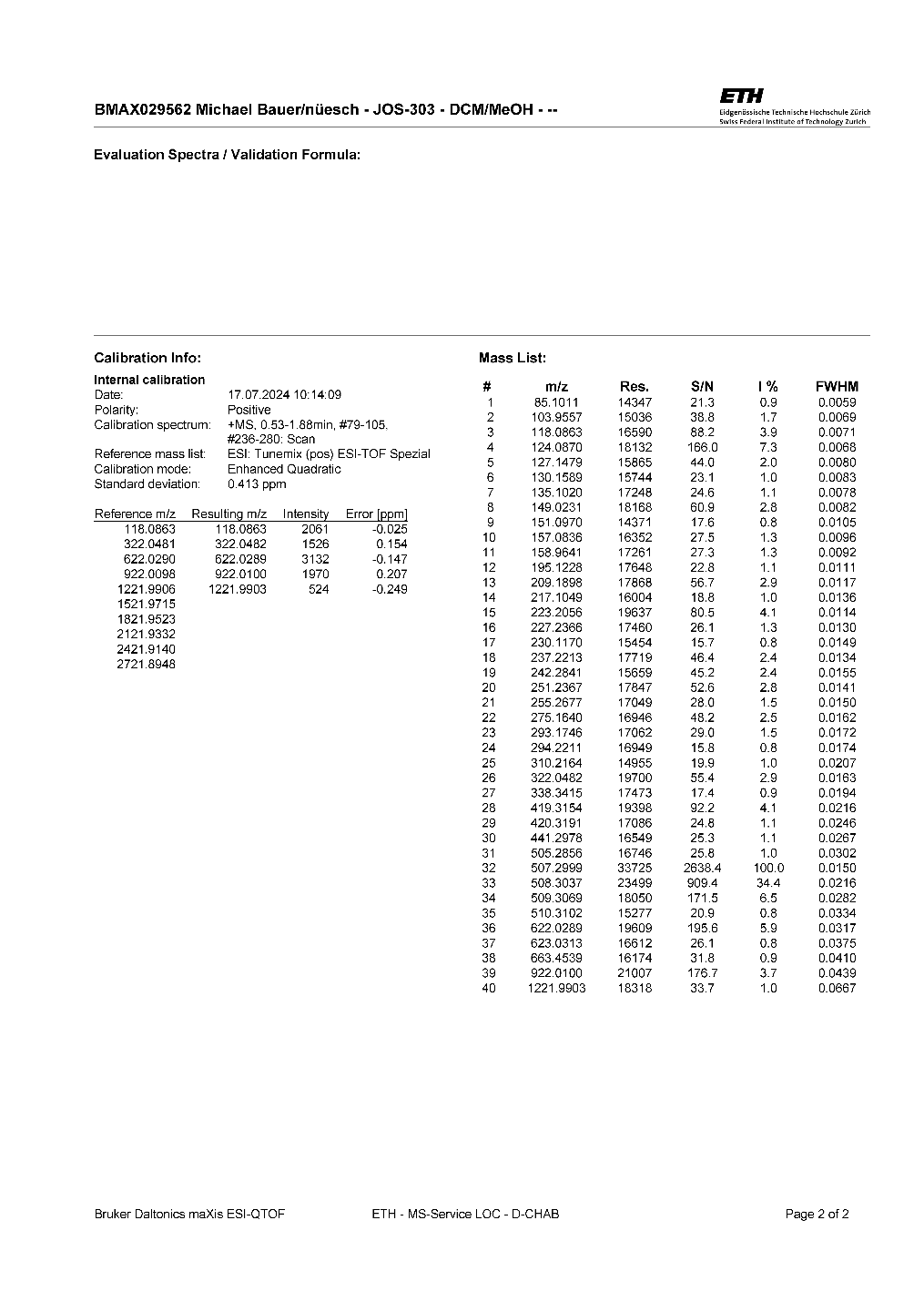


(*+*)-HR-ESI-MS data 4.


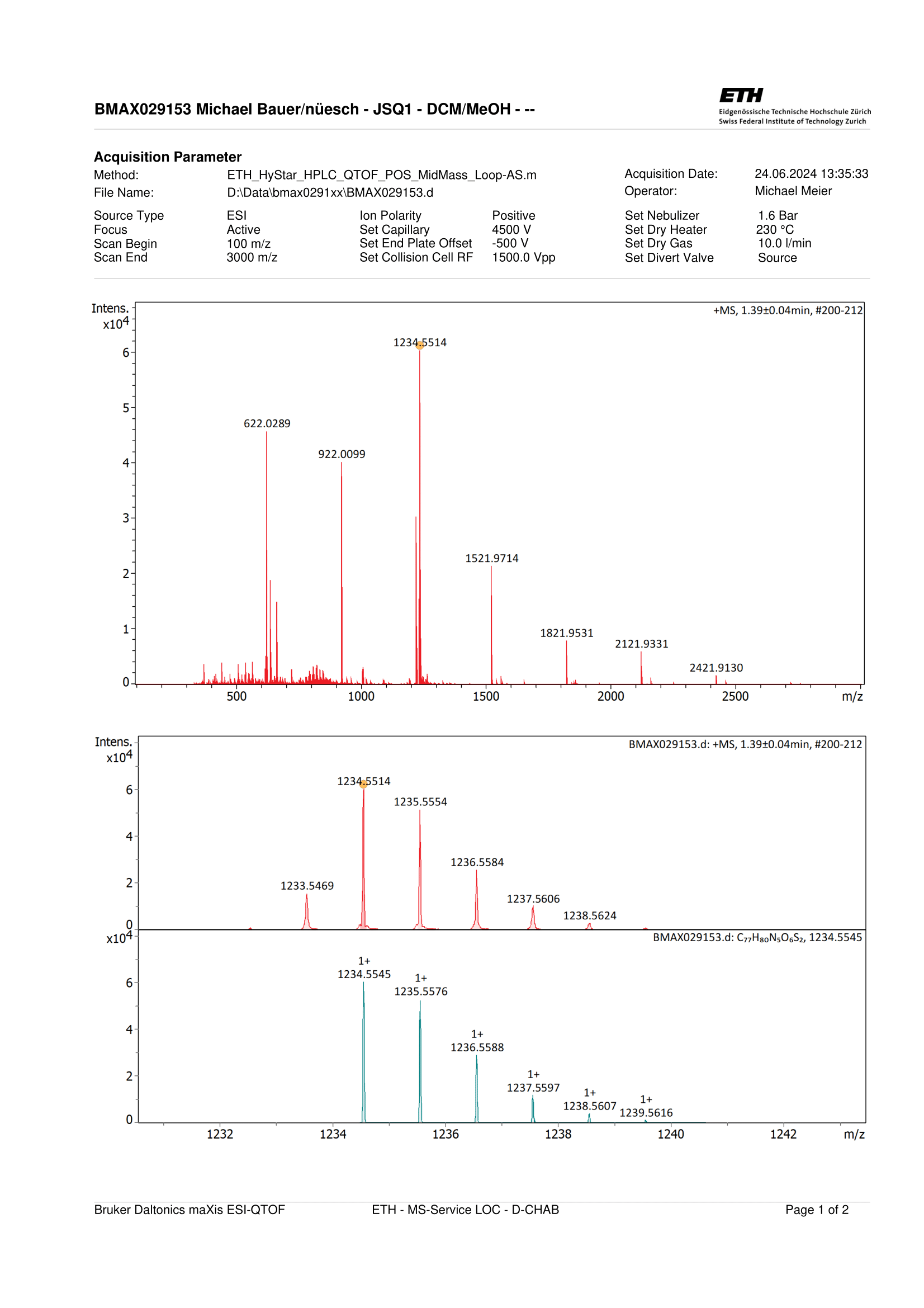

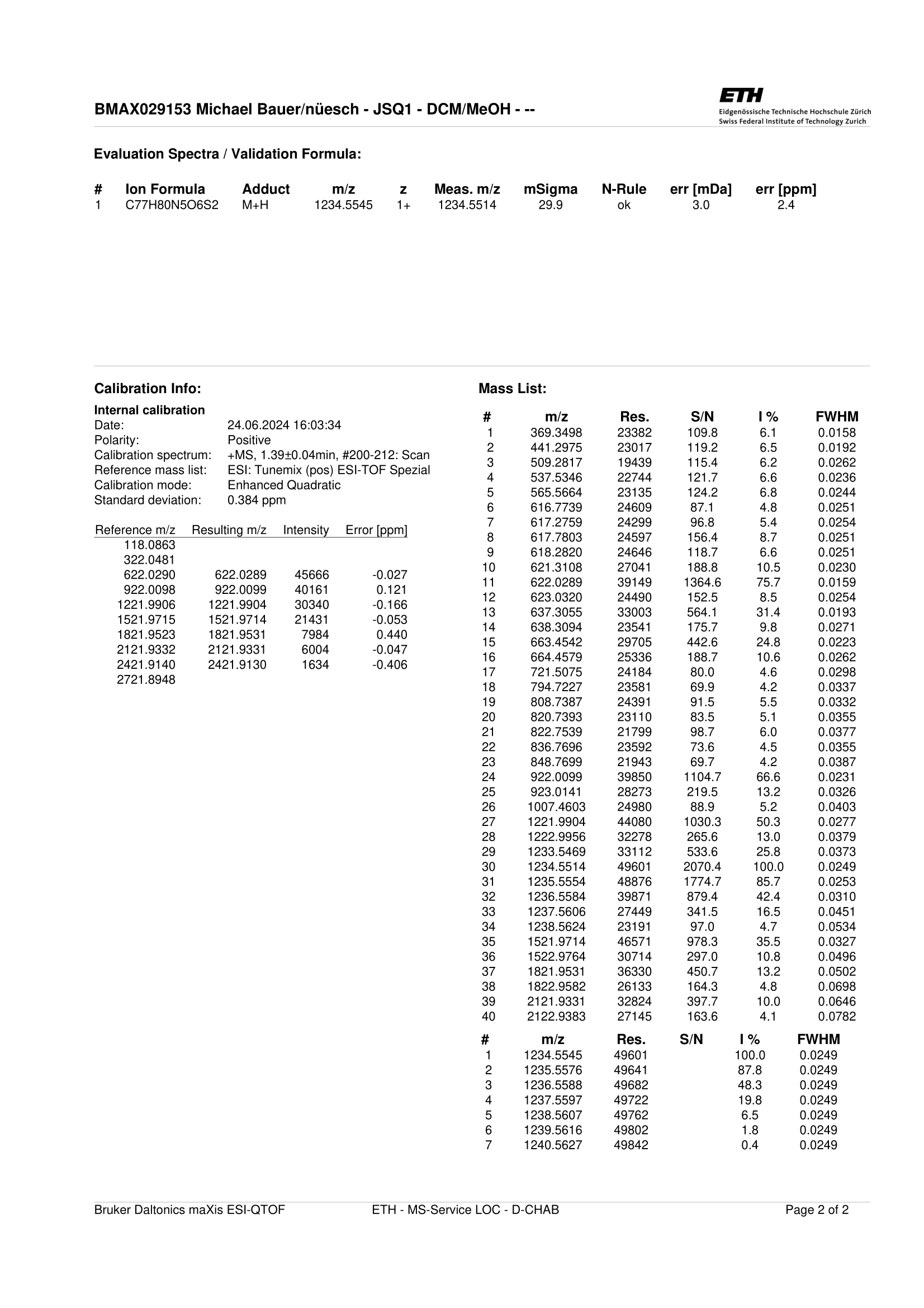


(*+*)-HR-ESI-MS data of RSQ2.


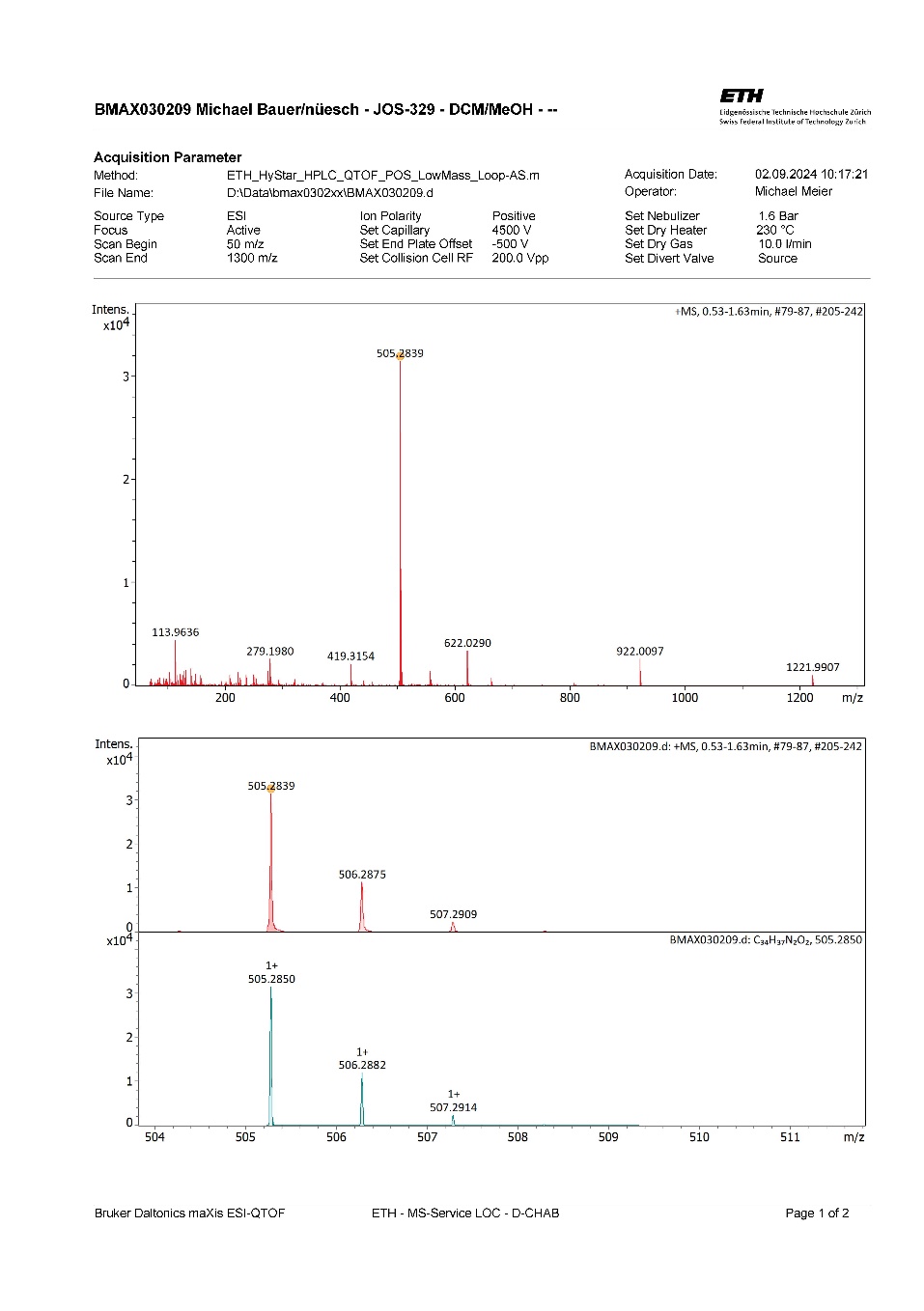

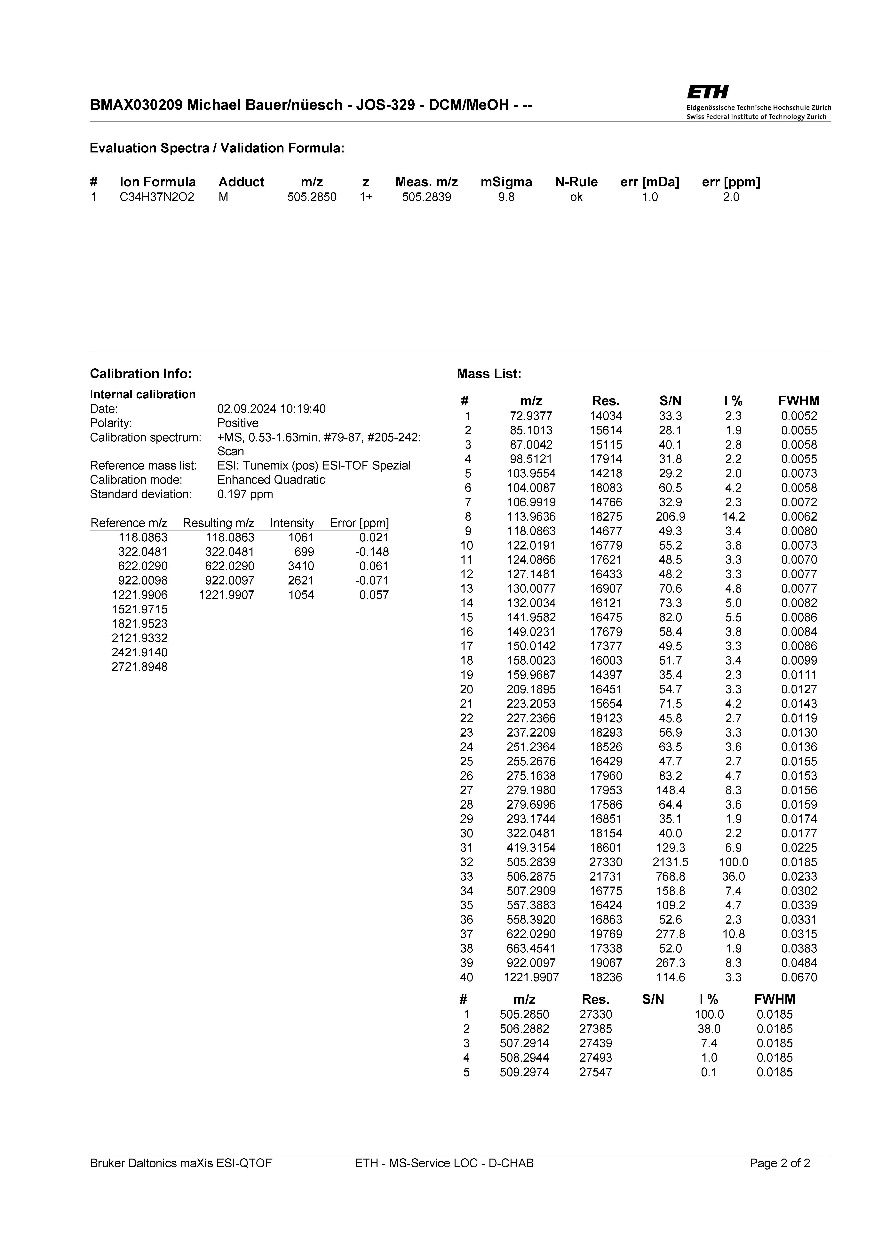


(*+*)-HR-ESI-MS data of 6.


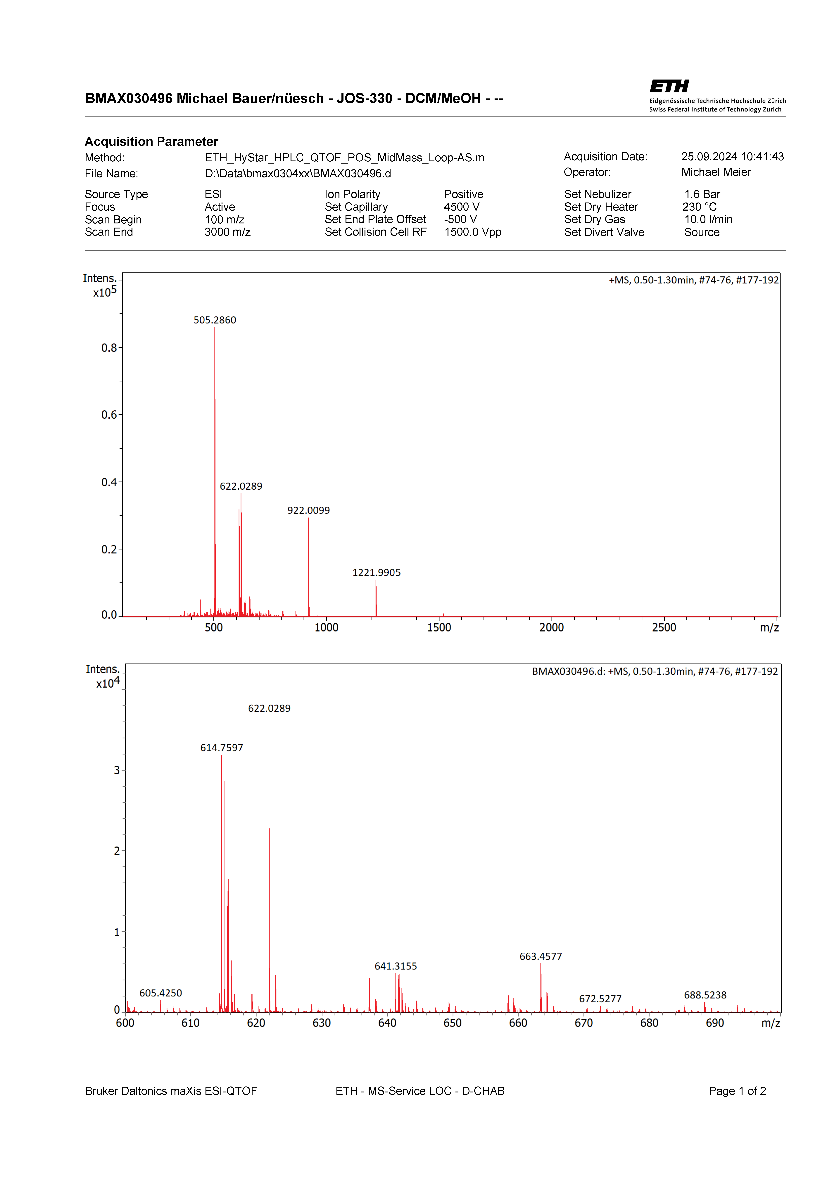

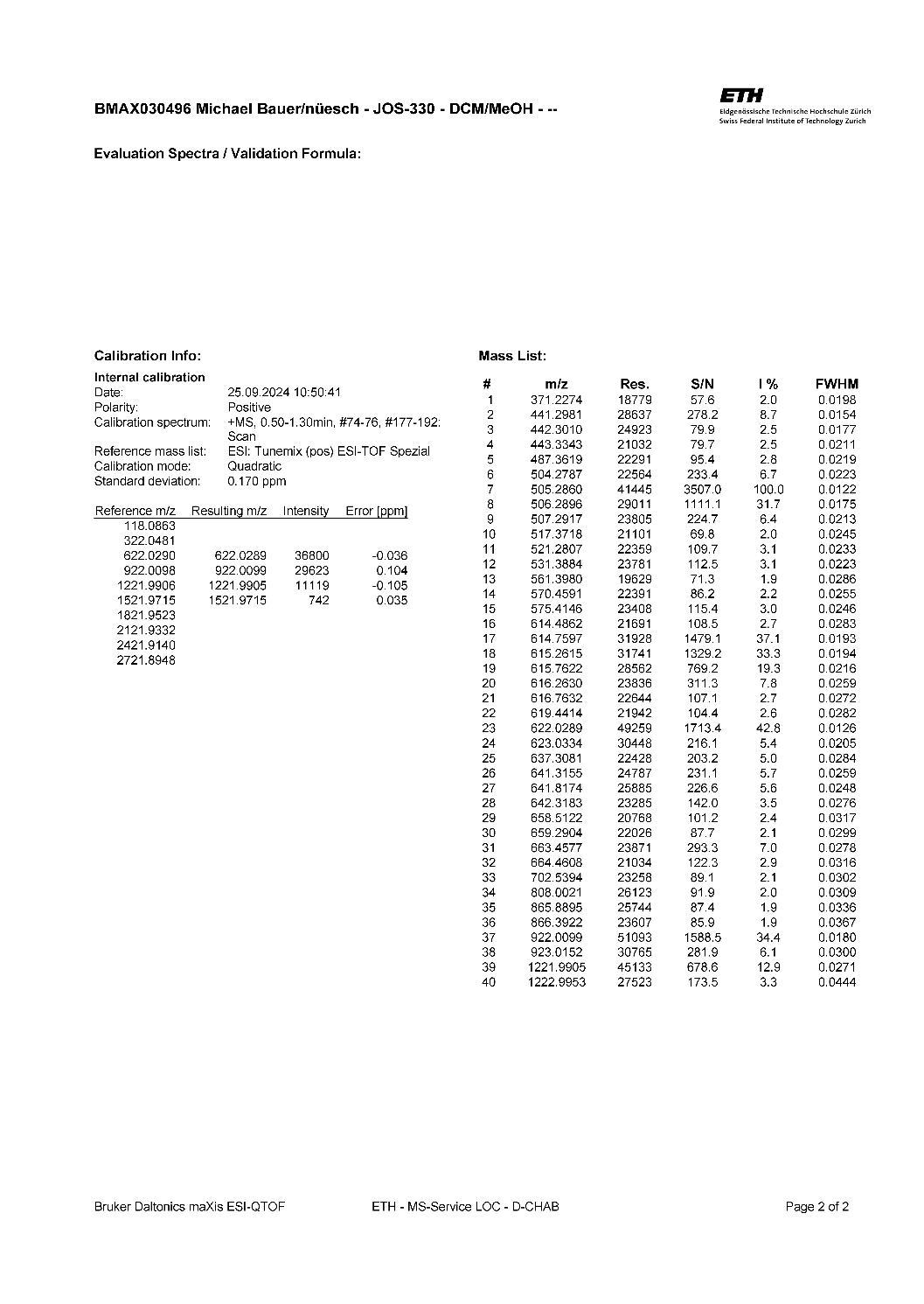


(*+*)-HR-ESI-MS data of **RSQ3**.

**
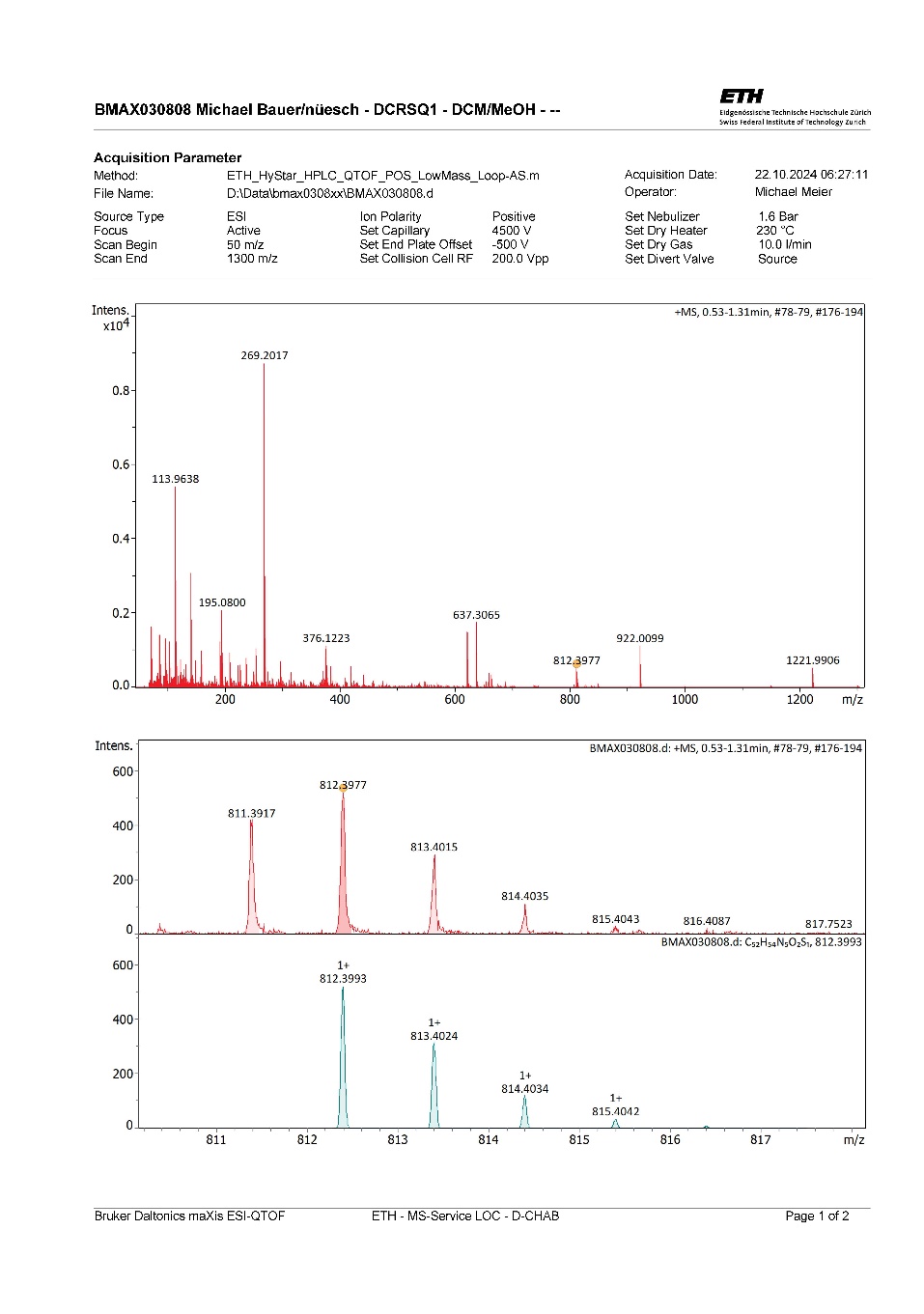

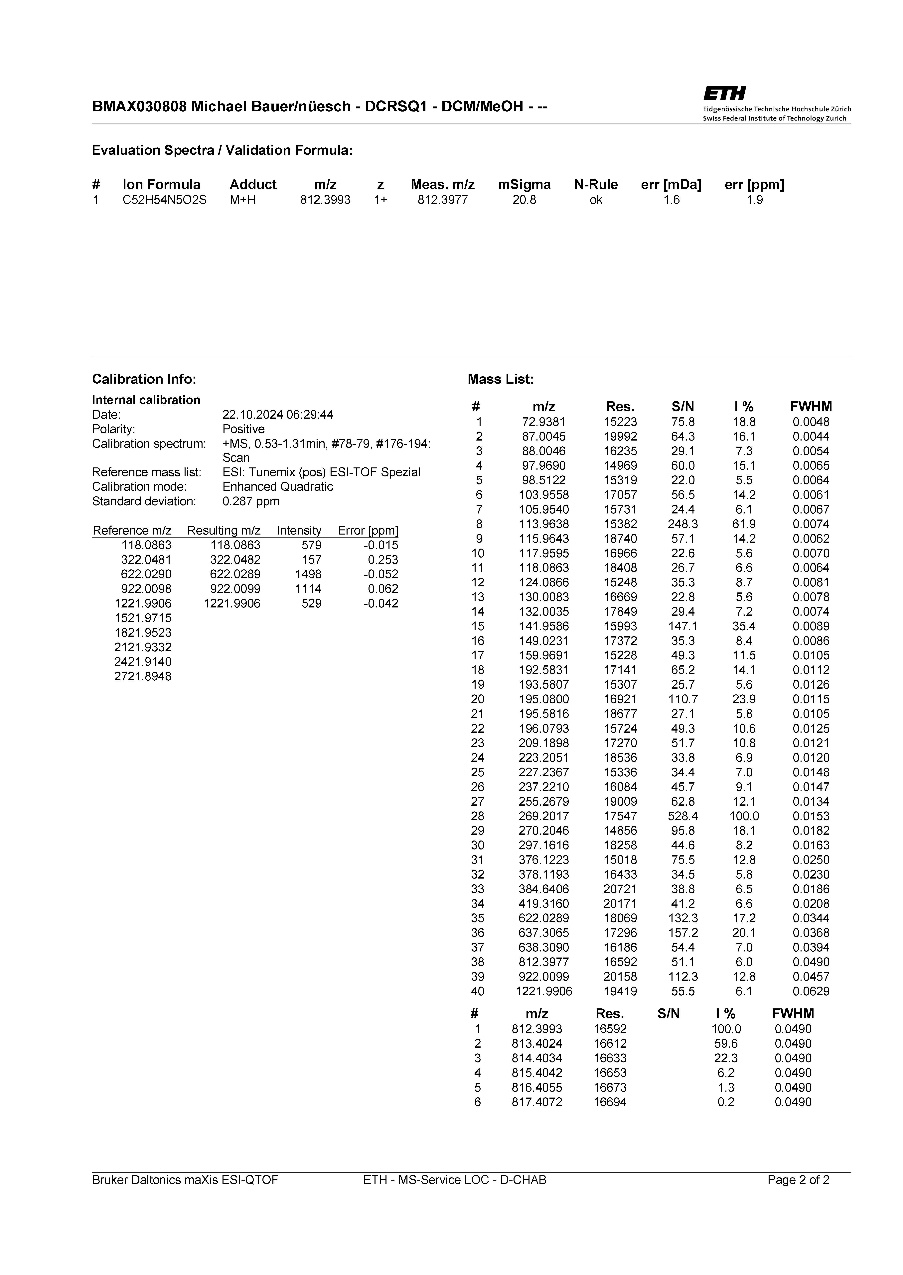
**

(*+*)-HR-ESI-MS data of DCRSQ1.


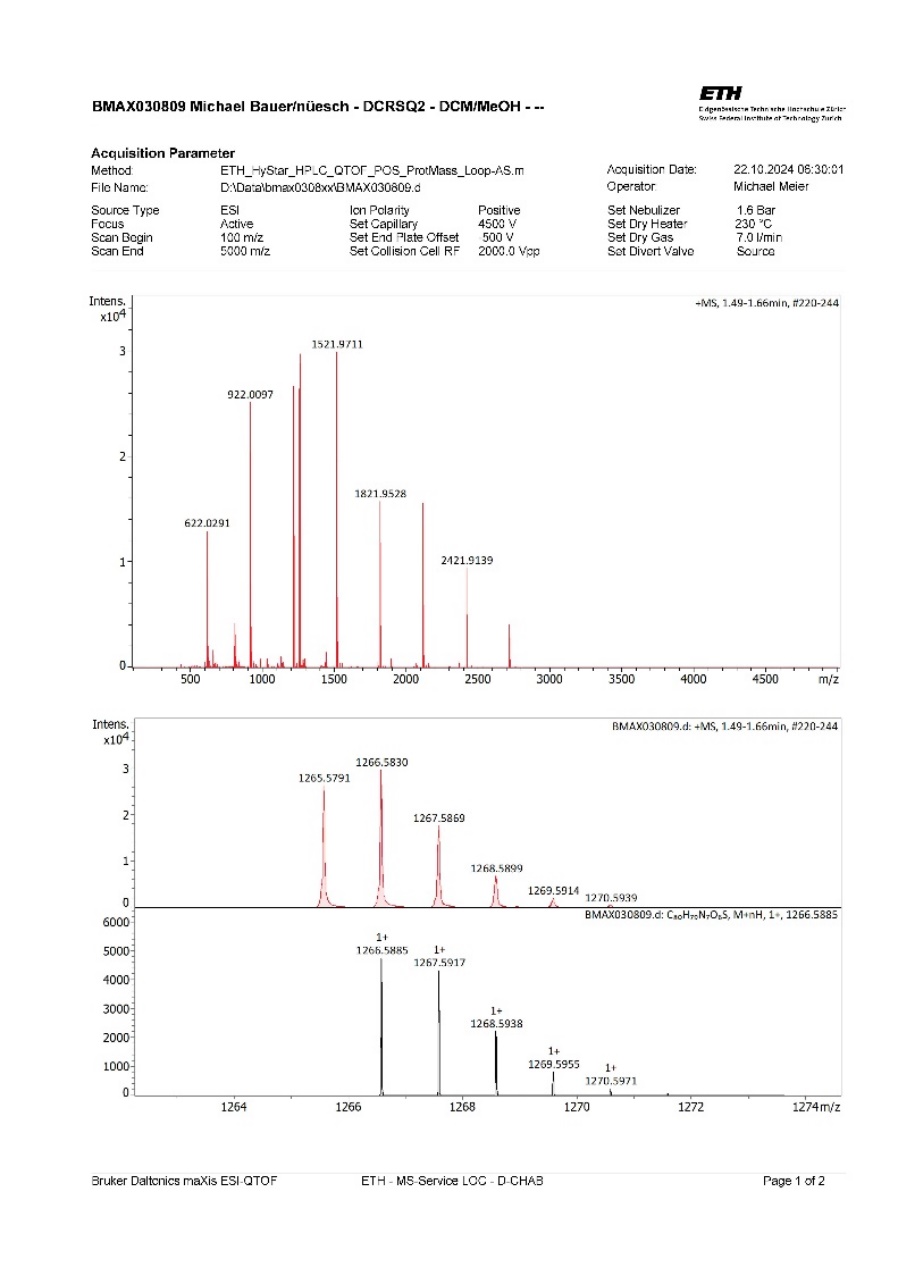

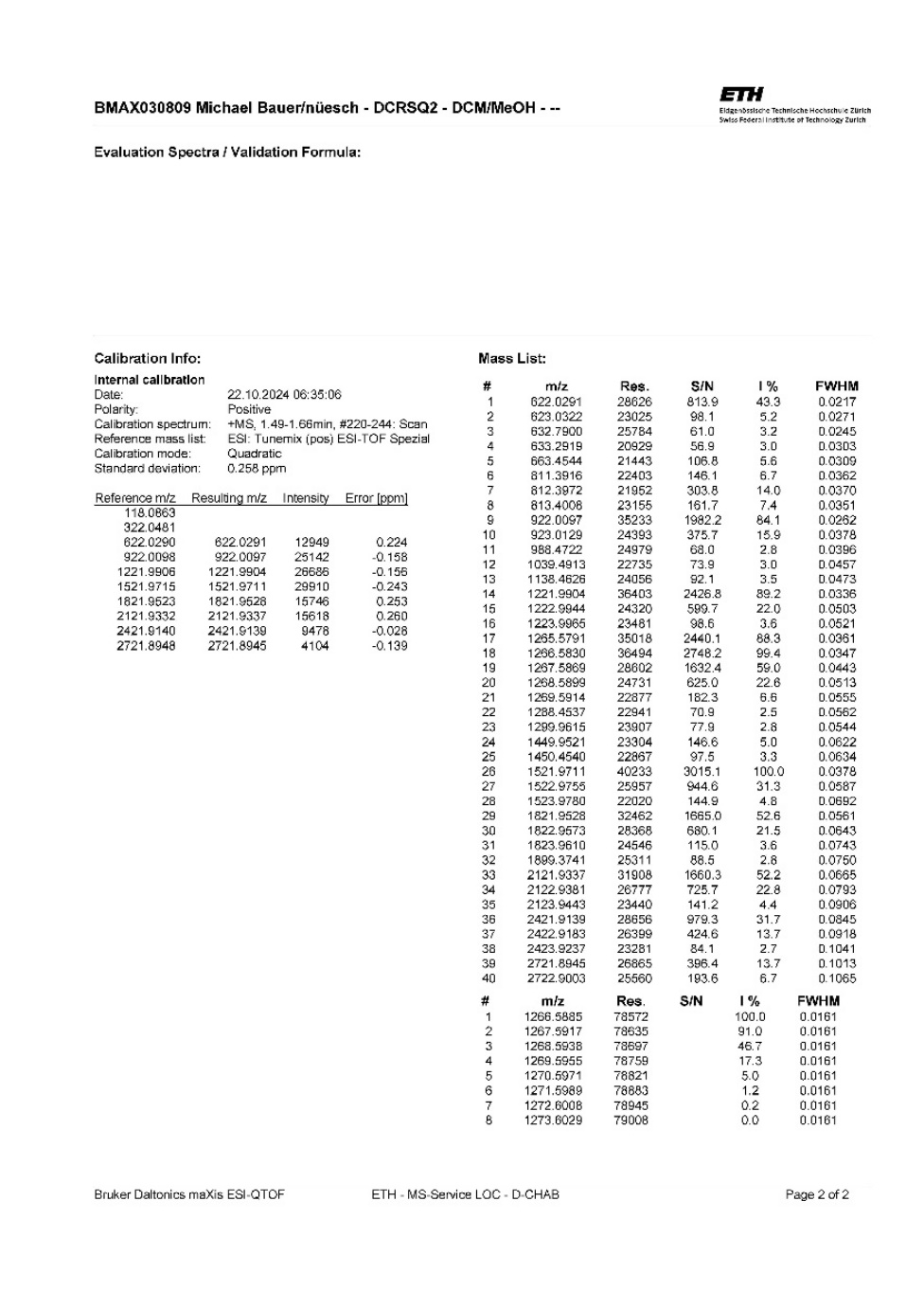


(*+*)-HR-ESI-MS data of DCRSQ2.


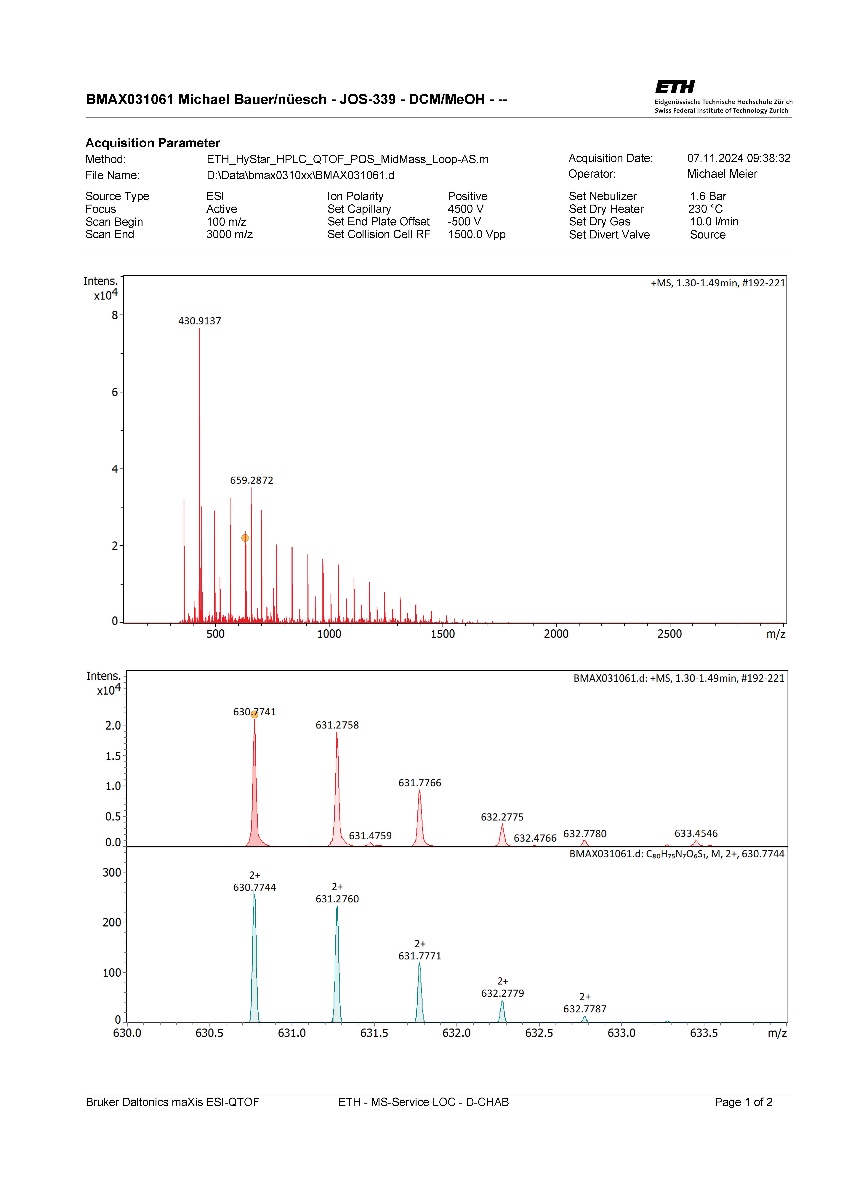

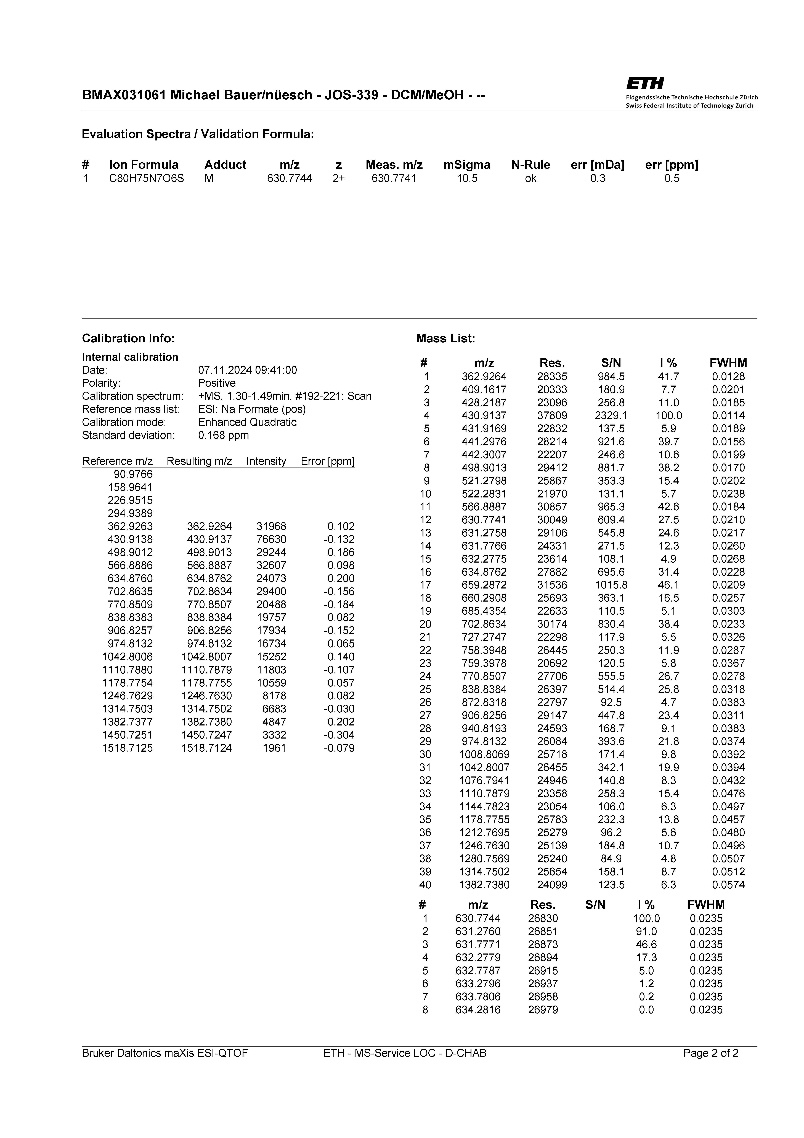


(*+*)-HR-ESI-MS data of DCRSQ3.

**References**

[S1] K. Strassel, W.-H. Hu, S. Osbild, D. Padula, D. Rentsch, S. Yakunin, Y. Shynkarenko, M. Kovalenko, F. Nüesch, R. Hany, M. Bauer, *Sci. Techn. Adv. Mater.* **2021**, *22*, 194.

[S2] C. M. Cardona, W. Li, A. E. Kaifer, D. Stockdale, G. C. Bazan, *Adv. Mater.* **2011**, *23*, 2367.

[S3] F. Neese, *WIREs Comput. Mol. Sci.* **2012**, *2*, 73.

[S4] F. Neese, WIREs Comput. Mol. Sci. **2022**, *12*, e1606.

[S5] F. Weigend, R. Ahlrichs, *Phys. Chem. Chem. Phys.* **2005**, *7*, 3297.

[S6] F. Weigend, *Phys. Chem. Chem. Phys.* **2006**, *8*, 1057.

[S7] Y. Zhao, D. G. Truhlar, *Theor. Chem. Account* **2008**, *120*, 215.

[S8] V. Barone, M. Cossi, *J. Phys. Chem. A* **1998**, *102*, 1995.

[S9] M. D. Hanwell, D. E. Curtis, D. C. Lonie, T. Vandermeersch, E. Zurek, G. R. Hutchison, *J. Cheminf.* **2012**, *4*, 17.

[S10] K. Strassel, A. Kaiser, S. Jenatsch, A. C. Véron, S. B. Anantharaman, E. Hack, M. Diethelm, F. Nüesch, R. Aderne, C. Legnani, S. Yakunin, M. Cremona, R. Hany, *ACS Appl. Mater. Interfaces* **2018**, *10*, 11063.

[S11] H. Zhang, S. Jenatsch, J. De Jonghe, F. Nüesch, R. Steim, A. C. Véron, R. Hany, *Sci. Rep.* **2015**, *5*, 9439.

[S12] H. Zhang, J. Cheng, F. Lin, H. He, J. Mao, K. S. Wong, A. K.-Y. Jen, W. C. H. Choy, *ACS Nano* **2016**, *10*, 1503.

[S13] N. B. Kotadiya, A. Mondal, P. W. M. Blom, D. Andrienko, G.-J. A. H. Wetzelaer, *Nat. Mater.* **2019**, *18*, 1182.

[S14] B. R. Patil, M. Ahmadpour, G. Sherafatipour, T. Qamar, A. F. Fernández, K. Zojer, H.-G. Rubahn, M. Madsen, *Sci. Rep*. **2018**, *8*, 12608.

[S15] N. B. Kotadiya, P. W. M. Blom, G.-J. A. H. Wetzelaer, *Nat. Photonics* **2019**, *13*, 765.

[S16] W.-H. Hu, F. Nüesch, D. Giavazzi, M. Jafarpour, R. Hany, M. Bauer, *Adv. Optical Mater.* **2024**, *12*, 2302105.
